# Silicas with Covalently Anchored Fluorosolvatochromic Dyes Suitable for Naked-Eye Detection of Colourless Compounds

**DOI:** 10.3390/molecules29030673

**Published:** 2024-01-31

**Authors:** Tereza Navrátilová, Martin Havlík, Ameneh Tatar, Karolína Hricková, Bohumil Dolenský

**Affiliations:** Department of Analytical Chemistry, University of Chemistry and Technology, Prague, Technická 5, 166 28 Prague, Czech Republic; navratit@vscht.cz (T.N.); martin.havlik@vscht.cz (M.H.); tatare@vscht.cz (A.T.);

**Keywords:** fluorosolvatochromism, solvatochromic dyes, modified silicas, colorimetric sensors

## Abstract

In this work, two stilbene derivatives with different substituents on the phenolic core (phenyl and dimethoxyphenyl) were prepared. The fluorosolvatochromic response of their *N*-propylated derivatives was studied in a solution of twelve different solvents using UV–Vis absorption and fluorescence emission spectra. Both stilbazolium dyes showed a significant negative solvatochromic effect, with a hypsochromic shift in the visible absorption band of approximately 232 nm and 265 nm for phenyl and the dimethoxyphenyl derivative, respectively, when the solvent was changed from water to pyridine. The stilbene derivatives were subsequently *N*-alkylated with (3-iodopropyl)trimethoxysilane and covalently anchored to the silica surface. The fluorosolvatochromic response of the prepared silicas compared to *N*-propylated dyes was then evaluated colorimetrically under daylight and UV illumination. The fluorosolvatochromic behaviour of the anchored dyes was preserved on the silica surface; therefore, the modified silicas could be used for the visual detection of colourless liquids.

## 1. Introduction

Solvatochromism is the phenomenon observed when the colour of a solution significantly changes when the solute, i.e., the solvatochromic compound, is dissolved in different solvents. In addition, fluorosolvatochromic compounds display a different fluorescence emission when dissolved in different solvents [1]. For decades, the solvatochromic phenomenon has been known and utilised for many applications, such as studying the polarity of solvents [2], detecting polar additives in gasoline [3,4], determining the water content in organic solvents [5] or the pH in nonaqueous solutions [6], or detecting various ions in solution [7,8,9,10].

However, using solvatochromic compounds in solution involves several disadvantages, as the solvatochromic compounds are consumed and the studied solution is contaminated. Therefore, novel materials that can transfer the solvatochromic phenomenon to a solid surface are being developed. The published solvatochromic materials include various polymers [11,12,13,14,15,16,17], silicas [18,19,20,21], organosilanes [22,23], resins [10], organic frameworks [24,25,26] and nanomaterials [27,28,29,30,31]. In this work, we focused on preparing solvatochromic silicas, as silica is a common material used in various industrial and research applications owing to its unique properties, such as chemical inertness, high surface area, porosity, and thermal stability [32].

Various approaches can be used to modify silica surfaces with solvatochromic compounds to form sensing materials. The solvatochromic compound can be bound to the silica surface through chemical bonds or noncovalent interactions. However, covalent binding is preferred because the noncovalent binding of the compound enables leakage [33,34,35].

The process by which organic compounds covalently bind to a silica surface usually includes several steps, such as functionalization of the compound, activation of the silica surface, and their interconnection. Organic compounds can be attached to silica surfaces through various functional groups, depending on the desired application of targeted silica-based materials. A common method to perform covalent silica modifications is to react silanol groups on the surface of silica with an organosilane coupling agent present in the molecule of the organic compound [36].

**Figure 1:**
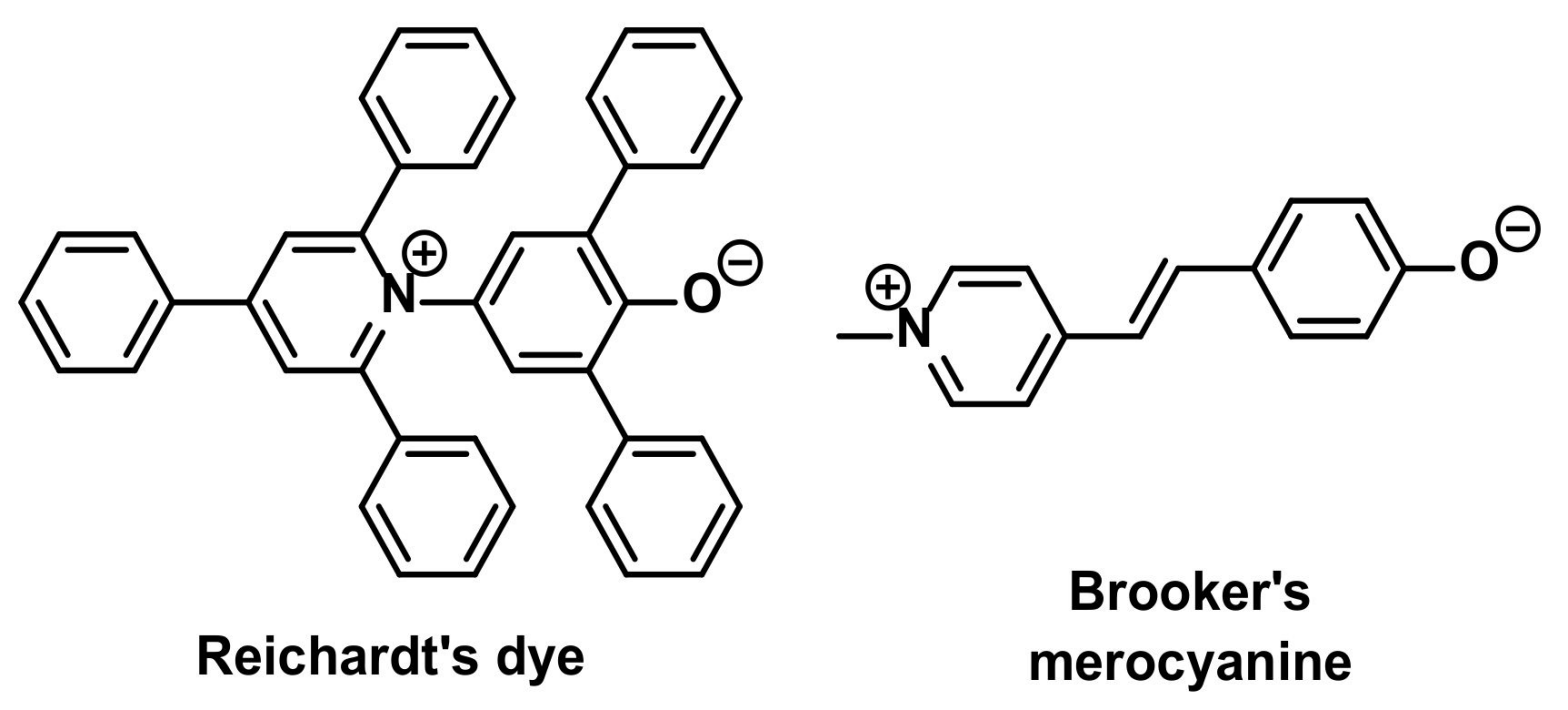
Molecular structures of Reichardt’s dye and Brooker’s merocyanine.

In 1995, Crowther and Liu pioneered the covalent attachment of a solvatochromic compound to a silica surface while retaining its solvatochromic properties [18]. For the solvatochromic compound, the researchers used the famous Reichardt’s dye (Figure 1) [37], also known as ET 30. The compound was modified with an amino group, which was subsequently covalently attached to variously modified silicas. The first material was prepared from isothiocyanatopropyl-modified silica, i.e., connected through a thiourea linker. The second material was prepared from carboxypropyl-modified silica, i.e., connected through an amide linker [18]. The reported work was followed by several others that aimed to use the solvatochromic silicas to construct optical chemical sensors with immobilised dye as a transducer. These sensors should be applicable to the detection of colourless analytes in gaseous and liquid phases [18,19,20,21].

In addition to Reichardt’s dye, Brooker’s merocyanine (Figure 1) is another well-known solvatochromic compound [38,39]. Brooker’s merocyanine dye exhibits one of the largest negative solvatochromic effects, with a hypsochromic shift in the visible absorption band of approximately 137 nm when the solvent is changed from chloroform to methanol [38]. Brooker’s merocyanine is advantageous over Reichardt’s dye because the dye is easy and inexpensive to prepare and maintains a highly pronounced solvatochromic response. In this work, we present a four-step method to prepare silicas modified with two different fluorosolvatochromic derivatives of Brooker’s merocyanine. The method utilizes readily available and cost-effective chemicals, allowing for the large-scale modification of simple chromatography-grade silica without surface activation.

We believe that the modified silicas could be used to construct simple colorimetric sensors that can be evaluated by the naked eye, i.e., autonomous devices, such as public sensors for environmental pollution and chemical hazards, and devices that utilise a flow-through arrangement to monitor the quality of colourless liquids, such as spirits or fuels. Moreover, the silicas could be applied in fields such as construction, art, or cosmetics as chemoresponsive powders, due to the growing interest in the use of smart materials.

## 2. Results and Discussion

### 2.1. Preparation of Model Stilbazolium Dyes **5**

To compare the fluorosolvatochromic behaviour of the dyes in solution and on the solid surface of silica, model compounds *N*-alkylated with a simple propyl group were prepared for fluorosolvatochromic studies in solution.

**Scheme 1:**
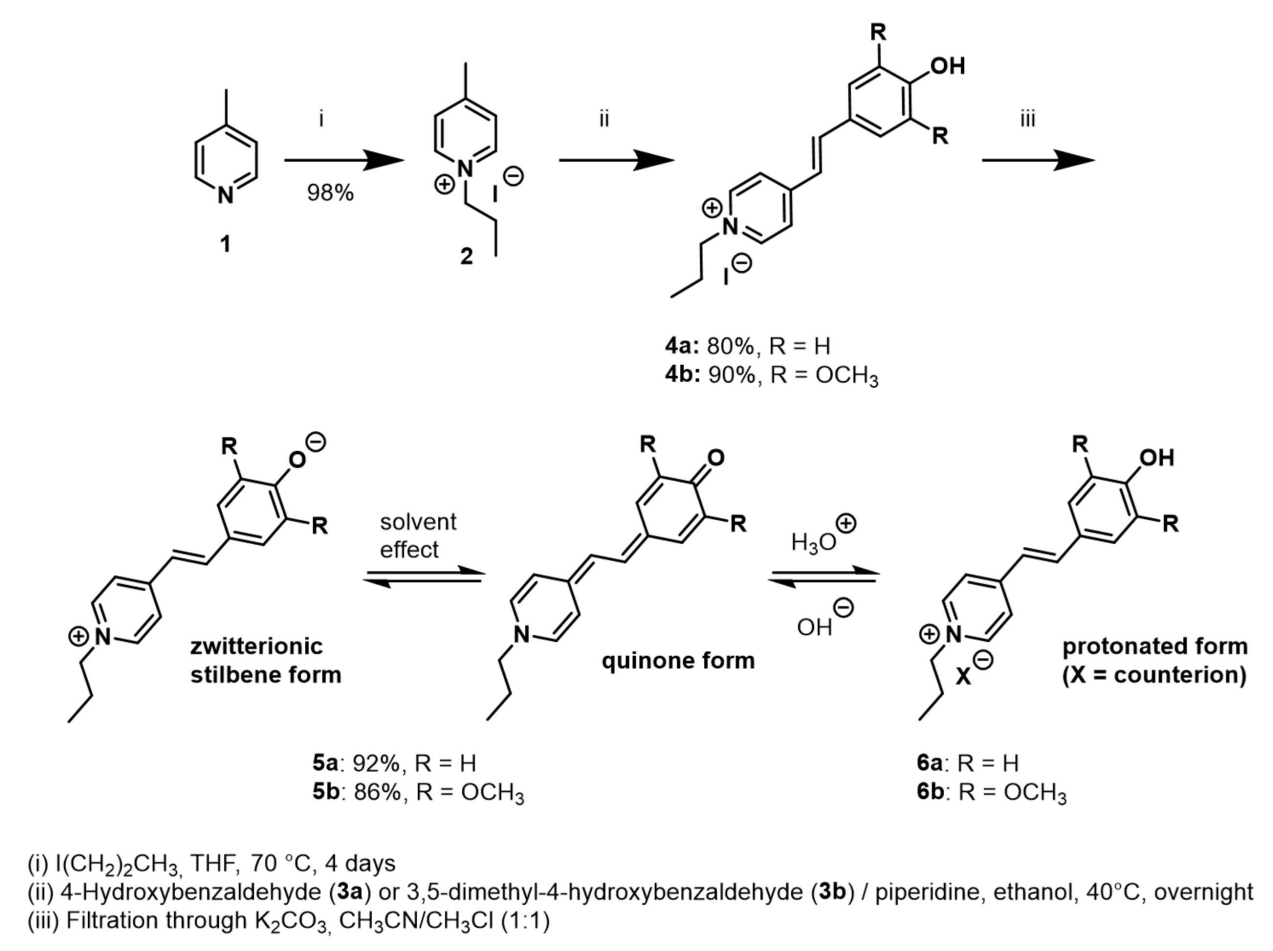
The preparation of model fluorosolvatochromic compounds, stilbazolium dyes **5**, for fluorosolvatochromic studies in solution. The scheme also shows the resonance structures of stilbazolium dyes **5** and their corresponding non-solvatochromic protonated forms **6**.

Model compounds were prepared in three reaction steps (Scheme 1). In the first step, 4-methylpyridine (**1**) was alkylated with 1-iodopropane in tetrahydrofuran to give a 98% yield of pyridinium salt **2** [40]. In the following step, pyridinium salt **2** was treated with 4-hydroxybenzaldehyde (**3a**), or 4-hydroxy-3,5-dimethoxybenzaldehyde (**3b**), and piperidine as a catalyst in ethanol to yield 80% of stilbazolium salt **4a** and 90% of stilbazolium salt **4b**, respectively. In the last step, stilbazolium salts **4** were transformed to the corresponding fluorosolvatochromic form **5** by reaction with potassium carbonate in a mixture of acetonitrile and chloroform to yield 92% of stilbazolium dye **5a** and 86% of stilbazolium dye **5b**.

### 2.2. Fluorosolvatochromic Behaviour of Stilbazolium Dyes **5** in Solution

The fluorosolvatochromic behavior of the prepared model compounds, stilbazolium dyes **5**, was demonstrated by their dissolution in several solvents with different polarities. However, stilbazolium dyes **5** were insoluble in nonpolar solvents, so only a variety of polar solvents were studied.

**Figure 2:**
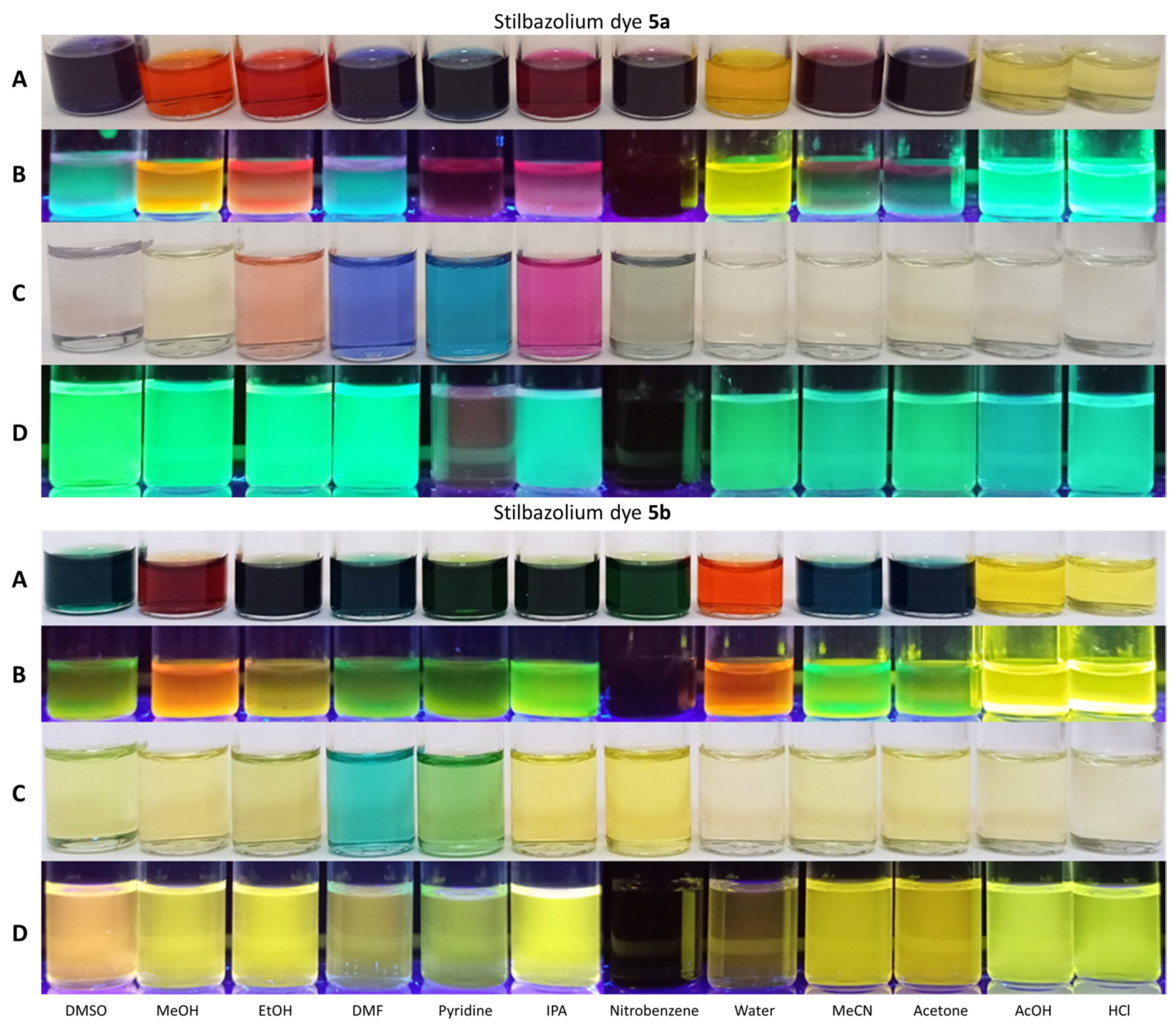
Fluorosolvatochromic response of model compounds **5** to different solvents. (**Top**): The colors of the stock solutions of stilbazolium dye **5a** (c = 0.9402 mmol·L^−1^) under daylight (**A**) and under UV illumination ((**B**), λ_ex_ = 366 nm) and the measured solutions of **5a** (c = 20.38 μmol·L^−1^) under daylight (**C**) and under UV illumination ((**D**), λ_ex_ = 366 nm). (**Bottom**): The colors of the stock solutions of stilbazolium dye **5b** (c = 0.9453 mmol·L^−1^) under daylight (**A**) and under UV illumination ((**B**), λ_ex_ = 366 nm) and the measured solutions of **5a** (c = 20.44 μmol·L^−1^) under daylight (**C**) and under UV illumination ((**D**), λ_ex_ = 366 nm).

First, the fluorosolvatochromic response was studied by simple colorimetric evaluation under daylight and UV illumination (λ_ex_ = 366 nm). The colours of the stock solutions and measured solutions of both compounds are shown in Figure 2. Both stilbazolium dyes showed strong fluorosolvatochromic responses. In the case of stilbazolium dye **5a**, the colour of the solutions ranged from bright yellow (HCl, protonated form **6a**) to shades of red/orange (water, MeOH, zwitterionic form **5a**) to shades of blue/purple (DMF, pyridine, quinone form **5a**). In the case of stilbazolium dye **5b**, the colour of the solutions ranged from bright yellow (HCl, protonated form **6b**) to shades of red/orange (water, MeOH, zwitterionic form **5b**) to shades of blue/green (DMF, pyridine, quinone form **5b**).

**Figure 3:**
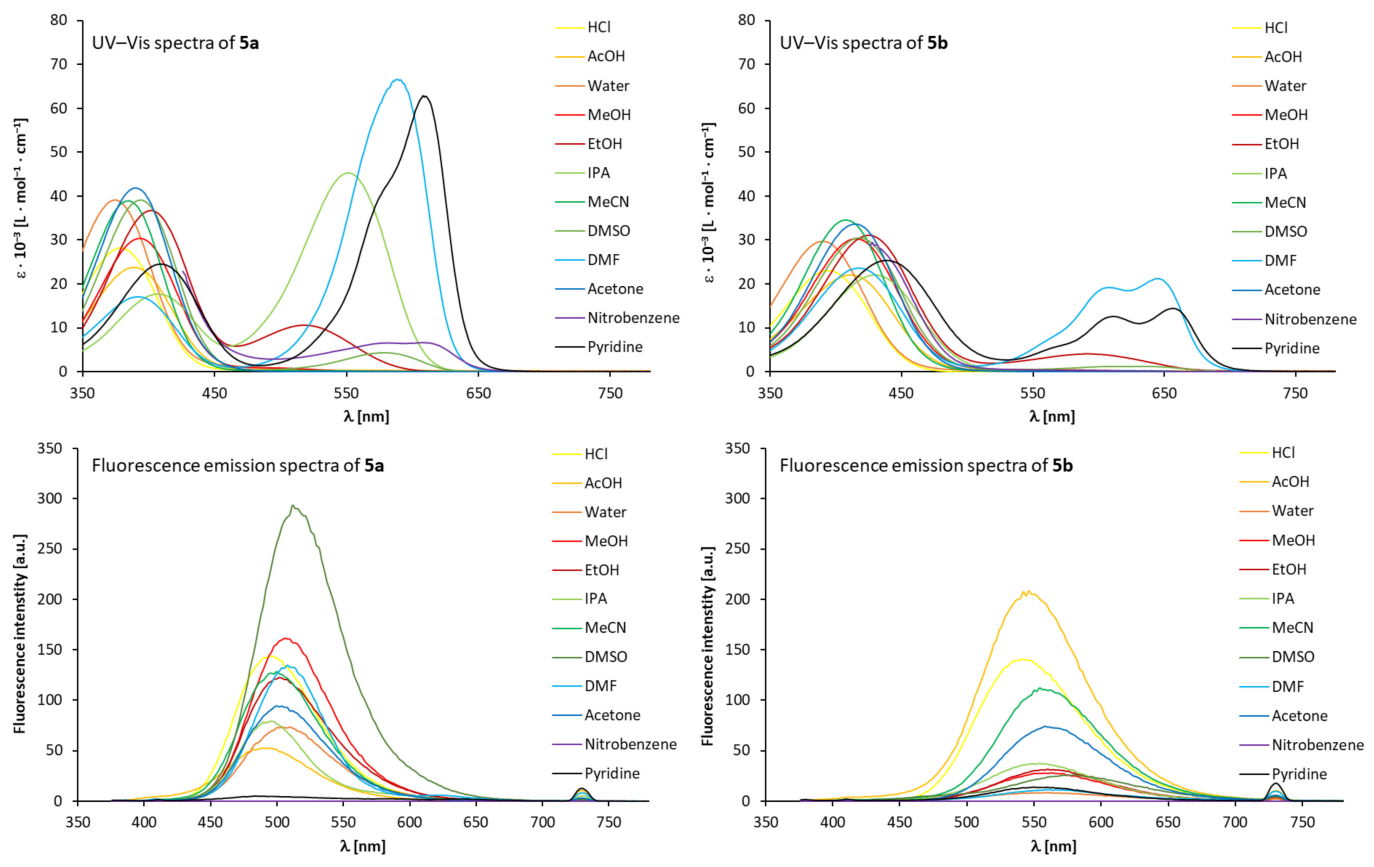
UV–Vis and fluorescence emission (λ_ex_ = 366 nm) spectra of stilbazolium dyes **5**. Top: UV–Vis spectra of stilbazolium dyes **5a** (left) and **5b** (right) measured in different solvents. Bottom: Fluorescence emission spectra of stilbazolium dyes **5a** (left) and **5b** (right) measured in different solvents. The low-intensity band at 732 nm in the fluorescence emission spectra is a second-order band, which is the result of higher-order scattering from the monochromator and is twice the excitation wavelength of 366 nm.

Next, the spectral properties of both stilbazolium dyes **5** were studied using UV–Vis and fluorescence emission spectra of the prepared solutions (Figure 3). An overview of the wavelengths of the maxima of the absorption and emission bands of stilbazolium dyes **5** measured in solvents with different polarities is given in Table 1.

The UV–Vis spectra of both stilbazolium dyes **5** exhibit strong hypsochromic shifts (blue shifts) with increasing solvent polarity, i.e., negative solvatochromism. This finding corresponds with the published spectra of Brooker’s merocyanine [38]. In the case of stilbazolium dye **5a**, the wavelength of the absorption band maxima ranged from 375 nm to 607 nm. The absorption bands at 516–607 nm are prominent in solvents, in which the quinone form of the molecule predominates [38,39]. In the case of stilbazolium dye **5b**, the wavelength of the absorption band maxima ranged from 390 nm to 656 nm. Thus, the spectra of **5b** are redshifted (bathochromic shift) in comparison to those of **5a**. The absorption bands at 592–656 nm are prominent in solvents in which the quinone form of the molecule predominates [38,39].

Moreover, both dyes **5** exhibited fluorosolvatochromism. In the case of stilbazolium dye **5a**, the wavelength of the fluorescence emission band maxima ranged from 490 nm to 512 nm. No fluorescence was observed for nitrobenzene or pyridine. In the case of stilbazolium dye **5b**, the wavelength of the fluorescence emission band maxima ranged from 540 nm to 580 nm. Thus, the fluorosolvatochromic response was stronger for **5b** (40 nm shift) than for **5a** (22 nm shift).

**Figure 4:**
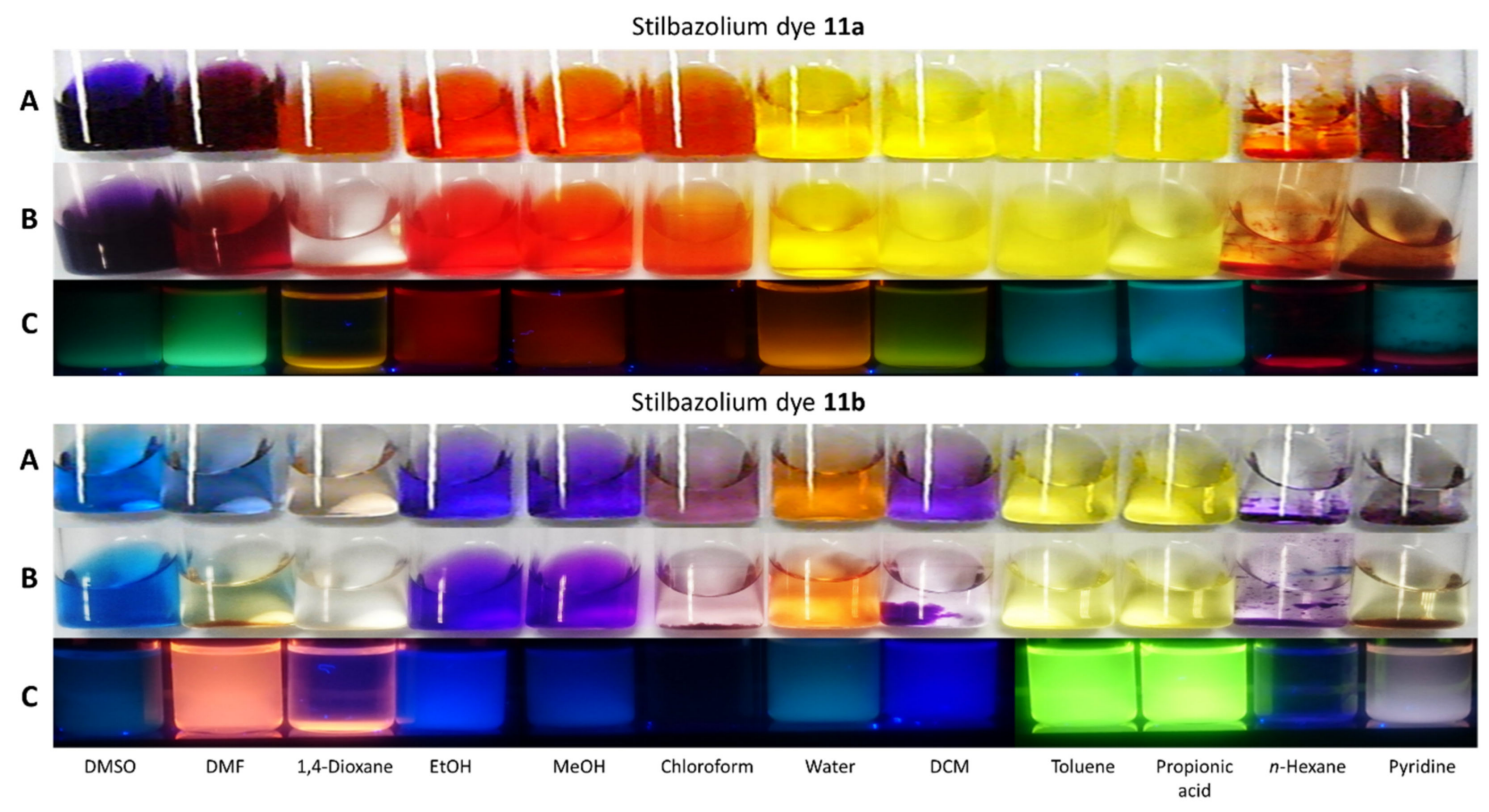
Fluorosolvatochromic response of model compounds **11a** (**top**) and **11b** (**bottom**) to different solvents under visible light ((**A**): freshly prepared solutions, (**B**): solutions after 24 h) and under UV illumination ((**C**): λ_ex_ = 366 nm).

We also studied model compounds *N*-alkylated with a silane anchor, i.e., stilbazolium dyes **11**. The preparation of stilbazolium dyes **11a** and **11b** is given in the Supplementary Materials. Solutions of stilbazolium dyes **11** in methanol were tested for fluorosolvatochromic behavior without further purification because of the poor stability of the prepared compounds. In the vials, 50 μl of the prepared stilbazolium dyes **11** solution was mixed with 3 mL of the studied solvent. The colour change of stilbazolium dyes **11a** and **11b** depending on the solvent used can be seen in Figure 4. It follows that the prepared stilbazolium dyes show fluorosolvatochromic behavior. However, the resulting colour of the solutions of both dyes is influenced by impurities from the preparation of both compounds, i.e., methanol, sodium hydroxide, sodium iodide, sodium acetate, and (3-iodopropyl)trimethoxysilane. The figure also shows that the prepared salts were not soluble in some solvents (*n*-hexane, pyridine). Even in these solvents, however, the colour of the dye varied. Their fluorescence (λ_ex_ = 366 nm) was most pronounced in solutions of *N*,*N*-dimethylformamide, toluene, and propionic acid.

However, the solutions of the prepared compounds were not stable. In Figure 4, freshly prepared solutions are compared with solutions after 24 h. The figure shows that in some cases there was a slight colour change, and in other cases there was a separation of the dye particles and the solvent taking place, whereby the separated particles could not be re-dissolved by stirring the solution. In most cases, aggregation of microparticles in the solution, their settling at the bottom, or excretion on the walls of the vial were observed. The particles of the compounds were separated to form a suspension within minutes to hours (depending on the solvent used). The figure shows that in the case of **11b**, the *N*,*N*-dimethylformamide solvent, there was a significant colour change, which can be explained by the separation of the *N*,*N*-dimethylformamide and methanol phases since the colour of the solid at the bottom of the *N*,*N*-dimethylformamide flask corresponds to the colour of the solution of dye **11b** in methanol. Thus, degradation of compounds occurred, probably due to hydrolysis of the silane anchor. Stilbazolium dyes **11** were therefore not suitable as model compounds and were excluded from our investigation.

### 2.3. Preparation of Fluorosolvatochromic Silicas 10

**Scheme 2:**
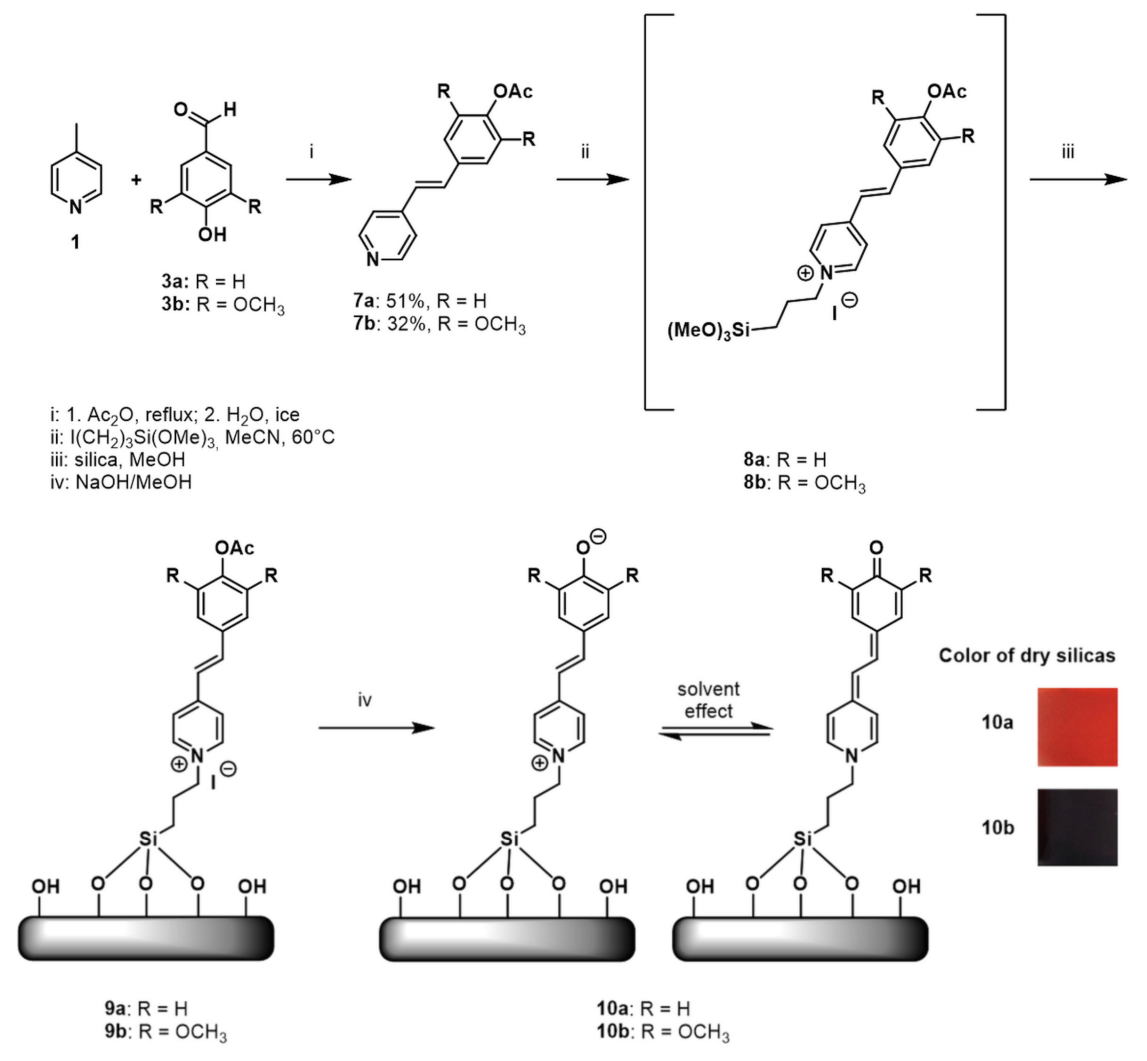
The preparation of modified silicas **10**. The scheme also shows the resonance structures of the anchored stilbazolium dyes and the colour of the dry modified silicas **10**.

The preparation of modified silicas took place in four reaction steps (Scheme 2). In the first step, the aldol condensation of 4-methylpyridine (**1**) and the corresponding benzaldehyde derivative **3** yielded 51% of stilbazole **7a** and 32% of stilbazole **7b**, respectively. In the second step, stilbazoles **7** were *N*-alkylated with (3-iodopropyl)trimethoxysilane to yield stilbazolium salts **8**, which were used without isolation due to the poor stability of the trimethoxysilane anchor. In the third step, stilbazolium salts **8** were covalently attached to the solid surface of chromatography-grade silica via a trimethoxysilyl group in a mixture of dichloromethane and methanol (19:5 *v*/*v*). The suspension was left to stand for 12 h with occasional mixing. After the solvent was evaporated, modified silicas **9** bearing acetylated stilbazolium dyes were obtained. The prepared silicas were dried under reduced pressure, washed on frit with water and methanol, and dried again. In the fourth step, the phenolic groups of the attached stilbazolium dyes were deacetylated in a basic solution of 0.5 M sodium hydroxide in methanol, resulting in the formation of fluorosolvatochromic silicas **10**. The prepared silicas were washed on frit with water and various organic solvents, subsequently washed with diethyl ether, dried by air, and then dried under reduced pressure to dryness. The colour of the dry silicas is shown in Scheme 2.

The covalent attachment of the compounds to the silica surface was demonstrated by the absence of any coloured substance being washed out of the silicas when the samples were rinsed on the frit with various solvents, including chloroform, dimethyl sulfoxide, and water. We further characterised the prepared silicas using elemental analysis (see the Supplementary Materials). We determined the amount of bound stilbazolium dye using the measured amount of nitrogen. For silica **10a**, the modification yield was determined to be 5.0 μmol, and for silica **10b**, the modification yield was 5.8 μmol of stilbazolium dye bound on the surface. The amount of dye bound to the surface was therefore very low in both cases, demonstrating the robustness of the colour response of both dyes. Moreover, with such a small amount of indicator, the preparation of the silicas is inexpensive. Importantly, we attempted to prepare another batch of modified silicas, and the method, including the fluorosolvatochromic response, was reproducible.

Since we used acetylated stilbazoles to modify the silicas, we wanted to further verify that deacetylation occurs quantitatively under the given conditions. Furthermore, we wanted to compare the fluorosolvatochromic response of stilbazolium salts before and after acetyl deprotection using UV–Vis spectra (Figures S66 and S67). For this comparison, we used a model system in solution. The details of the experiments, including the preparation of the compounds and the UV–Vis spectra, are given in the Supplementary Materials. The results confirmed that complete deacetylation occurred under the given reaction conditions. The experiments further showed that deacetylation partially occurs in basic solvents, such as *N*,*N*-dimethylformamide.

### 2.4. Fluorosolvatochromic Properties of the Prepared Silicas

**Figure 5:**
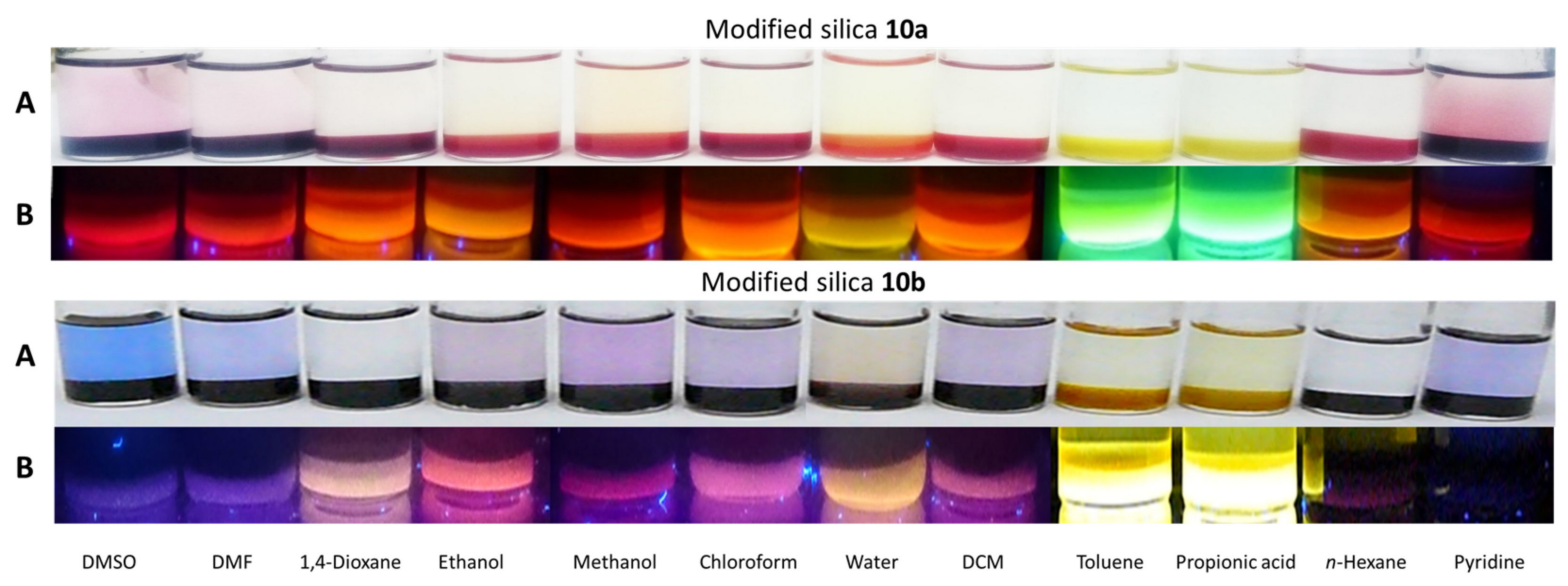
Fluorosolvatochromic response of modified silicas **10a** (**top**) and **10b** (**bottom**) at the bottom of the vials in solvents of different polarities under daylight (**A**) and UV illumination ((**B**), λ_ex_ = 366 nm).

The fluorosolvatochromic behaviour of the prepared silicas **10** was studied in twelve different solvents. Approximately 200 mg of the prepared silica was weighed into each vial, and 3 mL of the tested solvent was added. The resulting colour change in modified silicas **10** caused by the added solvent is shown in Figure 5.

The modified silicas showed rapid and significant colour changes depending on the solvent added. Therefore, the solvatochromic response of the anchored dyes was preserved after they were covalently attached to the surface of silica.

Figure 5 further shows that both modified silicas exhibited strong fluorosolvatochromic behaviour. Their fluorescence under UV illumination (λ_ex_ = 366 nm) was most pronounced in toluene and propionic acid solutions. Fluorescence in the presence of acid is not surprising since fluorescence of the protonated form was also observed for model compounds **5**. However, the fluorescence in the presence of toluene was surprising. The similarity between toluene and acid is, in our opinion, peculiar since the two solvents differ dramatically in their molecular structure, pKa, polarity, and other properties. Since model compounds **5** are not soluble in toluene, this solvent cannot be used for comparison.

Therefore, to compare with the solid phase, we also aimed to study aromatic solvents, in which stilbazolium dyes **5** were not soluble. We studied the effects of both solvents by measuring their UV–Vis and fluorescence emission spectra in solvents (benzene and toluene) mixed with solutions of the studied stilbazolium dyes **5** in dimethyl sulfoxide (less polar) or in methanol (more polar). For further information about the experiments, including UV–Vis and fluorescence emission spectra and colorimetric evaluation, see the Supplementary Materials. Experiments showed that fluorescence was significantly suppressed in the presence of aromatic solvents when dimethyl sulfoxide was introduced; however, fluorescence was preserved in the presence of methanol. Therefore, we believe that free hydroxyl groups on the surface of silica play a significant role in the observed fluorescence of silicas **10** in the presence of aromatic solvents.

**Figure 6:**
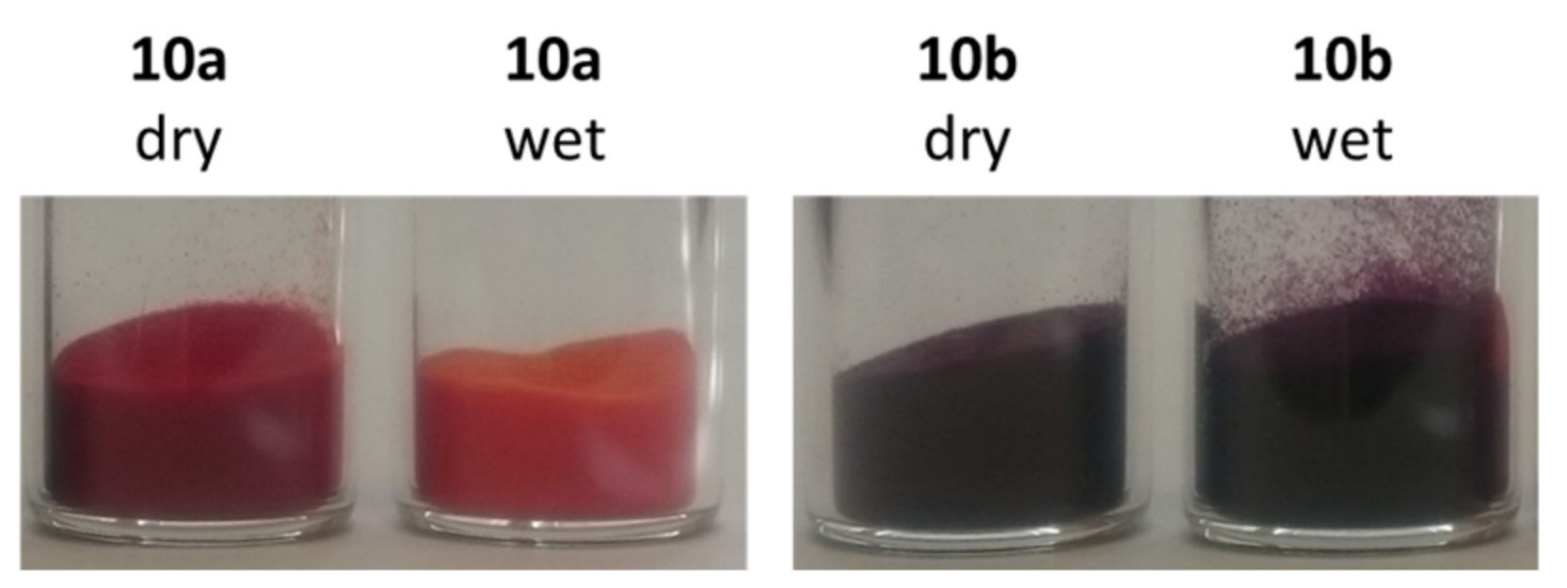
Solvatochromic response of silicas **10** to change in humidity.

Next, we investigated the response of modified silica **10a** in a flow-through arrangement. We fixed a layer of **10a** in a glass tube using glass wool. With this arrangement, we washed the tube with a series of solvents. The colour response was continuous and observable with the naked eye (see Supplementary Materials, Figure S68). Finally, in addition to the fluorosolvatochromic response, both silicas also exhibited a fluorovapochromic response. The response of solvatochromic silicas **10** to changes in humidity was evaluated colorimetrically. Figure 6 shows photos of freshly dried silicas and silicas after 24 h exposure to the air. It can be seen from the figure that in both cases, the sorption of atmospheric moisture took place, as the colour of the materials corresponds to their colour in suspension with water. The materials can therefore be used for several applications, such as simple indicators of relative humidity.

## 3. Materials and Methods

### 3.1. Chemicals and Materials

All reagents were purchased from commercial providers and used without further purification. For the modification of silicas, chromatography grade silica with a 60 Å pore size and 40–63 μm particle size (Material Harvest, Cambridge, UK) was used.

### 3.2. Nuclear Magnetic Resonance

NMR spectra were recorded with a JNM-ECZ500R NMR spectrometer (JEOL Resonance, Tokyo, Japan) operating at 500.16 MHz for ^1^H and 125.77 MHz for ^13^C. The ^1^H NMR and ^13^C NMR spectra were referenced to the residual solvent signal of DMSO-*d*_6_ (^1^H NMR: 2.50 ppm; ^13^C NMR: 39.52 ppm). The chemical shifts (*δ*) are given in ppm. The coupling constants (*J*) are given in Hz. All the spectra were measured at 25 °C.

### 3.3. High-Resolution Mass Spectrometry

Mass spectrometry was performed with an LTQ Orbitrap Velos mass spectrometer (Thermo Scientific, Waltham, MA, USA), a hybrid Ion trap-Orbitrap spectrometer with an Ion Max ion source, and an H-ESI II probe. The conditions for MS detection were as follows: ionization, ESI^+^; spray voltage, 3.0 kV; source temperature, 250 °C; capillary temperature, 300 °C; measurement mode, FTMS; resolution (FWHM), 30,000; lock mass, 413.2662 Da (diisooctyl phthalate); and scan range, 150–2000 Da. The samples were dosed under direct injection conditions (FIA) using an Accela 600 (Thermo Scientific) pump. The conditions for the injection were as follows: sample dosage, 5 μL (injection loop, Rheodyne valve); mobile phase, methanol; and flow rate of the mobile phase, 150 μL/min.

### 3.4. Elemental Analysis

Elemental analyses were performed on an Elementar Vario EL Cube CHNS elemental analyser (Elementar, Langenselbold, Germany). The precision was determined by the manufacturer to simultaneously analyse 5 mg of the standard of 4-aminobenzene sulfonic acid in the CHNS module to be <0.1% absolute (homogeneous substance), depending on sample type, analysis mode, and configuration for each element.

### 3.5. UV–Vis Spectroscopy

Absorption spectra were measured in 1 cm optical quartz cuvettes (Aireka Cells, Hong Kong, China) at ambient temperature on a Cary 60 UV–Vis spectrometer (Agilent Technologies, Santa Clara, CA, USA). The spectra were measured over the spectral range of 190–1100 nm. The spectrum of the measured solvent was used for baseline correction. The molar absorption coefficients of the measured substances were determined via linear regression from dilution experiments. The measured spectra are shown as plots of the molar absorption coefficients versus wavelength. The given spectra were obtained by dividing the measured spectra from the dilution experiments by the measured concentrations. For spectra measured in a single solvent, the spectra obtained were averaged. When solvent (e.g., nitrobenzene) absorption was high, the relevant parts of the spectrum were clipped. The preparation of the samples for the measurements and the resulting measured concentrations are given in the Supplementary Materials.

### 3.6. Fluorescence Spectroscopy

Fluorescence emission spectra were measured in a 1 cm optical quartz cuvette (Aireka Cells, Hong Kong, China) on a Cary Eclipse fluorescence spectrometer (Agilent Technologies). Fluorescence emission spectra were evaluated without blank subtraction. The excitation wavelength used was 366 nm, unless stated otherwise. The spectra were measured in the spectral range of 376–900 nm, unless stated otherwise. Other parameters were set as follows: excitation slit, 5 nm; emission slit, 5 nm; scan rate, 1200 nm/min; averaging time, 0.1 s; and data interval, 2.0 nm. More information, the preparation of samples for measurement, and the resulting concentrations are given in the Supplementary Materials.

### 3.7. Optical Images

Optical images were captured using an ordinary mobile phone with a M2003J15SC camera (Xiaomi, Beijing, China). The camera settings and parameters of the images taken were as follows: photo dimensions in pixels: 4000 × 2992; horizontal and vertical resolution, 96 dpi; bit depth, 24; sRGB colour resolution; aperture shutter, f/1.8; ISO 108; exposure duration, 1/100 s; exposure metre mode with centre emphasis; focal distance, 5 mm; without flash; and automatic white balance. The images were processed only by cropping, and no other adjustments (brightness, contrast, etc.) were made. The images were taken under constant lighting. A manual UV lamp with a power of 4W and λ_ex_ = 366 nm was used for UV illumination.

## 4. Conclusions

In this work, we prepared two novel silica-based solids covalently modified with fluorosolvatochromic stilbazolium dyes, which enabled colour changes based on the characteristics of their microenvironment. The ability of the prepared silicas to distinguish organic solvents colorimetrically was demonstrated for twelve different solvents. We showed that stilbazolium dyes exhibit solvatochromic and fluorosolvatochromic behaviours when covalently bound to the silica surface. The prepared compounds and silicas showed significantly different colour responses to UV-grade methanol and ethanol, which could be applied for routine analyses of the quality of colourless spirits. Moreover, we found peculiar fluorescence in the presence of aromatic compounds, similar to the response to acids. We observed a colour response toward moisture, and we demonstrated the analysis in a flow-through arrangement. We believe that the following research on fluorosolvatochromic silicas will lead to the development of cheap colorimetric sensors and sensor arrays for a wide range of applications, including routine detection of chemicals by a nonprofessional eye.

## Figures and Tables

**Table 1:** Overview of the wavelengths of the maxima of the absorption and fluorescence emission (λ_ex_ = 366 nm) bands of stilbazolium dyes **5** measured in different solvents.

	Stilbazolium Dye 5a					Stilbazolium Dye 5b				
Solvent ^a^	λ_max,1_ [nm]	λ_max,2_ [nm]	λ_max,3_ [nm]	λ_em_ [nm]	Stokes shift [nm]	λ_max,1_ [nm]	λ_max,2_ [nm]	λ_max,3_ [nm]	λ_em_ [nm]	Stokes Shift [nm]
Hydrochloric acid	378	-	-	496	130	391	-	-	540	174
Acetic acid	387	-	-	490	124	410	-	-	546	180
Water	375	-	-	510	144	390	-	-	560	194
Methanol	393	-	-	506	140	417	-	-	562	196
Ethanol	402	516	-	502	136	426	592	-	560	194
Isopropyl alcohol	406	552	-	496	130	430	-	-	554	188
Acetonitrile	385	-	-	500	134	408	-	-	554	188
Dimethyl sulfoxide	395	-	581	512	146	416	-	-	580	214
*N*,*N*-Dimethylformamide	391	-	591	508	142	419	607	644	562	196
Acetone	390	-	-	500	134	415	-	-	558	192
Nitrobenzene	- ^b^	-	607	-	-	- ^b^	-	-	-	-
Pyridine	499	577	607	-	-	439	610	656	550	184

^a^ The order of polarity decreases according to the $E_{T}^{N}$ polarity scale [41,42]; ^b^ band overlap due to the strong absorption of nitrobenzene.

## Data Availability

All data used to support the findings of this study are included in the article and Supplementary Materials. Spectra in raw data format are available upon request.

## Supplementary Materials

The supporting information can be downloaded at: https://www.mdpi.com/article/10.3390/molecules29030673/s1, The Electronic Supporting Information contains detailed preparations of all compounds and modified silicas, supporting experiments, selected NMR, UV–Vis, and fluorescence emission spectra, and optical images (PDF). Refs. [43,44,45] are cited in the Supplementary Materials.
