# Supplementary material for: Silicas with Covalently Anchored Fluorosolvatochromic Dyes Suitable for Naked-Eye Detection of Colourless Compounds
Source: Molecules. 2024 Jan 31;29(3):673. doi: 10.3390/molecules29030673 (PMC10856631; doi:10.3390/molecules29030673)
Supplement: Supplementary file 1 [file molecules-29-00673-s001.zip › molecules-2839104-supplementary.pdf]

SUPPLEMENTARY INFORMATION

Silicas with Covalently Anchored Fluorosolvatochromic Dyes Suitable for Naked-Eye Detection of Colourless Compounds

Tereza Navrátilová, Martin Havlík, Ameneh Tatar, Karolína Hricková and Bohumil Dolenský *

Department of Analytical Chemistry, University of Chemistry and Technology, Prague, Technická 5, 166 28 Prague, Czech Republic; navratit@vscht.cz (T.N.); martin.havlik@vscht.cz (M.H.); tatare@vscht.cz (A.T.)

* Correspondence: dolenskb@vscht.cz; Tel.: +420-220-444-110

CONTENTS

S01-S05	1. Preparation of compounds and materials
S01-S02	1.1 Preparation of model compounds **5** for UV-Vis and fluorescence measurement
S02-S04	1.2 Preparation of modified silicas **10**
S04-S04	1.3 Preparation of model compounds **11** bearing silyl group
S04-S05	1.4 Preparation of model compounds **12a** and **13a**
S06-S28	2. NMR spectra
S06-S08	^{1}H NMR, $^{13}C\{^{1}H\}$ NMR and 2D NMR spectra of pyridine salt **2**
S08-S10	^{1}H NMR, $^{13}C\{^{1}H\}$ NMR and 2D NMR spectra of stilbazolium salt **4a**
S11-S13	^{1}H NMR, $^{13}C\{^{1}H\}$ NMR and 2D NMR spectra of stilbazolium salt **4b**
S13-S15	^{1}H NMR, $^{13}C\{^{1}H\}$ NMR and 2D NMR spectra of stilbazolium dye **5a**
S16-S18	^{1}H NMR, $^{13}C\{^{1}H\}$ NMR and 2D NMR spectra of stilbazolium dye **5b**
S18-S20	^{1}H NMR, $^{13}C\{^{1}H\}$ NMR and 2D NMR spectra of stilbazole **7a**
S21-S23	^{1}H NMR, $^{13}C\{^{1}H\}$ NMR and 2D NMR spectra of stilbazole **7b**
S23-S25	^{1}H NMR, $^{13}C\{^{1}H\}$ NMR and 2D NMR spectra of stilbazolium salt **12a**
S26-S28	^{1}H NMR, $^{13}C\{^{1}H\}$ NMR and 2D NMR spectra of stilbazolium dye **13a**
S29-S45	3. Fluorosolvatochromic evaluation of model compounds **5**
S29-S29	3.1 Preparation of samples for UV-Vis and fluorescence spectra measurement
S30-S39	3.2 UV-Vis spectra of stilbazolium dyes **5**
S40-S43	3.3 Fluorescence emission spectra of stilbazolium dyes **5**
S44-S44	3.4 Colorimetric evaluation of stilbazolium dyes **5**
S45-S45	4. Fluorosolvatochromic evaluation of stilbazolium salt **12a** and stilbazolium dye **13a**
S46-S46	5. Elemental analysis of modified silicas **10**
S47-S47	6. Colorimetric evaluation of modified silicas **10**
S47-S47	7. Supplemental references

1. Preparation of compounds and materials

1.1 Preparation of model compounds 5 for UV-Vis and fluorescence measurement

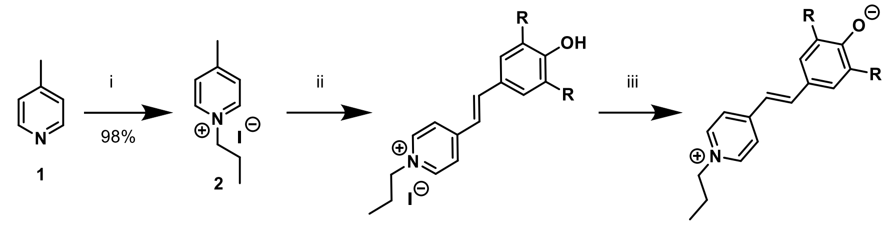

4a: 80%, R = H
4b: 90%, R = OCH_3

5a: 92%, R = H
5b: 86%, R = OCH_3

i) $I(CH_2)_2CH_3$, THF, 70 °C, 4 days
ii) 4-Hydroxybenzaldehyde (***3a***) or 3,5-dimethyl-4-hydroxybenzaldehyde (***3b***) / piperidine, ethanol, 40°C, overnight
ii) Filtration through K_2CO_3, CH_3CN/CH_3Cl (1:1)

Scheme S1 Preparation of model compounds – stilbazolium dyes **5**.

Pyridinium salt **2**. Prepared following the published procedure [S1]. 4-methylpyridine (***1***, 5.0 g, 53.7 mmol) was treated with iodopropane (6.2 ml, 10.8 g, 63.5 mmol) in tetrahydrofuran (40 mL) at 70°C for 4 days. The reaction mixture was cooled to room temperature and diethyl ether (30 mL) was added. The obtained solid was filtered, washed by diethyl ether and dried under vacuum to obtain 13.8 g (98 %) of pyridinium salt **2**. **2:** Known compound [S1]. ^{1}H NMR (500.16 MHz, DMSO-*d6*): δ 8.958 – 8.900 (2H, m, H4), 7.998 (2H, d, *J* = 6.2 Hz, H3), 4.498 (2H, t, *J* = 7.3 Hz, H5), 2.610 (3H, s, H1), 1.910 (2H, h, *J* = 7.4 Hz, H6), 0.863 (3H, t, *J* = 7.4 Hz, H7). ^{13}C{^{1}H} NMR (125.77 MHz, DMSO-*d6*): δ 158.77 (C2), 143.66 (C4), 128.31 (C3), 61.21 (C5), 23.94 (C6), 21.35 (C1), 10.15 (C7). HRMS (ESI$^+$, MeOH): for $C_9H_{14}N$ calcd. $[M]^+$ 136.11317, found 136.11212.

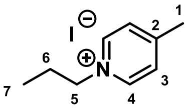

Stilbazolium salt **4a**. Pyridinium salt **2** (2.6 g, 9.9 mmol) was treated with 4-hydroxybenzaldehyde (***3a***, 1.8 g, 14.7 mmol) and piperidine (0.5 mL) in ethanol (30 mL) at reflux temperature overnight. The obtained solid was filtered, washed by ethanol then diethyl ether and dried under vacuum to obtain 2.9 g (80 %) of stilbazolium salt **4a**. **4a:** New compound. ^{1}H NMR (500.16 MHz, DMSO-*d6*): δ 8.890 (2H, m, *J* = 7.0 Hz cov., H9); 7.999 (2H, m, *J* = 7.0 Hz cov., H8); 7.882 (1H, d, *J* = 16.0 Hz, H5); 7.512 (2H, m, *J* = 8.8 Hz cov., H3); 7.063 (1H, d, *J* = 16.0 Hz, H6); 6.671 (2H, m, *J* = 8.8 Hz cov., H2); 5.471 (1H, br s, OH); 4.350 (2H, t, *J* = 7.2 Hz, H10); 1.889 (2H, h, *J* = 7.3 Hz, H11); 0.880 (3H, t, *J* = 7.4 Hz, H12). ^{13}C{^{1}H} NMR (125.77 MHz, DMSO-*d6*): δ 166.43 (C1), 153.63 (C7), 143.19 (C9), 142.49 (C5), 131.00 (C3), 122.84 (C4), 121.94 (C8), 117.60 (C2), 116.20 (C6), 60.27 (C10), 23.85 (C11), 10.25 (C12). HRMS (ESI$^+$, MeOH): for $C_{16}H_{17}NO$ calcd. $[M+H]^+$ 240.13939, found 240.13825; calcd. $[M+Na]^+$ 262.12024, found 262.12007.

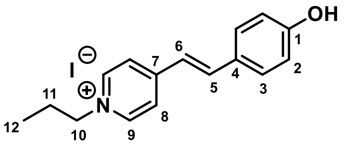

Stilbazolium salt **4b**. Pyridinium salt **2** (2.6 g, 9.9 mmol) was treated with 4-hydroxy-3,5-dimethoxybenzaldehyde (***3b***, 2.7 g, 14.8 mmol) and piperidine (0.5 mL) in ethanol (30 mL) at reflux temperature overnight. The obtained solid was filtered, washed by ethanol then diethyl ether and dried under vacuum to obtain 3.8 g (90 %) of stilbazolium salt **4b**. **4b:** New compound. ^{1}H NMR (500.16 MHz, DMSO-*d6*): δ 9.156 (1H, s, OH), 8.861 (2H, d, *J* = 6.7 Hz, H9), 8.120 (2H, d, *J* = 6.7 Hz, H8), 7.940 (1H, d, *J* = 16.2 Hz, H5), 7.372 (1H, d, *J* = 16.2 Hz, H6), 7.064 (2H, s, H3), 4.421 (2H, t, *J* = 7.3 Hz, H10), 3.842 (6H, s, H13), 1.925 (2H, h, *J* = 7.3 Hz, H11), 0.896 (3H, t, *J* = 7.4 Hz, H12). ^{13}C{^{1}H} NMR (125.77 MHz, DMSO-*d6*): δ 153.33 (C7), 148.21 cov. (C1, C2), 143.93 (C9), 142.01 (C5), 125.42 (C4), 122.94 (C8), 120.06 (C6), 106.22 (C3), 60.79 (C10), 56.08 (C13), 23.86 (C11), 10.27 (C12). HRMS (ESI$^+$, MeOH): for $C_{18}H_{21}NO_3$ calcd. $[M+H]^+$ 300.15942, found 300.15967; calcd. $[M+Na]^+$ 322.14136, found 322.14125.

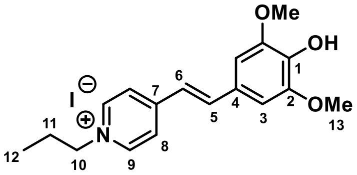

Stilbazolium dye **5a**. Stilbazolium salt **4a** (1.0 g, 2.7 mmol) was treated with potassium carbonate (500 mg, 3.6 mmol) in the mixture of acetonitrile and chloroform (1:1, 10 mL) at room temperature for 1 hour. The reaction mixture was filtered trough a short silica column and evaporated to dried under vacuum to obtain 0.6 g (92 %) of stilbazolium dye **5a**. **5a:** Known compound [S2-S3]. ^{1}H NMR (500.16 MHz, DMSO-d_6): δ 8.282 (2H, d, *J* = 7.0 Hz, H9), 7.706 (1H, d, *J* = 15.2 Hz, H5), 7.586 (2H, br d, *J* = 7.0 Hz, H8), 7.325 (2H, br d, *J* = 8.7 Hz, H3), 6.564 (1H, d, *J* = 15.2 Hz, H6), 6.187 (2H, d, *J* = 8.7 Hz, H2), 4.150 (2H, t, *J* = 7.2 Hz, H10), 1.817 (2H, h, *J* = 7.3 Hz, H11), 0.862 (3H, t, *J* = 7.4 Hz, H12). ^{13}C{^{1}H} NMR (125.77 MHz, DMSO-d_6): δ 177.24 (C1), 153.24 (C7), 143.88 (C5), 141.41 (C9), 132.73 (C3), 120.81 (C2), 119.09 (C8), 117.10 (C4), 109.04 (C6), 59.04 (C10), 23.72 (C11), 10.30 (C12). HRMS (ESI$^+$, MeOH): for $C_{16}H_{17}NO$ calcd. [M+H]$^+$ 240.13829, found 240.13828; calcd. [M+Na]$^+$ 262.12024, found 262.12003.

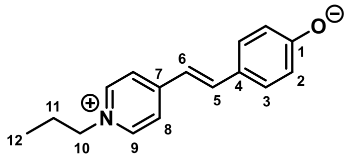

Stilbazolium dye **5b**. Stilbazolium salt **4b** (1.0 g, 2.3 mmol) was treated with potassium carbonate (500 mg, 3.6 mmol) in the mixture of acetonitrile and chloroform (1:1, 10 mL) at room temperature for 1 hour. The reaction mixture was filtered trough a short silica column and evaporated to dried under vacuum to obtain 0.6 g (86 %) of stilbazolium dye **5b**. **5b:** New compound. ^{1}H NMR (500.16 MHz, DMSO-d_6): δ 8.687 (2H, br d, *J* = 6.6 Hz, H9), 7.957 (2H, br d, *J* = 6.6 Hz, H8), 7.875 (1H, d, *J* = 15.9 Hz, H5), 7.190 (1H, br d, *J* = 15.9 Hz, H6), 7.012 (2H, s, H3), 4.337 (2H, t, *J* = 7.2 Hz, H10), 3.804 (6H, s, H13), 1.894 (2H, h, *J* = 7.3 Hz, H11), 0.888 (3H, t, *J* = 7.4 Hz, H12). ^{13}C{^{1}H} NMR (125.77 MHz, DMSO-d_6): δ 152.83 (C7), 148.73 (C1), 143.16 (C9), 142.14 (C5), 123.41 (C4), 121.81 (C8), 117.59 (C6), 106.79 (C3), 60.27 (C10), 55.94 (C13), 23.81 (C11), 10.28 (C12). HRMS (ESI$^+$, MeOH): for $C_{18}H_{21}NO_3$ calcd. [M+H]$^+$ 300.15942, found 300.15976; calcd. [M+Na]$^+$ 322.14136, found 322.14120.

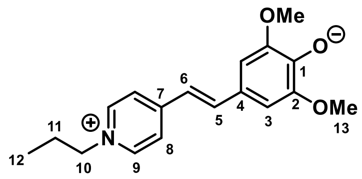

1.2 Preparation of modified silicas 10

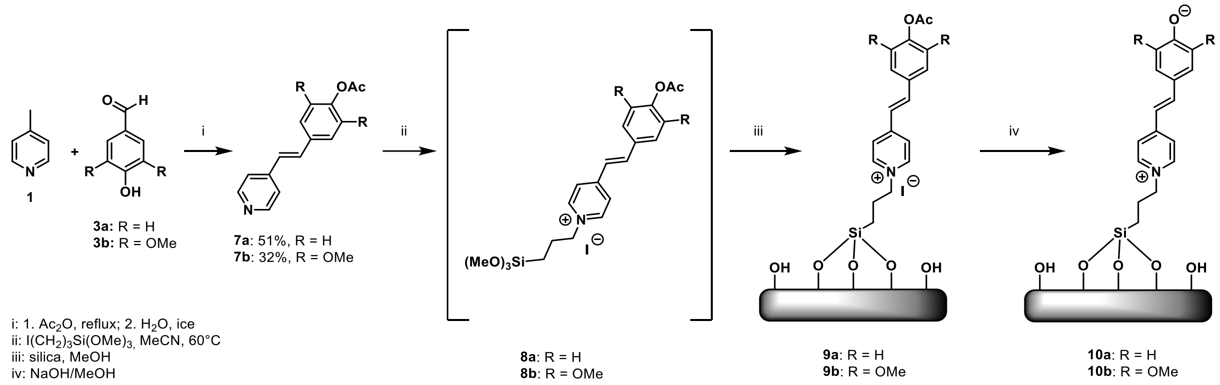

Scheme S2 Preparation of modified silicas **10**.

Stilbazole 7a: 4-Hydroxybenzaldehyde (***3a***, 15.2 g, 130 mmol) and 4-methylpyridine (***1***, 9.48 g, 100 mmol) were placed in a round bottom flask and dissolved in acetic anhydride (20 mL). The solution was stirred at 170°C for 24 hours. The progress of the reaction was monitored by TLC ($CHCl_3$:MeOH, 49:1 v/v). The reaction mixture was then poured onto ice water. After decantation with water, precipitate was recrystallized from acetonitrile to give 12.5 g (51%) of stilbazole **7a** as a pale yellow powder. **7a:** Known compound [S4]. ^{1}H NMR (500 MHz, $CDCl_3$): δ 8.547 (2H, dd, *J* = 4.5, 1.6 Hz, H9), 7.696 (2H, dd, *J* = 6.6, 2.0 Hz, H3), 7.550 (2H, dd, *J* = 4.5, 1.6 Hz, H8), 7.549 (1H, d, *J* = 16.5 Hz, H5), 7.227 (1H, d, *J* = 16.5 Hz, H6), 7.181 (2H, dd, *J* = 6.6, 2.0 Hz, H2), 2.280 (3H, s, H11). ^{13}C{^{1}H} NMR (126 MHz, $CDCl_3$): δ 169.20 (C10), 150.66 (C1), 150.09 (C9), 144.23 (C7), 133.90 (C4), 132.08 (C5), 128.16 (C3), 126.12 (C6), 122.32 (C2), 120.90 (C8), 20.93 (C11). ^{15}N NMR (50.68 MHz, $CDCl_3$, from ^{1}H-^{15}N HMBC): δ 309.394. HRMS (ESI$^+$, MeOH): for $C_{15}H_{13}NO_2$ calcd. [M+H]$^+$ 240.10191, found 240.10207.

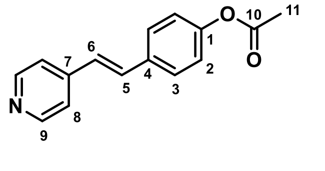

Stilbazole 7b: 4-Hydroxy-3,5-dimethoxybenzaldehyde (***3b***, 4.38 g, 24.0 mmol) and 4-methylpyridine (***1***, 2.24 g, 24.0 mmol) were placed in a round bottom flask and dissolved in acetic anhydride (20 mL). The solution was stirred at 150°C for 72 hours. The progress of the reaction was monitored by TLC ($CHCl_3$:MeOH, 49:1 v/v). The reaction mixture was then poured onto ice water. Following extraction (DCM/H_2O) yielded after evaporation under reduced pressure 2.30 g (32%) of stilbazole **7b** as a yellow-orange crystallic compound. **7b:** New compound. ^{1}H NMR (500 MHz, $CDCl_3$): δ 8.586 (2H, d, J = 5.7 Hz, H9), 7.391 (2H, d, J = 5.7 Hz, H8), 7.228 (1H, d, J = 16.3 Hz, H5), 6.931 (1H, d, J = 16.3 Hz, H6), 6.756 (2H, s, H3), 3.848 (6H, s, H12), 2.333 (3H, s, H11). ^{13}C{^{1}H} NMR (126 MHz, $CDCl_3$): δ 168.69 (C10), 152.47 (C2), 149.12 (C9), 145.43 (C7), 144.29 (C1), 134.44 (C4), 133.70 (C5), 125.95 (C6), 121.22 (C8), 103.84 (C3), 56.22 (C12), 20.47 (C11). ^{15}N NMR (50.68 MHz, $CDCl_3$, from ^{1}H-^{15}N HMBC): δ 292.232. HRMS (ESI$^+$, MeOH): for $C_{17}H_{17}NO_4$ calcd. $[M+H]^+$ 300.12303, found 300.12314.

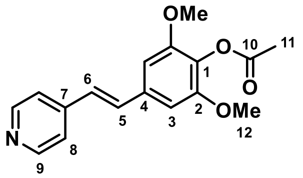

Modified silica 9a: Stilbazole **7a** (138 mg, 0.577 mmol) and (3-iodopropyl)trimethoxysilane (395 mg; 1.36 mmol) were dissolved in acetonitrile (5 mL). The solution was heated to 60 °C for 3 days. The progress of the reaction was monitored by TLC analysis until the consumption of the starting stilbazole **7a**. The reaction was quenched by evaporating to dryness under reduced pressure. The crude product of stilbazolium salt **8a** (453 mg) was obtained in the form of a yellow crystalline substance. Due to its difficult purification and low stability, stilbazolium salt **8a** was used as a crude product for the next step without further purification. The crude product of **8a** (39.9 mg) was dissolved in 50 mL of a mixture of DCM:MeOH, 19:5 (v/v) and the resulting solution was mixed with 10.0 g of chromatography grade silica. The suspension was left to stand for 12 h with occasional shaking and then evaporated to dryness under reduced pressure to give modified silica **9a**. The prepared material did not show any significant color changes when it was washed on the frit with different solvents. We observed no leakage of the dye when washing the material, even when using solvents such as water, MeOH and DMSO.

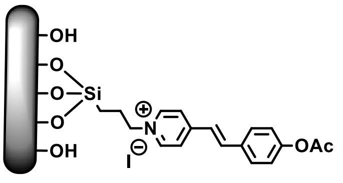

Modified silica 9b: Stilbazole **7b** (173 mg, 0.576 mmol) and (3-iodopropyl)trimethoxysilane (373 mg; 1.29 mmol) were dissolved in acetonitrile (5 mL). The solution was heated to 60 °C for 3 days. The progress of the reaction was monitored by TLC analysis until the consumption of the starting stilbazole **7b**. The reaction was quenched by evaporating to dryness under reduced pressure. The crude product of stilbazolium salt **8b** (425 mg) was obtained in the form of a honey-like viscous liquid. Due to its difficult purification and low stability, stilbazolium salt **8b** was used as a crude product for the next step without further purification. The crude product of **8b** (35.8 mg) was dissolved in 50 mL of a mixture of DCM:MeOH, 19:5 (v/v) and the resulting solution was mixed with 10.0 g of chromatography grade silica. The suspension was left to stand for 12 h with occasional shaking and then evaporated to dryness under reduced pressure to give modified silica **9b**. The prepared material did not show any significant color changes when it was washed on the frit with different solvents. We observed no leakage of the dye when washing the material, even when using solvents such as water, MeOH and DMSO.

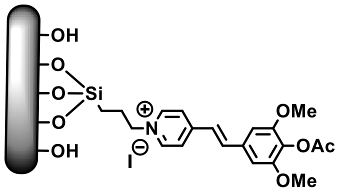

Fluorosolvatochromic silica 10a: The prepared modified silica **9a** was washed with 100 mL of 0.5M solution of NaOH in MeOH to give fluorosolvatochromic silica **10a**. The solid was filtered off on a frit and washed with 250 mL of MeOH to neutral pH. The resulting material was then evaporated to dryness under reduced pressure to yield 12.5 g of orange-red silica material which showed fluorosolvatochromic response to solvents with different polarity. We observed no leakage of the dye when washing the material, even when using solvents such as water, MeOH and DMSO.

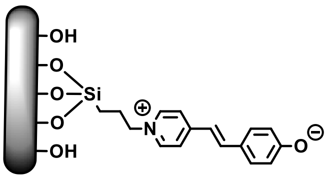

Fluorosolvatochromic silica 10b: The prepared modified silica **9b** was washed with 100 mL of 0.5M solution of NaOH in MeOH to give fluorosolvatochromic silica **10b**. The solid was filtered off on a frit and washed with 250 mL of MeOH to neutral pH. The resulting material was then evaporated to dryness under reduced pressure to yield 11.2 g of dark purple silica material which showed fluorosolvatochromic response to solvents with different polarity. We observed no leakage of the dye when washing the material, even when using solvents such as water, MeOH and DMSO.

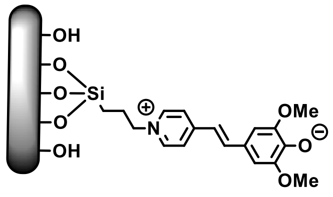

1.3 Preparation of model compounds 11 bearing silyl group

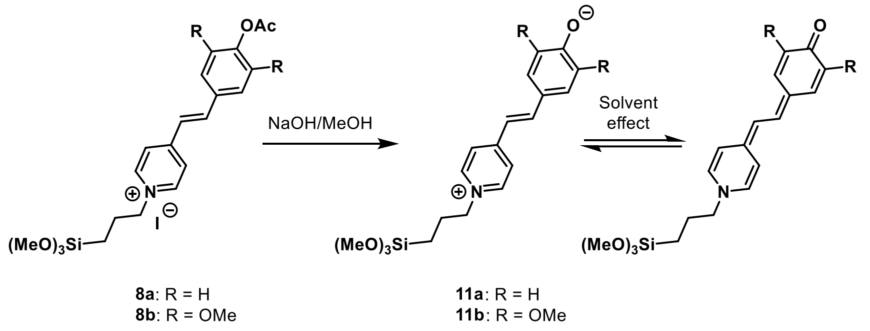

Scheme S3 Preparation of model compounds bearing silyl group – stilbazolium dyes **11**.

Stilbazolium dye 11a: The crude product for the preparation of stilbazolium salt **8a** (22.9 mg) was weighted into an 5 mL Eppendorf tube and dissolved in 2 mL of MeOH. Into this solution, 1 mL of 1M solution of NaOH in MeOH was added. Resulting solution was stirred for 10 minutes. Thus prepared stilbene **11a** was tested for solvatochromic behavior as a prepared solution without further purification due to its difficult purification and low stability.

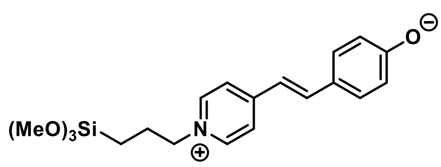

Stilbazolium dye 11b: The crude product for the preparation of stilbazolium salt **8b** (20.2 mg) was weighted into an 5 mL Eppendorf tube and dissolved in 2 mL of MeOH. Into this solution, 1 mL of 1M solution of NaOH in MeOH was added. Resulting solution was stirred for 10 minutes. Thus prepared stilbene **11b** was tested for solvatochromic behavior as a prepared solution without further purification due to its difficult purification and low stability.

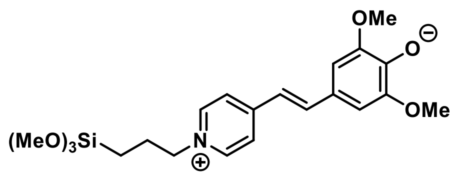

1.4 Preparation of model compounds 12a and 13a

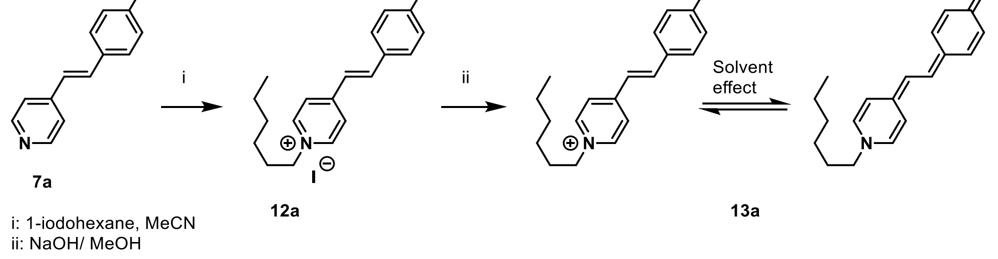

Scheme S4 Preparation of model compounds for deacetylation – stilbazolium salt **12a** and stilbazolium dye **13a**.

Stilbazolium salt 12a: Stilbazole **7a** (373 mg, 1.56 mmol) was dissolved in acetonitrile (50 mL). 1-Iodohexane (1.68 g, 7.91 mmol) was added to the solution. The solution was stirred at 80 °C overnight. The reaction was terminated by evaporating the reaction mixture to dryness. Column chromatography on silica gel (mobile phase: DCM:MeOH, 90:10 v/v) yielded 400 mg of stilbazolium dye **12a** (57%) as a bright yellow crystalline compound. **12a:** New compound. ^{1}H NMR (500 MHz, DMSO-d_6): δ 9.982 (2H, d, *J* = 7.0 Hz, H9), 8.256 (2H, d, *J* = 7.0 Hz, H8), 8.053 (1H, d, *J* = 16.3 Hz, H5), 7.809 (2H, m, *J* = 8.7 Hz cov., H3), 7.524 (1H, d, *J* = 16.3 Hz, H6), 7.270 (2H, m, *J* = 8.7 Hz cov., H2), 4.508 (2H, br t, *J* = 7.4 Hz, H10), 2.295 (3H, s, H17), 1.905 (2H, dt, *J* = 7.2 Hz cov., H11), 1.344 – 1.206 cov. (6H, m, H12, H13, H14), 1.006 – 0.691 (3H, m, H15). ^{13}C{^{1}H} NMR (126 MHz, DMSO-d_6): δ 169.01 (C16), 152.73 (C7), 151.92 (C1), 144.27 (C9), 139.80 (C5), 132.81 (C4), 129.31 (C3), 123.88 (C8), 123.34 (C6), 122.61 (C2), 59.71 (C10), 30.53 (C13), 30.45 (C11), 25.04 (C12), 21.82 (C14), 20.88 (C17), 13.79 (C15). HRMS (ESI$^+$, MeOH): for $C_{21}H_{26}NO_2$ calcd. [M]$^+$ 324.19581, found 324.19624.

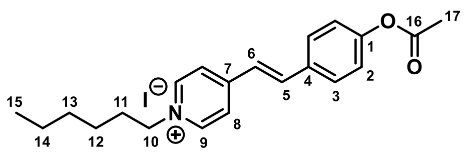

Stilbazolium dye 13a: Stilbazolium dye **12a** (200 mg, 44.3 mmol) was dissolved in 20 mL of a saturated solution of NaOH in MeOH. The solution was stirred for 1 h at room temperature. The reaction mixture was subsequently evaporated to dryness. The residue was suspended in chloroform and the suspension was filtered on a frit. The filtrate was evaporated to dryness to yield 113 mg of stilbazolium dye **13a** (91%) as a purple metallic-glossy crystalline compound. **13a:** Known compound [S3]. ^{1}H NMR (500 MHz, DMSO-d_6): δ 8.192 (2H, d, *J* = 7.1 Hz, H9), 7.668 (1H, d, *J* = 15.1 Hz, H5), 7.493 (2H, br d, *J* = 7.1 Hz, H8), 7.289 (2H, br d, *J* = 8.8 Hz, H3), 6.463 (1H, d, *J* = 15.1 Hz, H6), 6.083 (2H, d, *J* = 8.8 Hz, H2), 4.128 (2H, t, *J* = 7.3 Hz, H10), 1.877 – 1.703 (2H, m, H11), 1.323 – 1.157 cov. (6H, m, H12, H13, H14), 0.905 – 0.815 (3H, m, H15). ^{13}C{^{1}H} NMR (126 MHz, DMSO-d_6): δ 153.10 (C7), 144.08 (C5), 141.07 (C9), 132.95 br (C3), 121.45 cov. (C2, cov. C1), 118.57 (C8), 116.21 (C4), 107.74 (C6), 57.44 (C10), 30.63 (C13), 30.30 (C11), 25.17 (C12), 21.93 (C14), 13.87 (C15). HRMS (ESI$^+$, MeOH): for $C_{19}H_{23}NO$ calcd. [M+H]$^+$ 282.18524, found 282.18562.

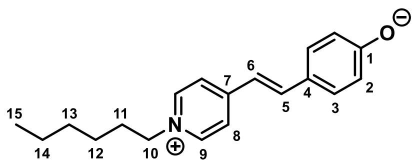

2. NMR spectra

Pyridinium salt 2

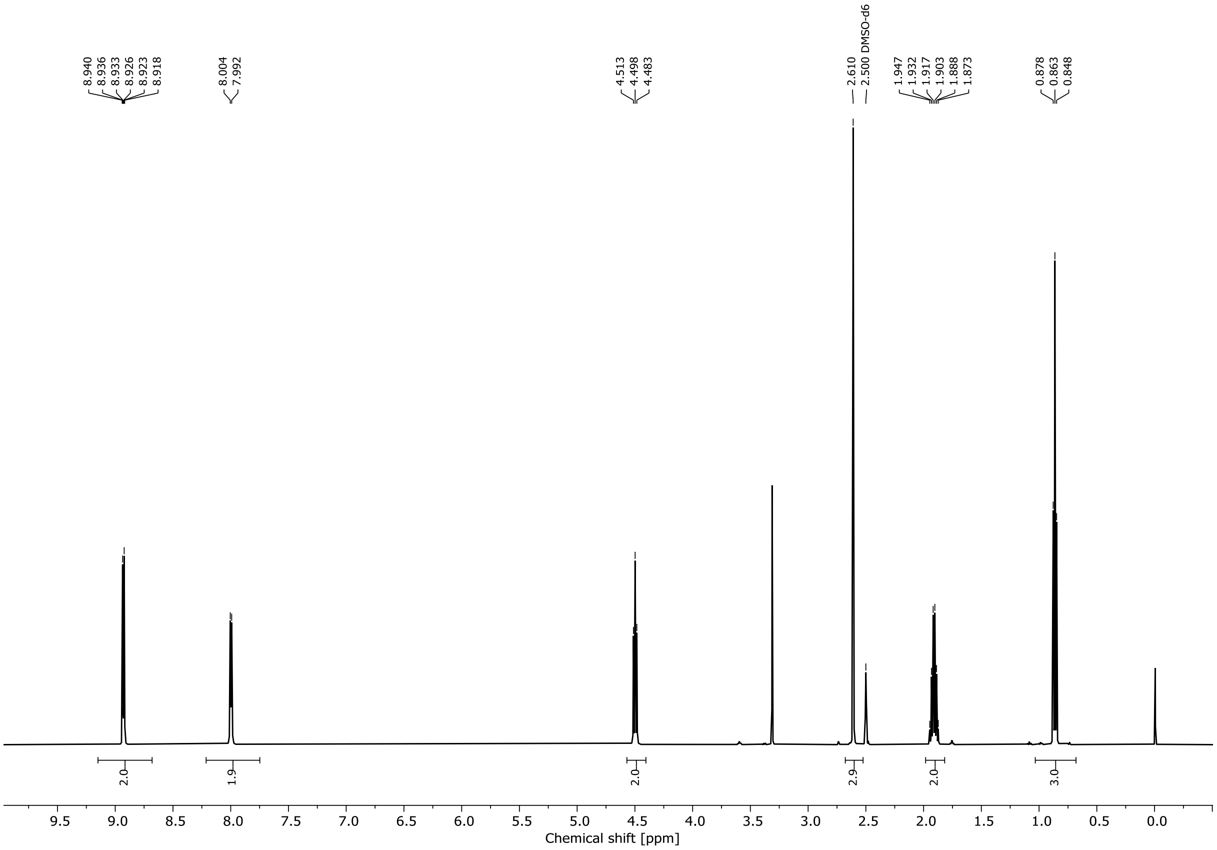

Fig. S1 ^{1}H NMR (500 MHz, DMSO-d_6) spectrum of pyridinium salt **2**.

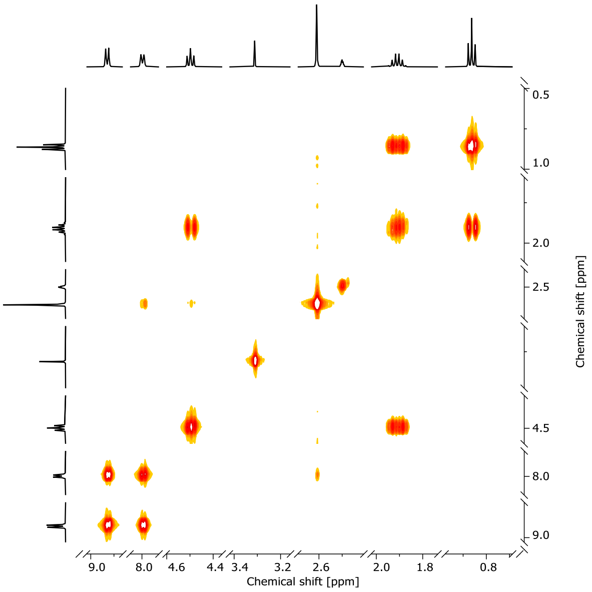

Fig. S2 Section of the ^{1}H-^{1}H COSY spectrum in the signal region of pyridinium salt **2**.

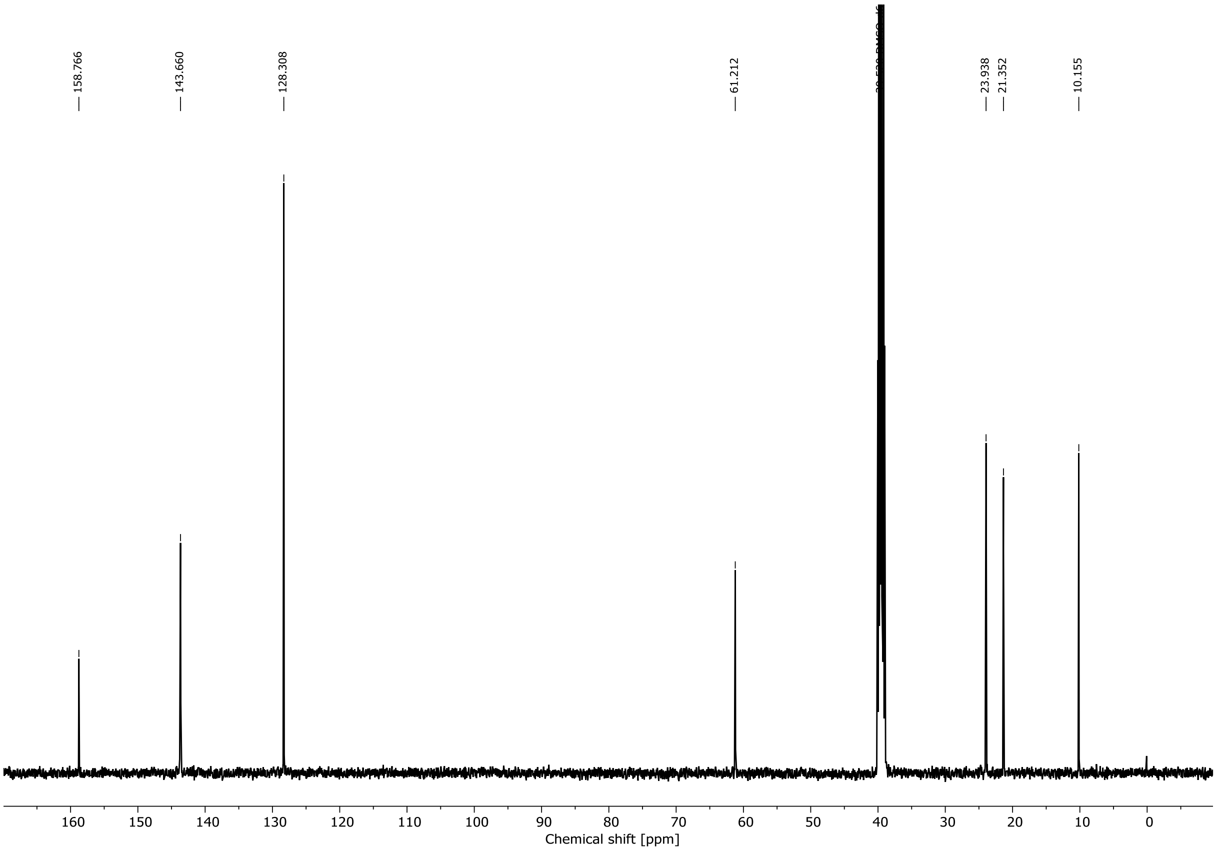

Fig. S3 $^{13}C\{^{1}H\}$ NMR (126 MHz, DMSO-d_6) spectrum of pyridinium salt **2**.

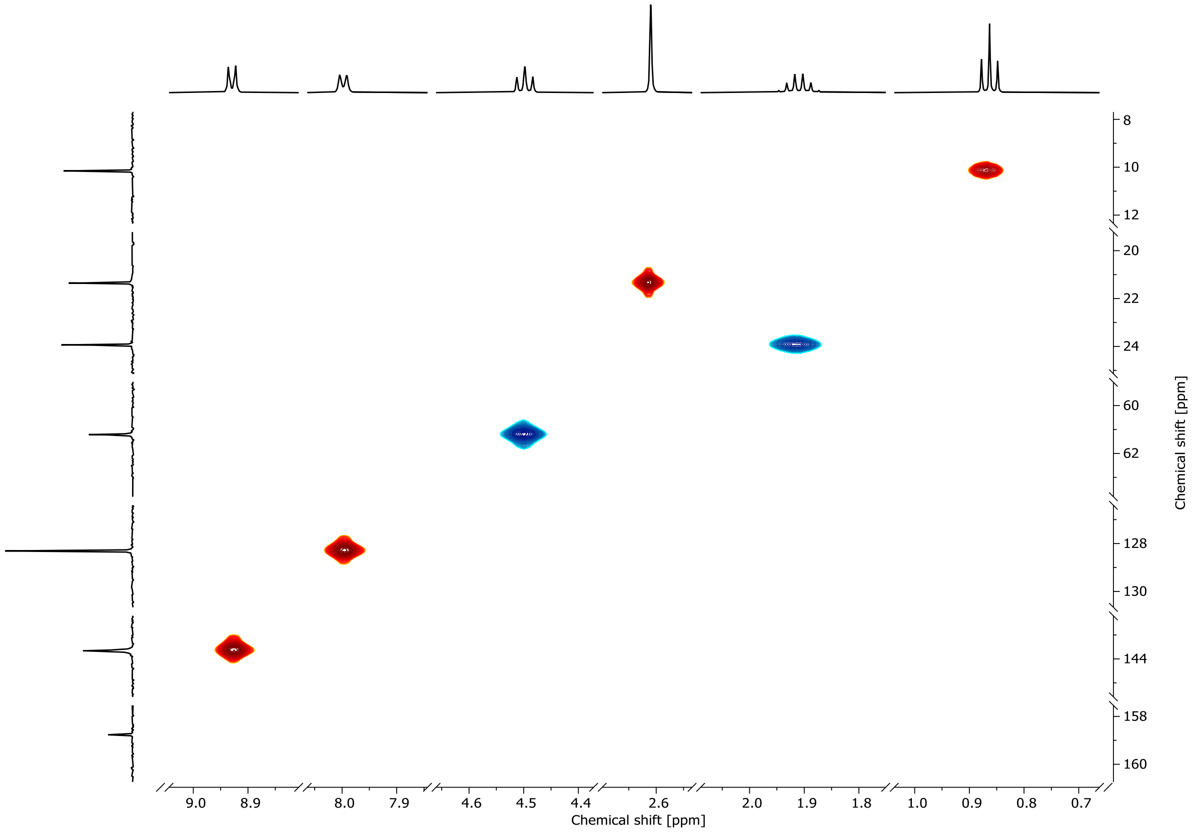

Fig. S4 Section of the ^{1}H-^{13}C HSQC spectrum in the signal region of pyridinium salt **2**.

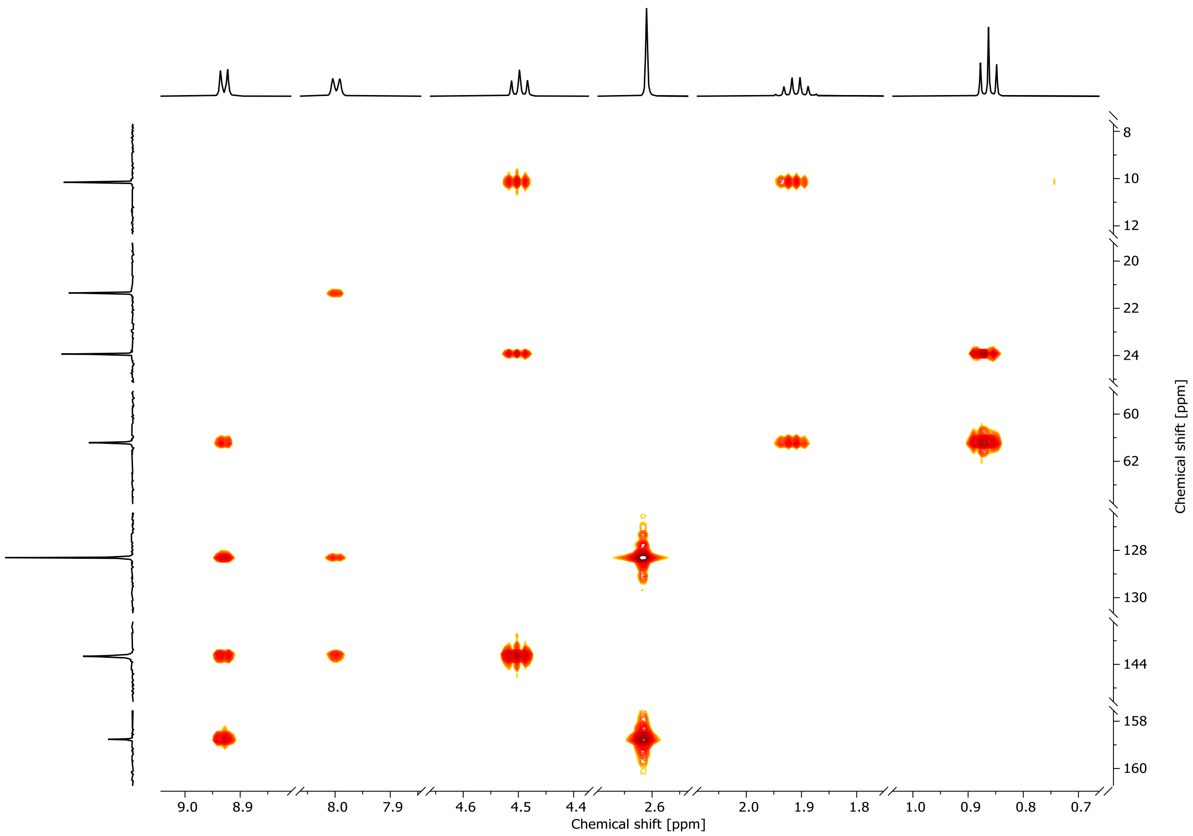

Fig. S5 Section of the ^{1}H-^{13}C HMBC spectrum in the signal region of pyridinium salt **2**.

Stilbazolium salt 4a

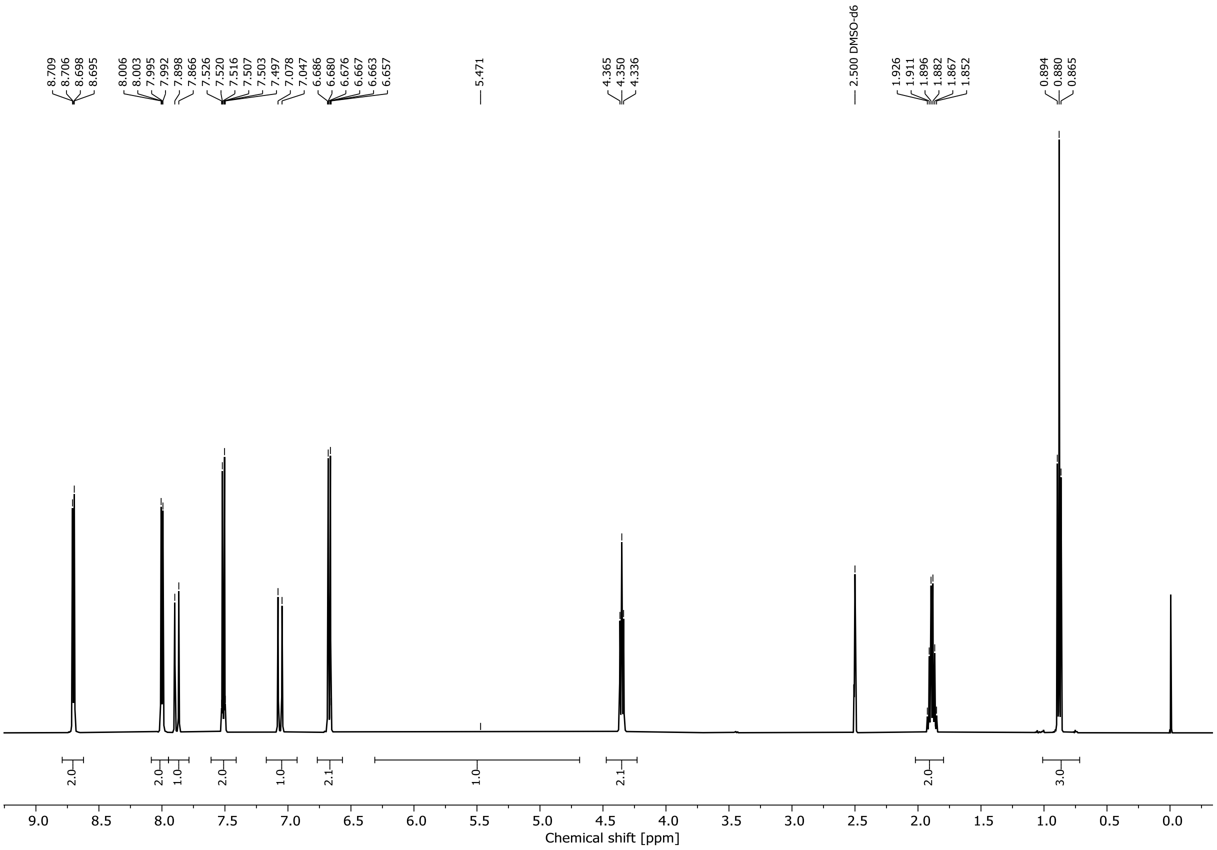

Fig. S6 ^{1}H NMR (500 MHz, DMSO-d_6) spectrum of stilbazolium salt **4a**.

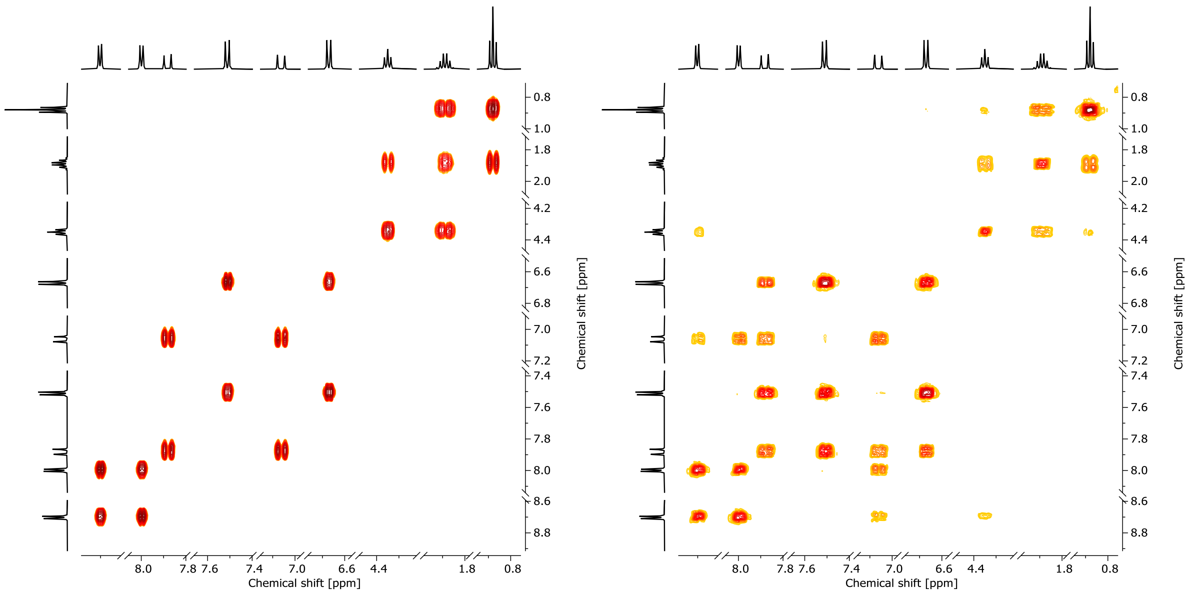

Fig. S7 Section of the ^{1}H-^{1}H COSY spectrum (left) and ^{1}H-^{1}H long range COSY spectrum (right, 450 ms) in the signal region of stilbazolium salt **4a**.

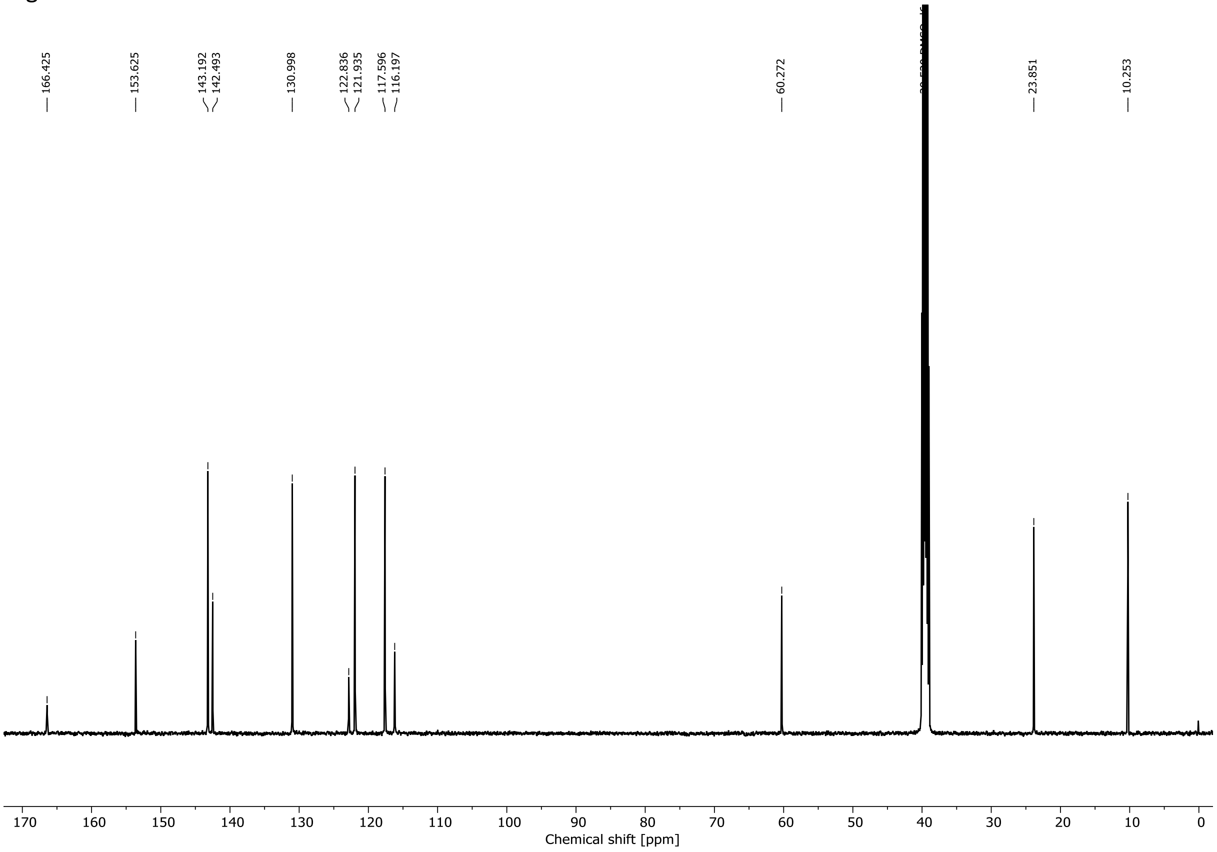

Fig. S8 $^{13}C\{^{1}H\}$ NMR (126 MHz, DMSO-d_6) spectrum of stilbazolium salt **4a**.

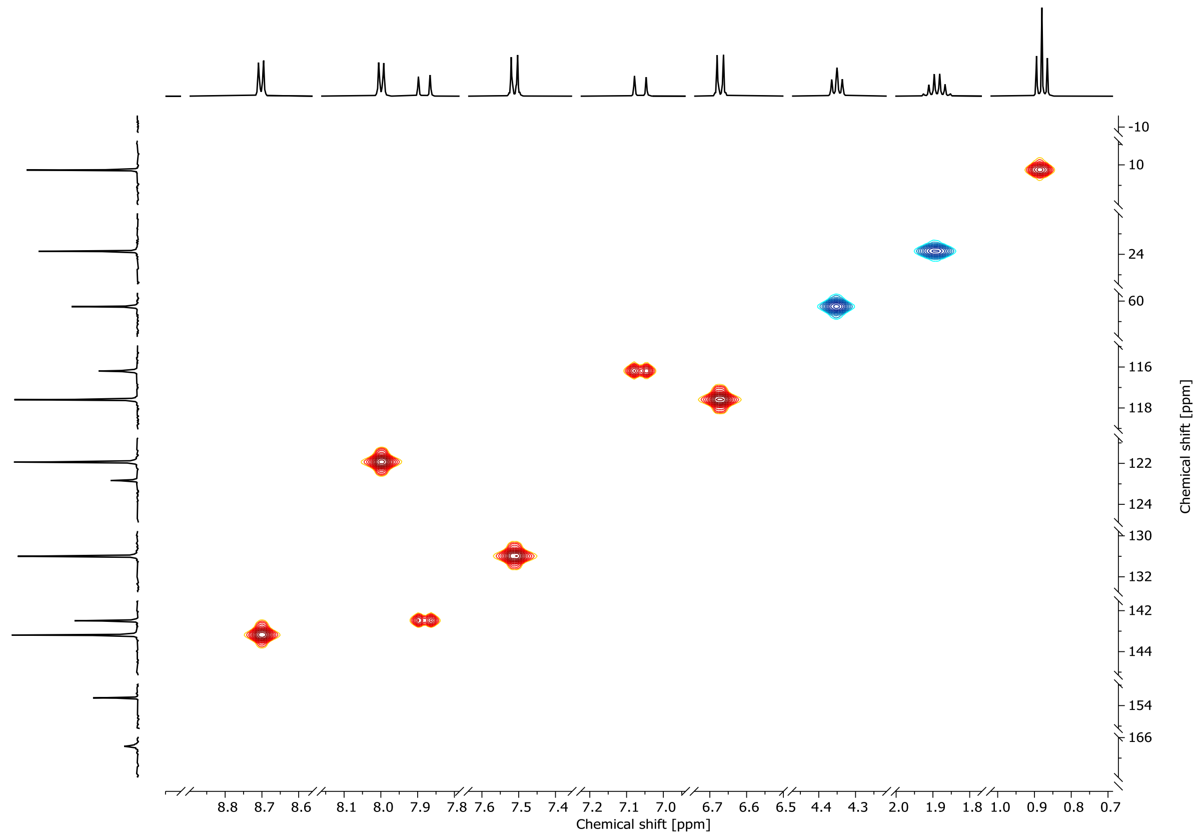

Fig. S9 Section of the ^{1}H-^{13}C HSQC spectrum in the signal region of stilbazolium salt **4a**.

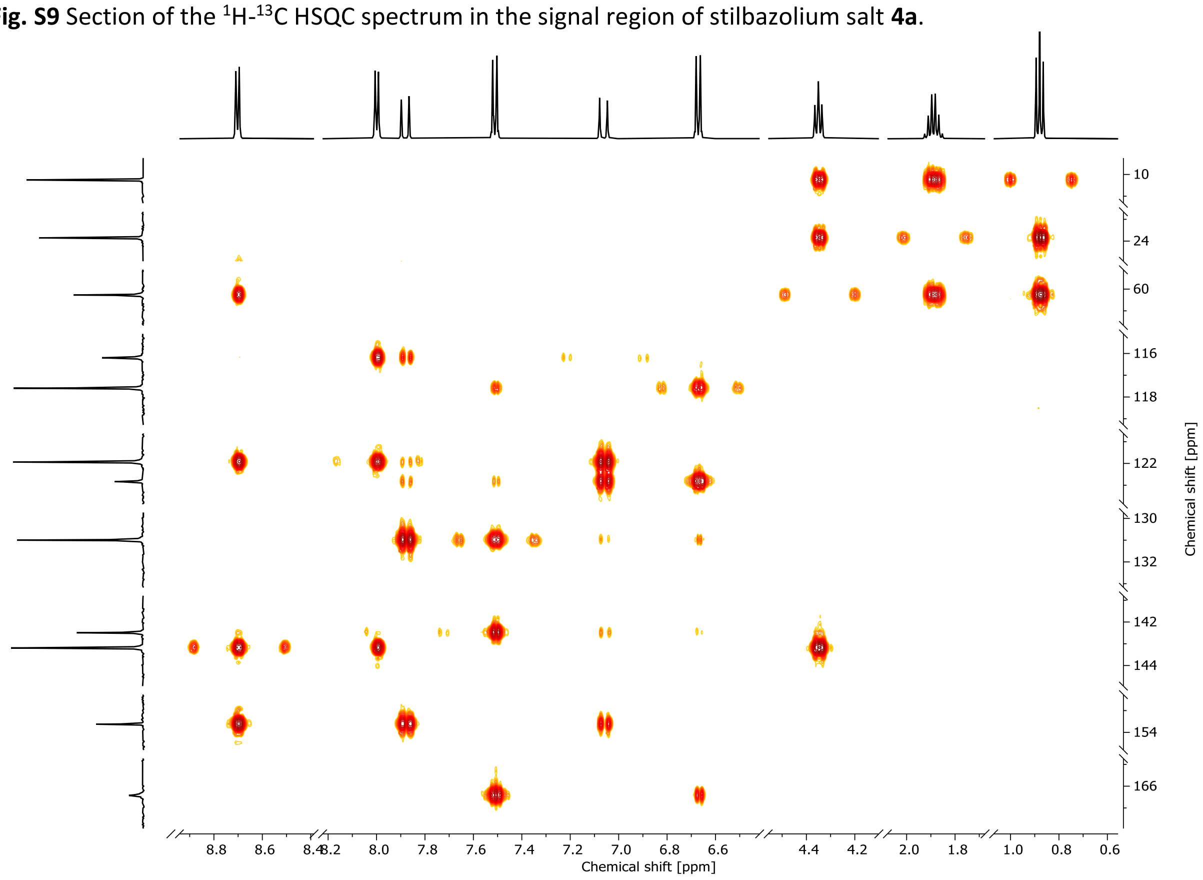

Fig. S10 Section of the ^{1}H-^{13}C HMBC spectrum in the signal region of stilbazolium salt **4a**.

Stilbazolium salt 4b

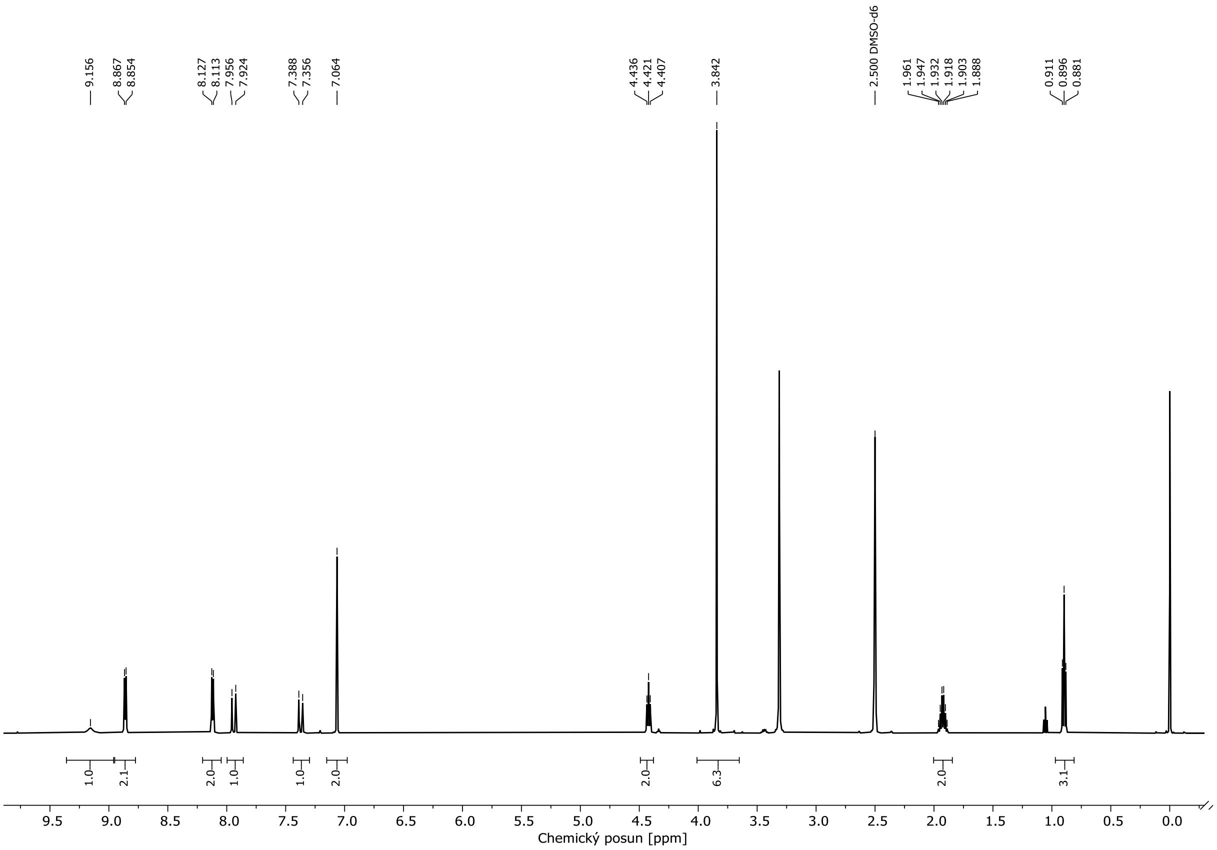

Fig. S11 ^{1}H NMR (500 MHz, DMSO-d_6) spectrum of stilbazolium salt **4b**.

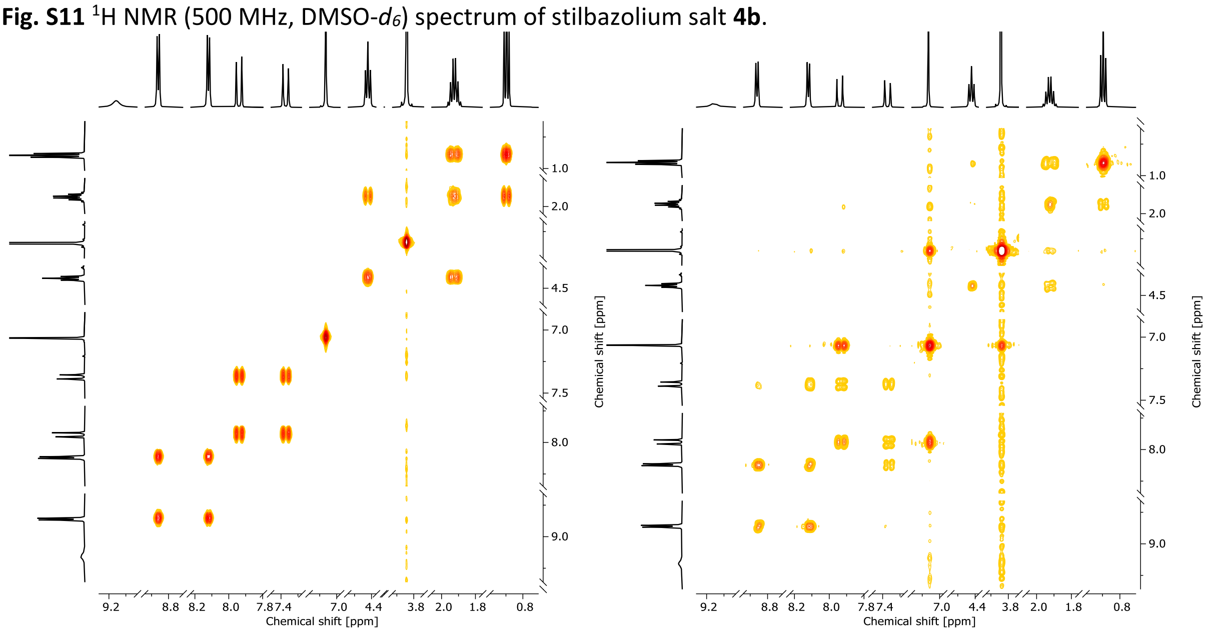

Fig. S12 Section of the ^{1}H-^{1}H COSY spectrum (left) and ^{1}H-^{1}H long range COSY spectrum (right, 450 ms) in the signal region of stilbazolium salt **4b**.

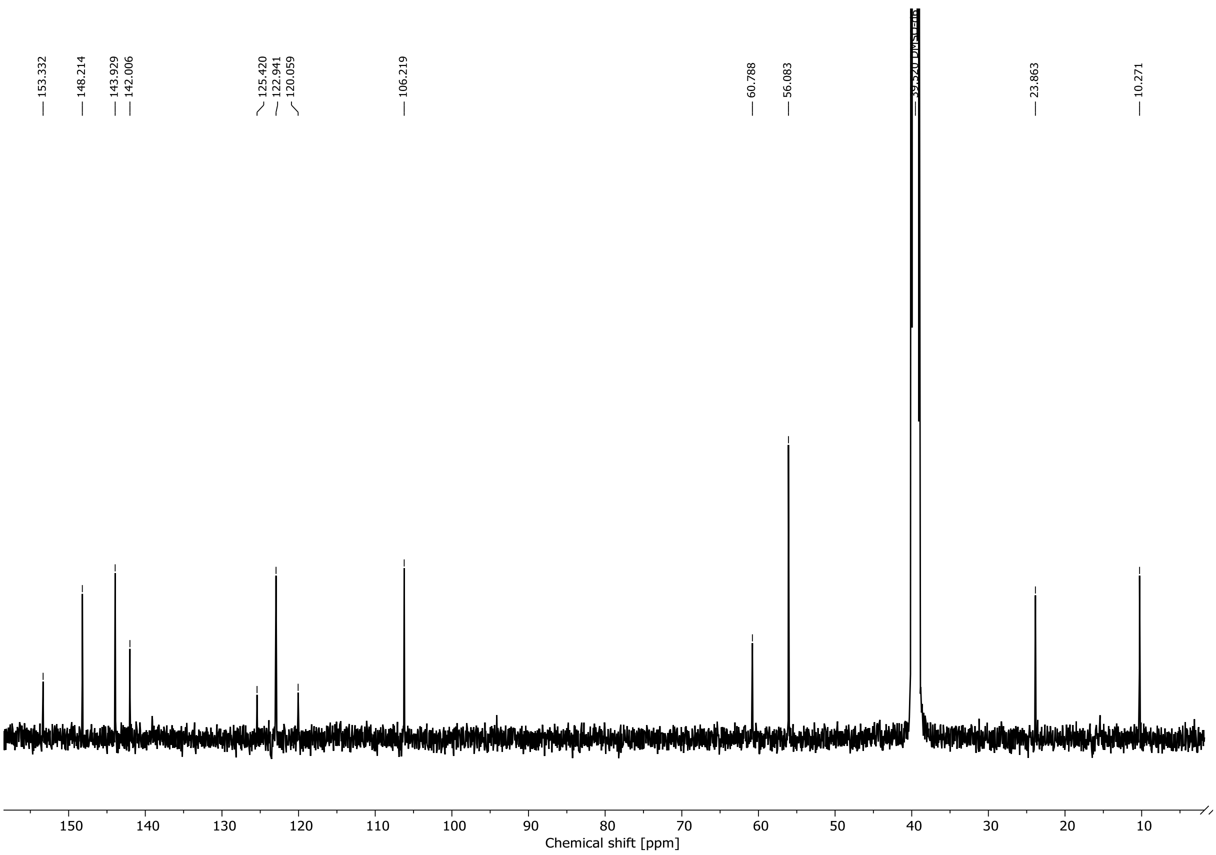

Fig. S13 $^{13}C\{^1H\}$ NMR (126 MHz, DMSO-d_6) spectrum of stilbazolium salt **4b**.

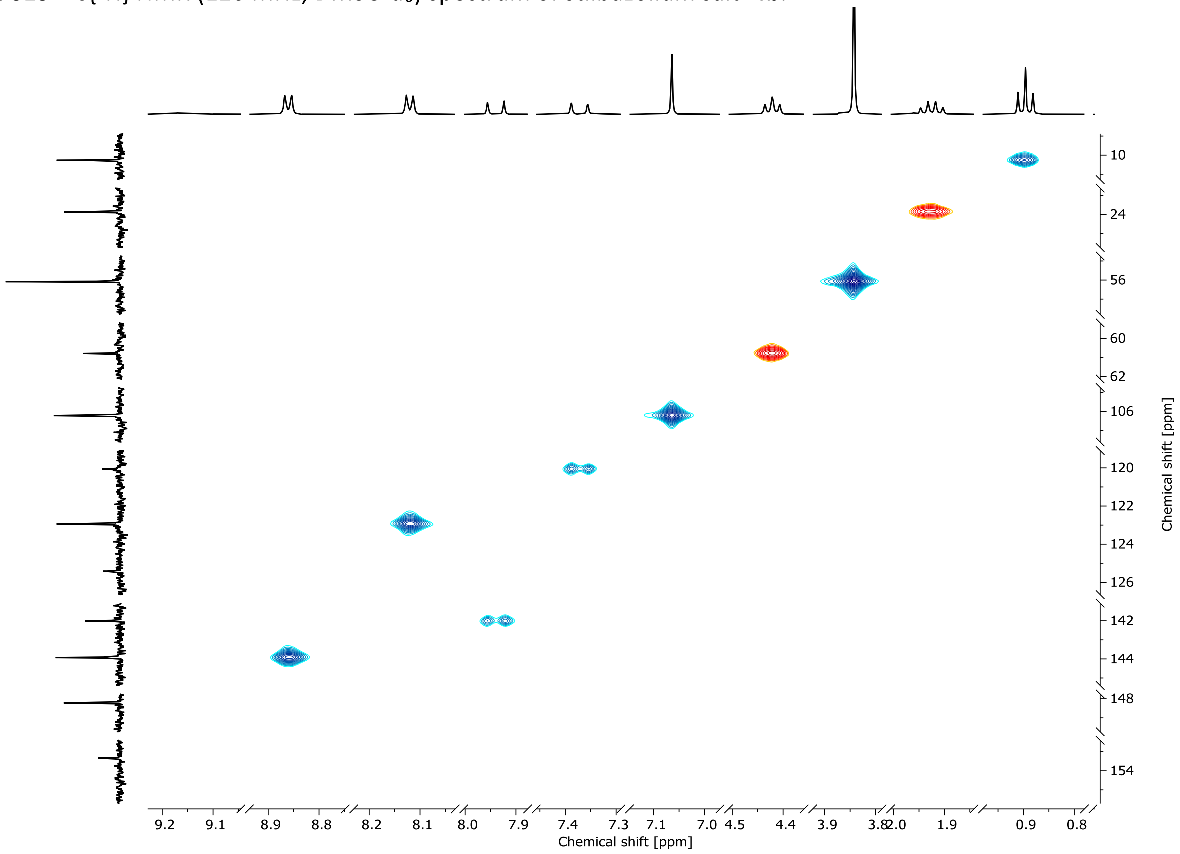

Fig. S14 Section of the ^1H-^{13}C HSQC spectrum in the signal region of stilbazolium salt **4b**.

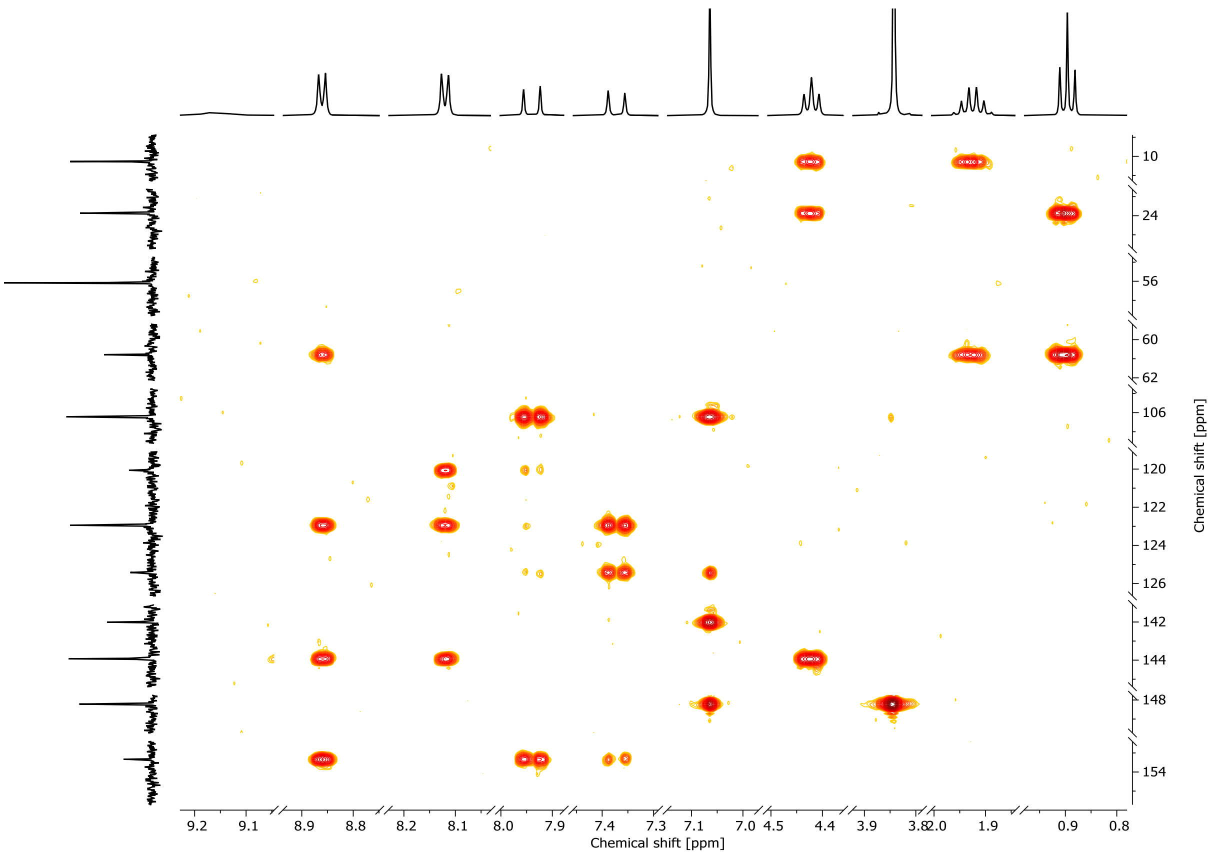

Fig. S15 Section of the ^{1}H-^{13}C HMBC spectrum in the signal region of stilbazolium salt **4b**.

Stilbazolium dye 5a

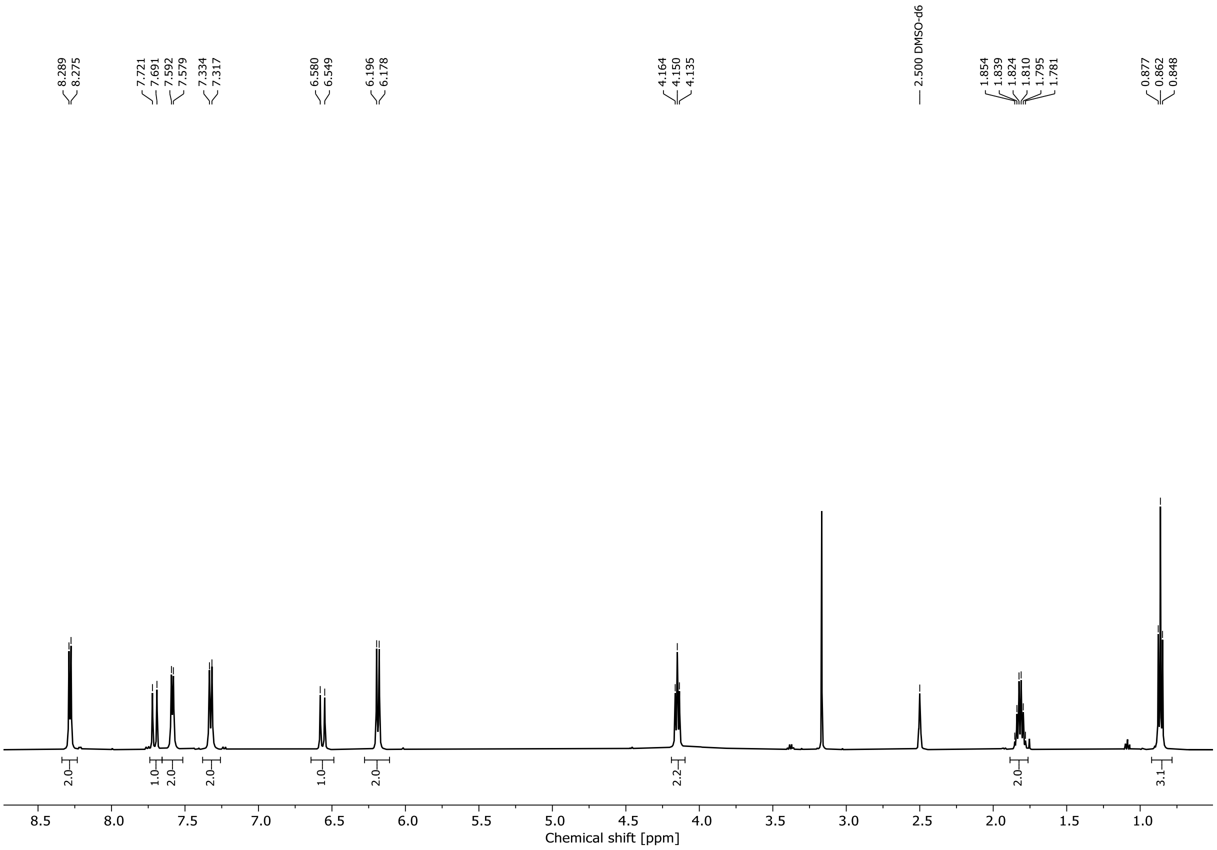

Fig. S16 ^{1}H NMR (500 MHz, DMSO-d_6) spectrum of stilbazolium dye **5a**.

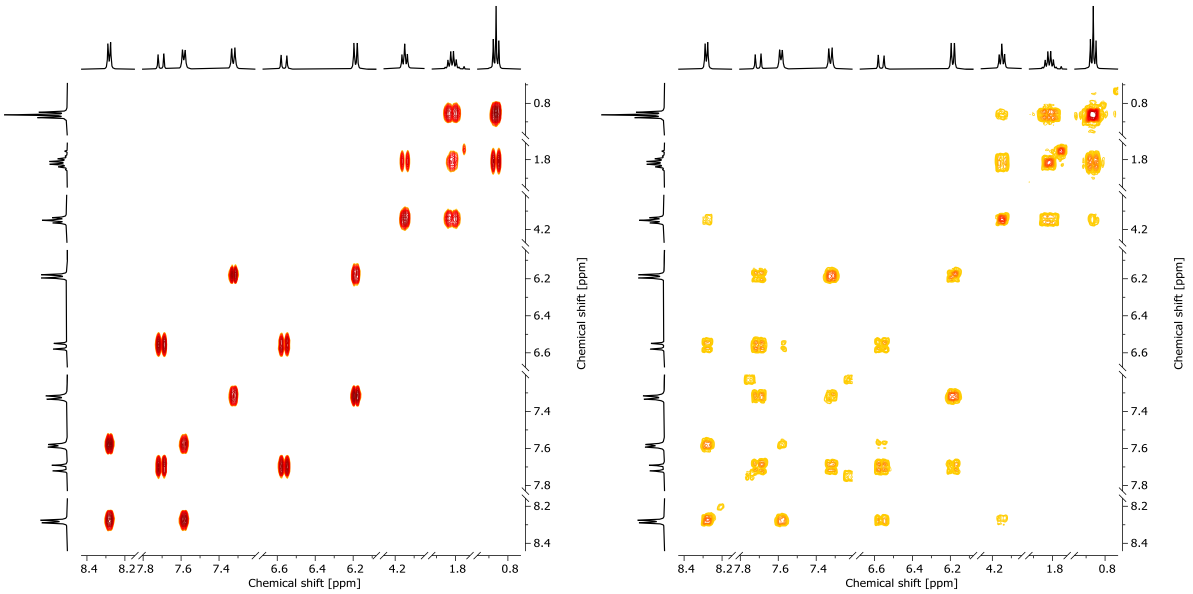

Fig. S17 Section of the ^{1}H-^{1}H COSY spectrum (left) and ^{1}H-^{1}H long range COSY spectrum (right, 450 ms) in the signal region of stilbazolium dye **5a**.

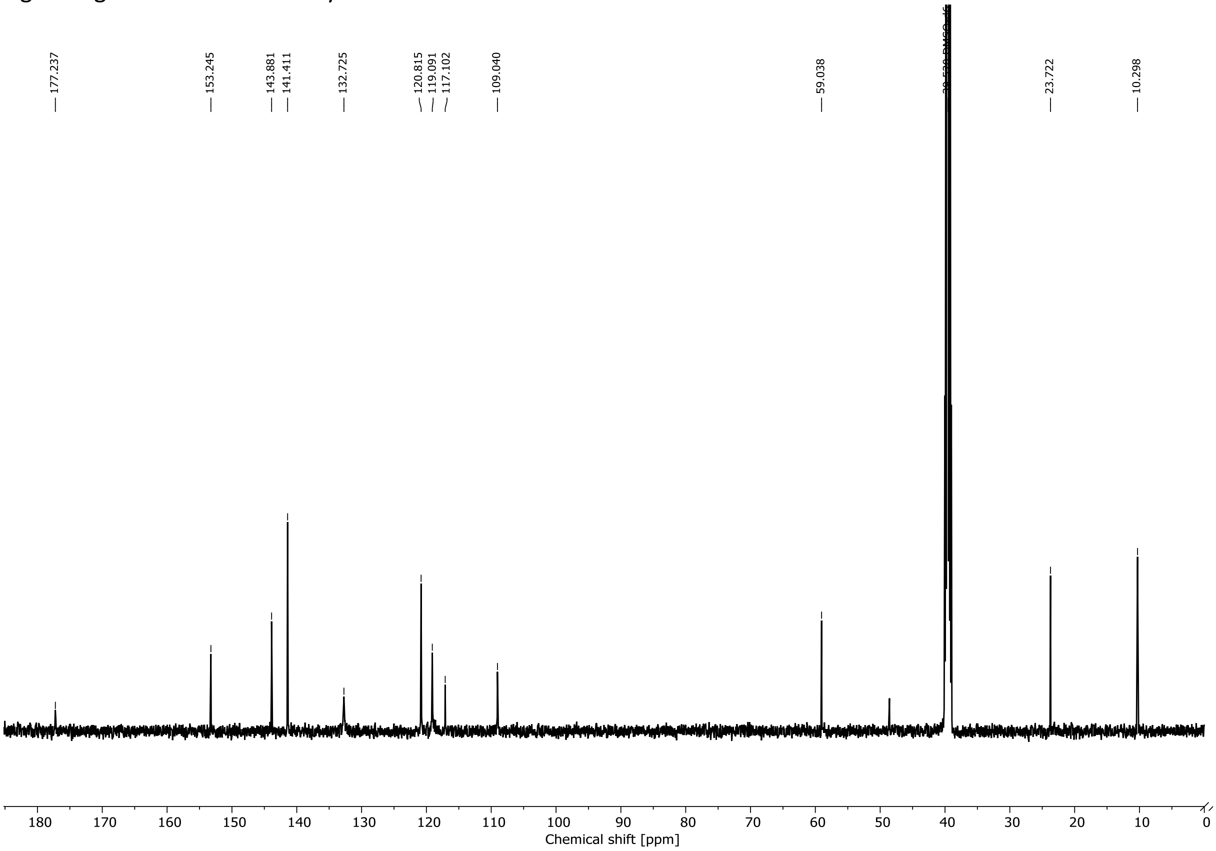

Fig. S18 ^{13}C{^{1}H} NMR (126 MHz, DMSO-d_6) spectrum of stilbazolium dye **5a**.

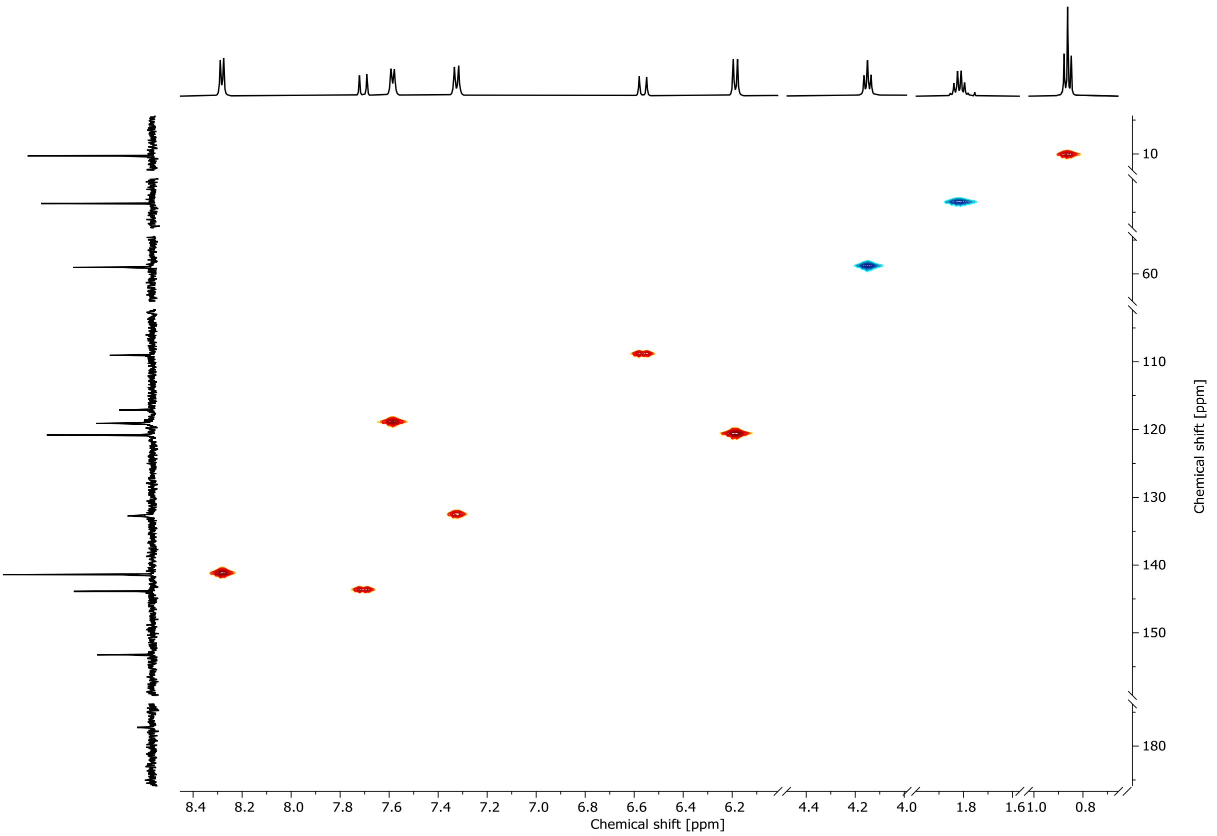

Fig. S19 Section of the ^{1}H-^{13}C HSQC spectrum in the signal region of stilbazolium dye **5a**.

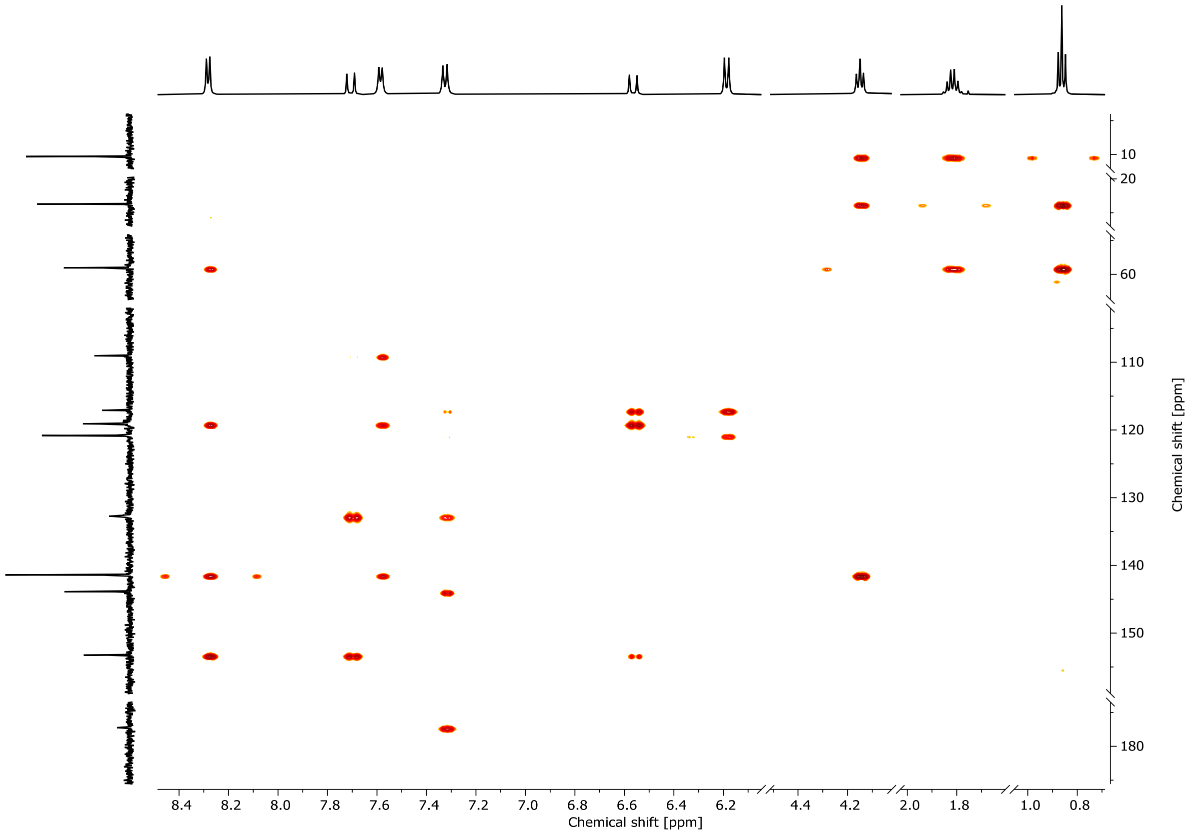

Fig. S20 Section of the ^{1}H-^{13}C HMBC spectrum in the signal region of stilbazolium dye **5a**.

Stilbazolium dye 5b

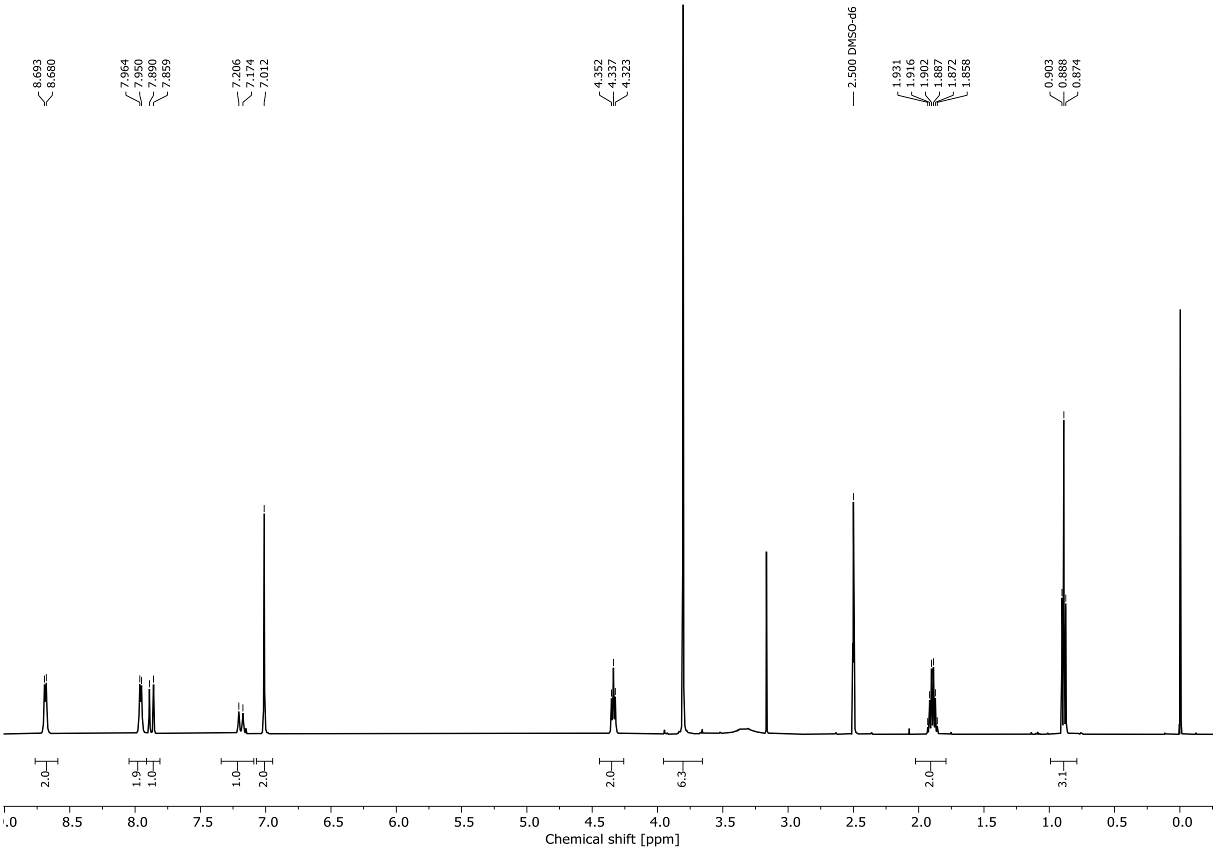

Fig. S21 ^{1}H NMR (500 MHz, DMSO-d_6) spectrum of stilbazolium dye **5b**.

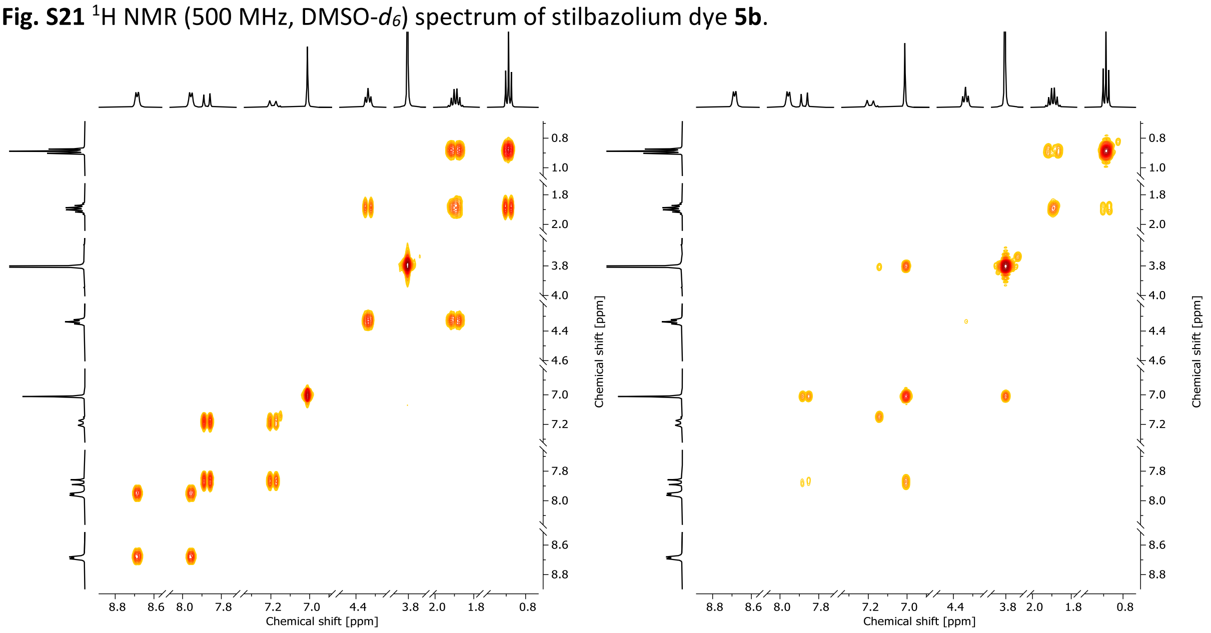

Fig. S22 Section of the ^{1}H-^{1}H COSY spectrum (left) and ^{1}H-^{1}H long range COSY spectrum (right, 450 ms) in the signal region of stilbazolium dye **5b**.

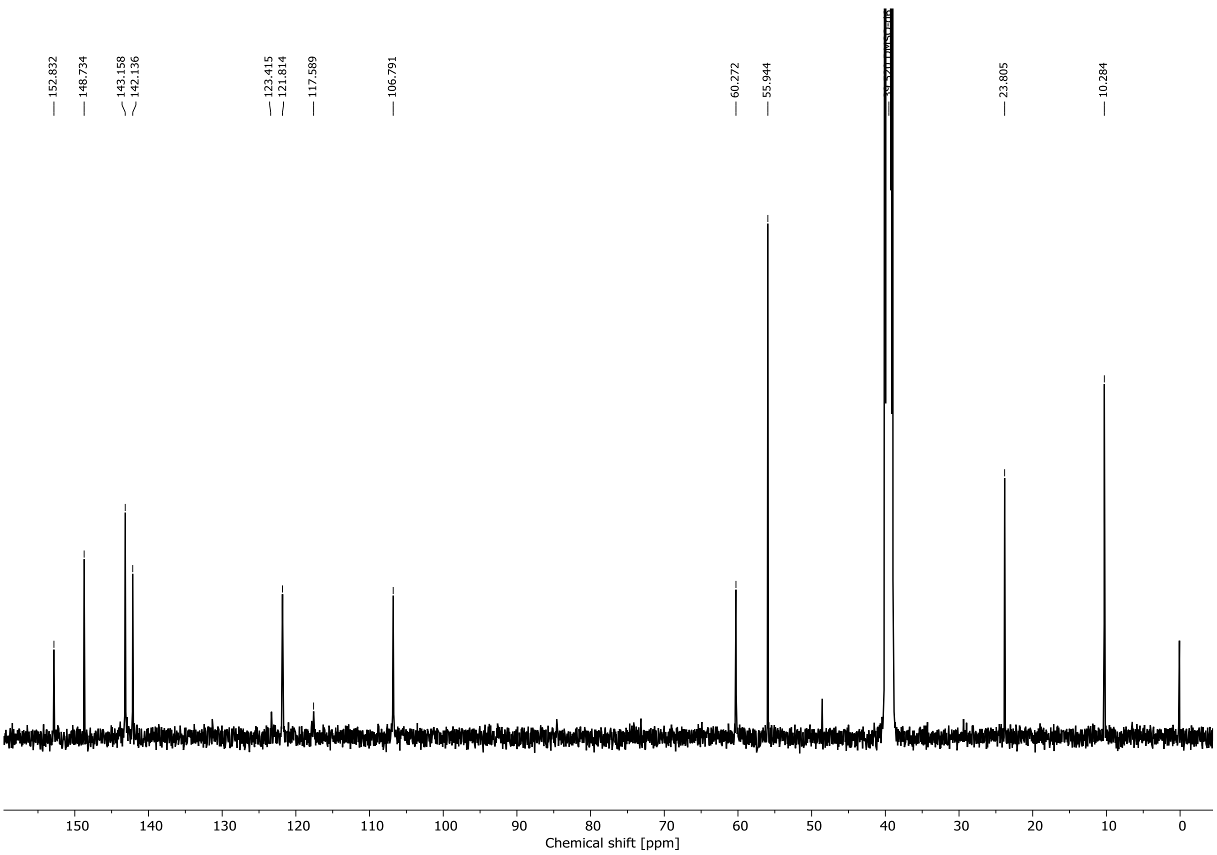

Fig. S23 $^{13}C\{^{1}H\}$ NMR (126 MHz, DMSO-d_6) spectrum of stilbazolium dye **5b**.

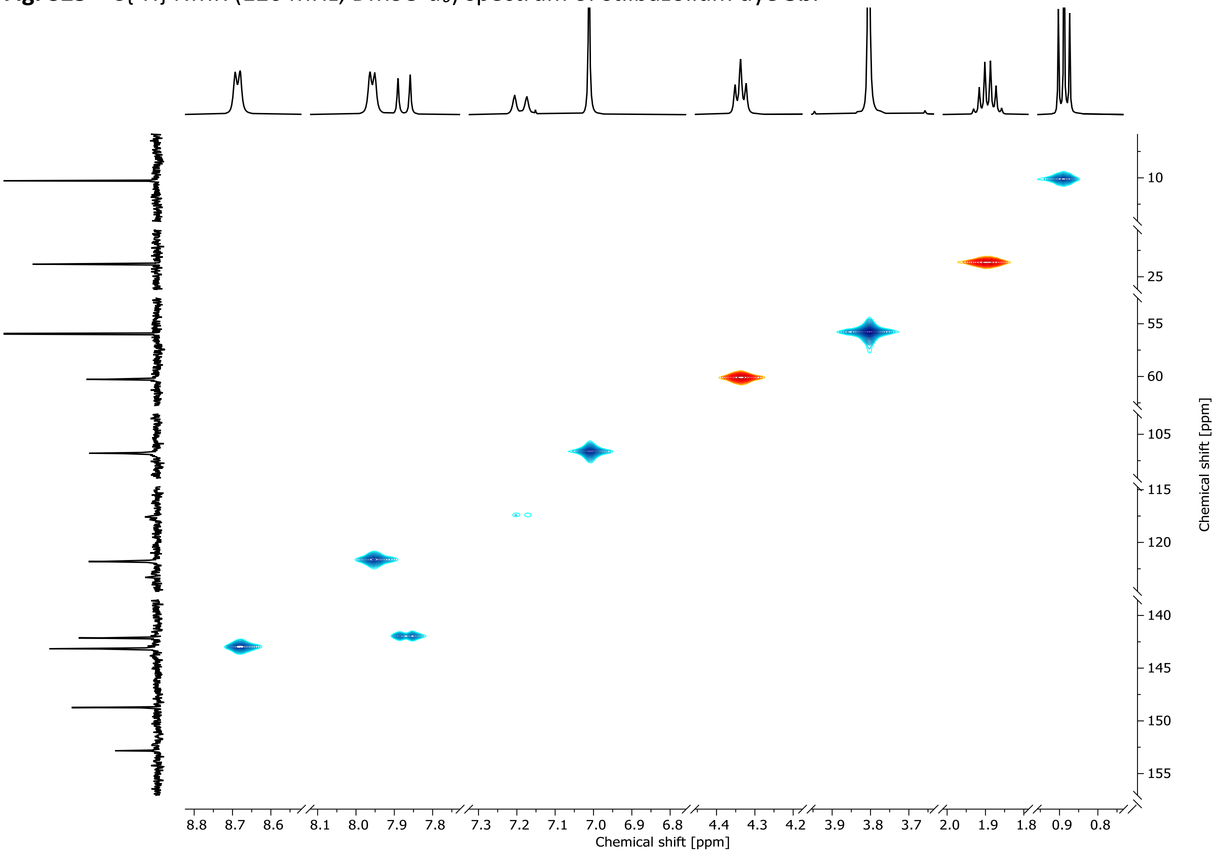

Fig. S24 Section of the ^{1}H-^{13}C HSQC spectrum in the signal region of stilbazolium dye **5b**.

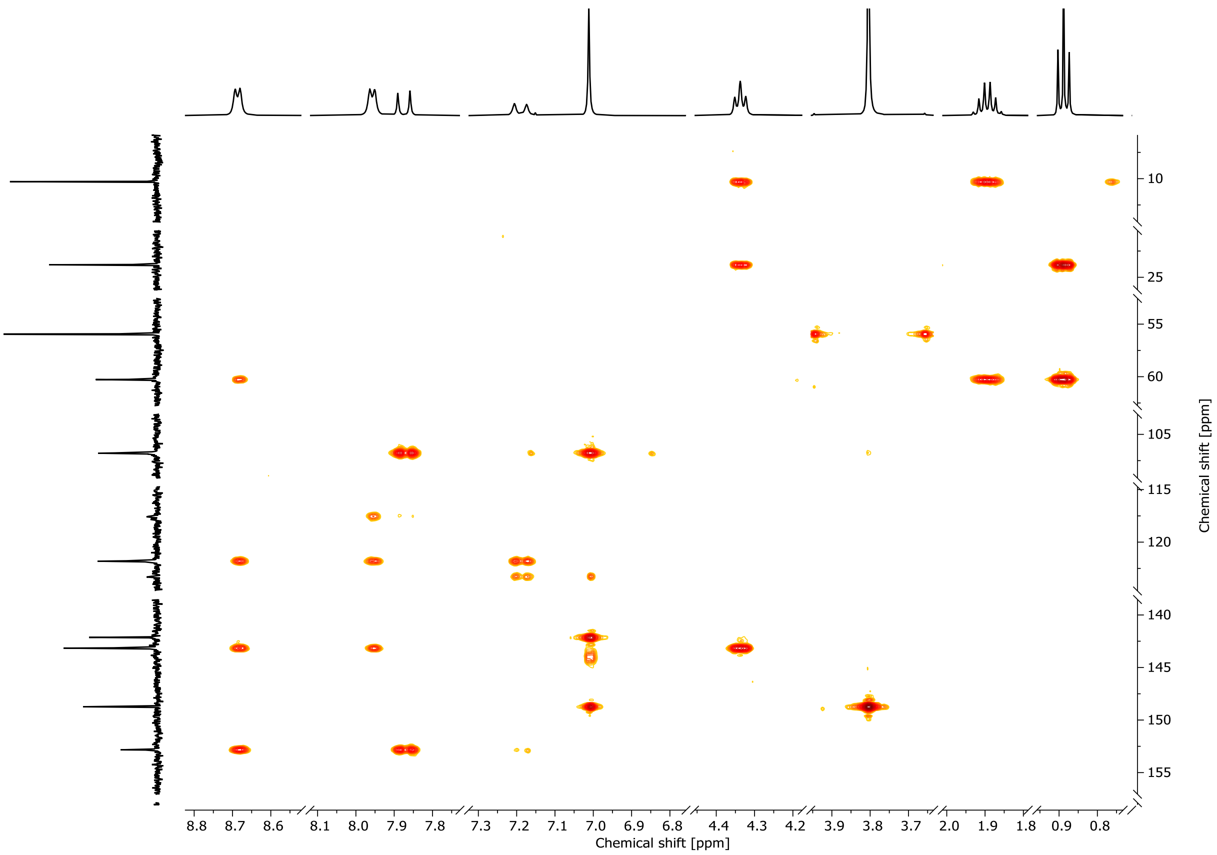

Fig. S25 Section of the ^{1}H-^{13}C HMBC spectrum in the signal region of stilbazolium dye **5b**.

Stilbazole 7a

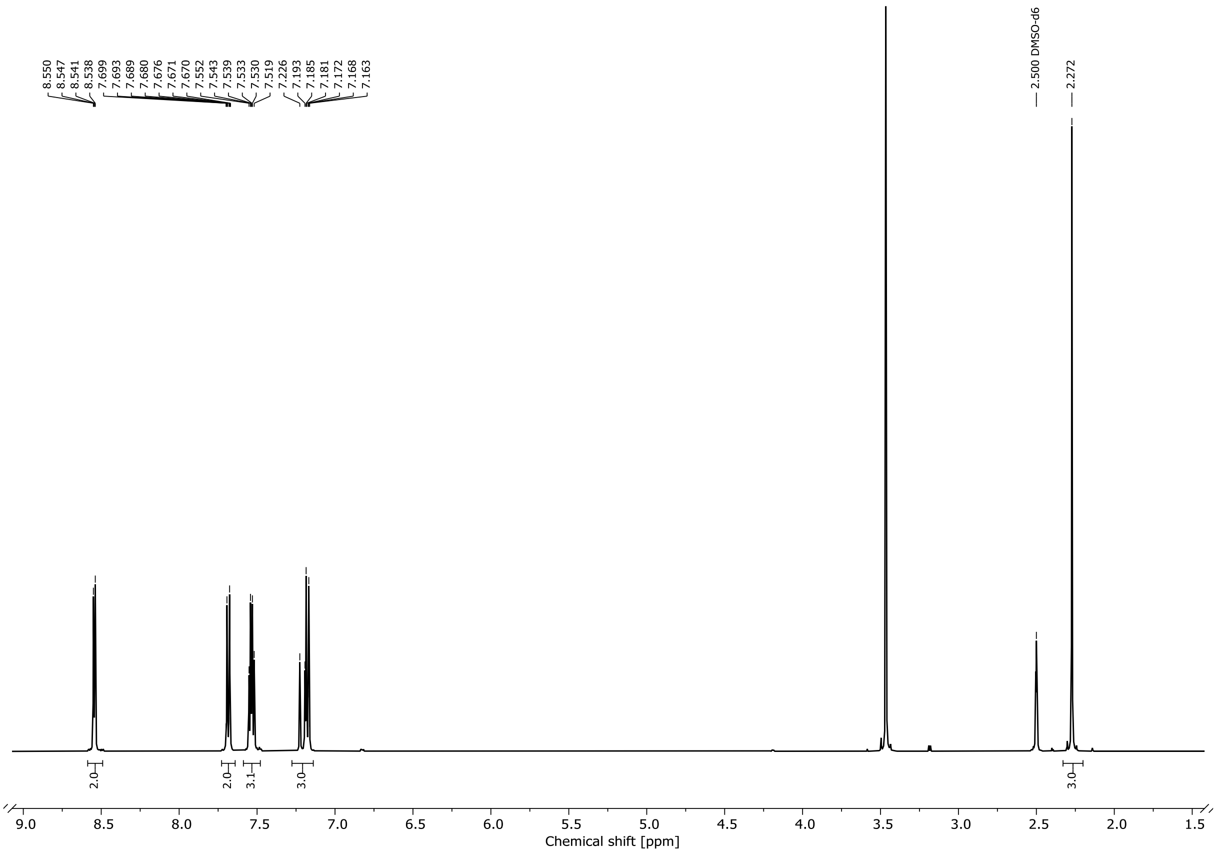

Fig. S26 ^{1}H NMR (500 MHz, DMSO-d_6) spectrum of stilbazole **7a**.

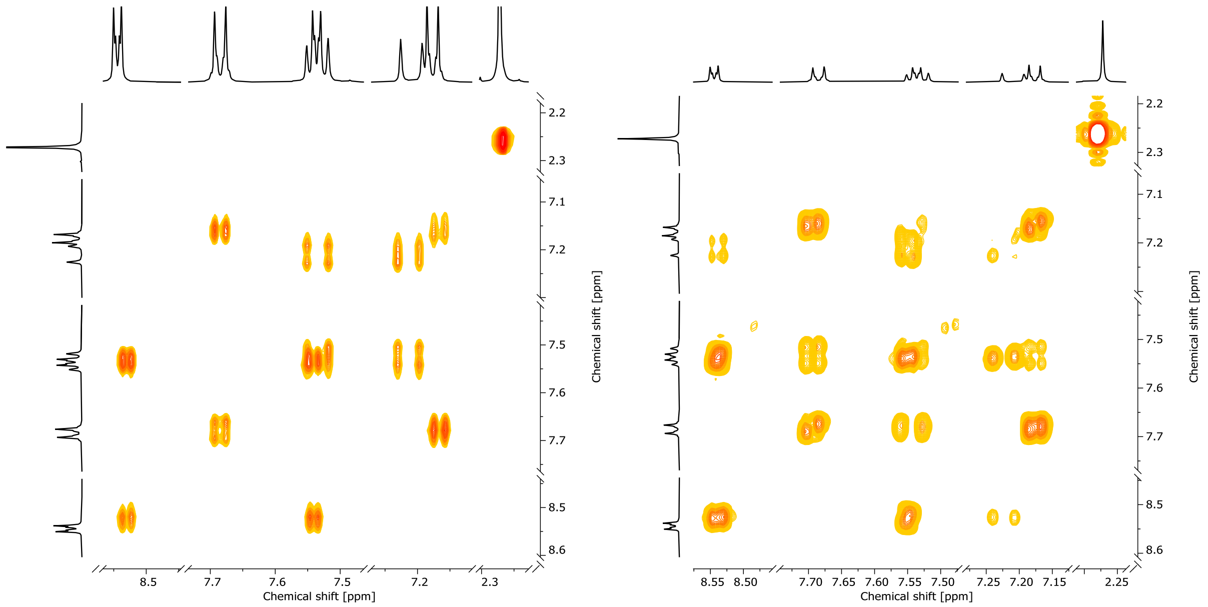

Fig. S27 Section of the ^{1}H-^{1}H COSY spectrum (left) and ^{1}H-^{1}H long range COSY spectrum (right, 450 ms) in the signal region of stilbazole **7a**.

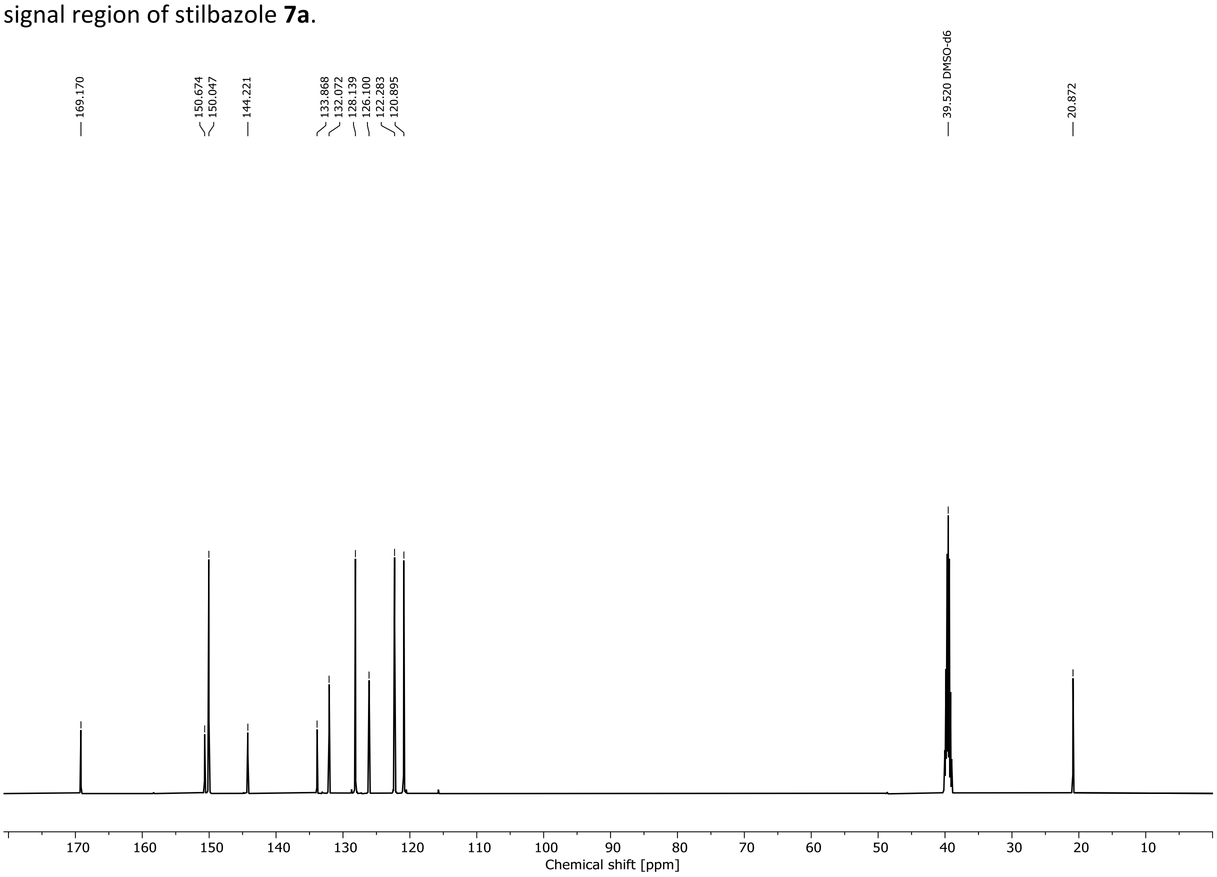

Fig. S28 $^{13}C\{^{1}H\}$ NMR (126 MHz, DMSO-d_6) spectrum of stilbazole **7a**.

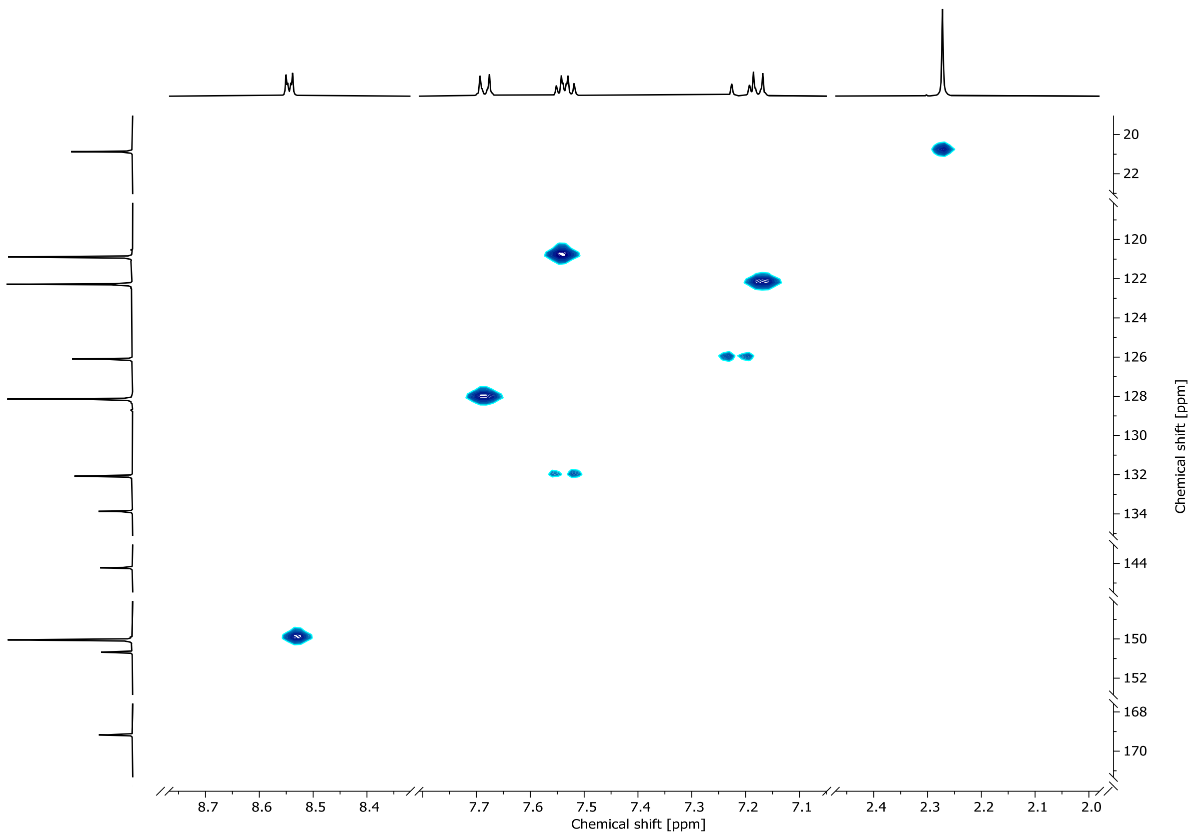

Fig. S29 Section of the ^{1}H-^{13}C HSQC spectrum in the signal region of stilbazole **7a**.

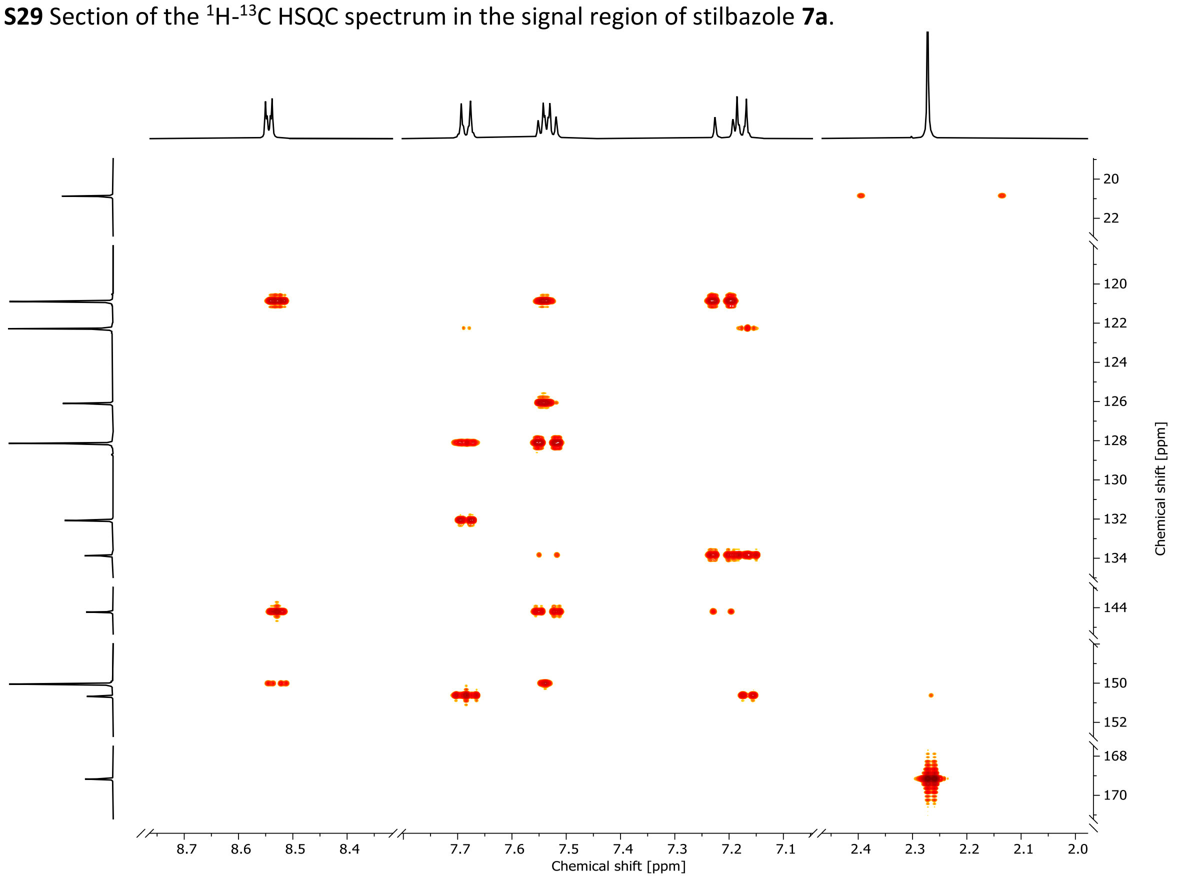

Fig. S30 Section of the ^{1}H-^{13}C HMBC spectrum in the signal region of stilbazole **7a**.

Stilbazole 7b

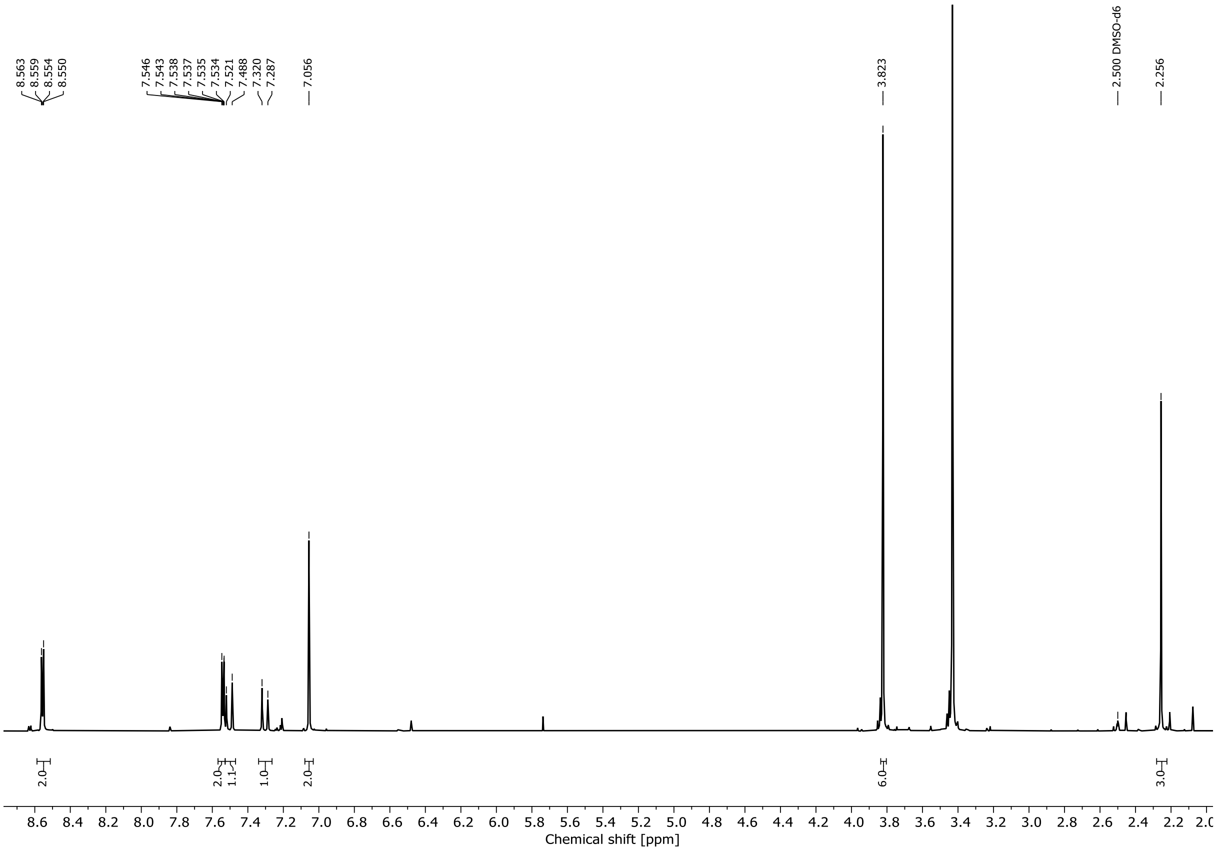

Fig. S31 ^{1}H NMR (500 MHz, DMSO-d_6) spectrum of stilbazole **7b**.

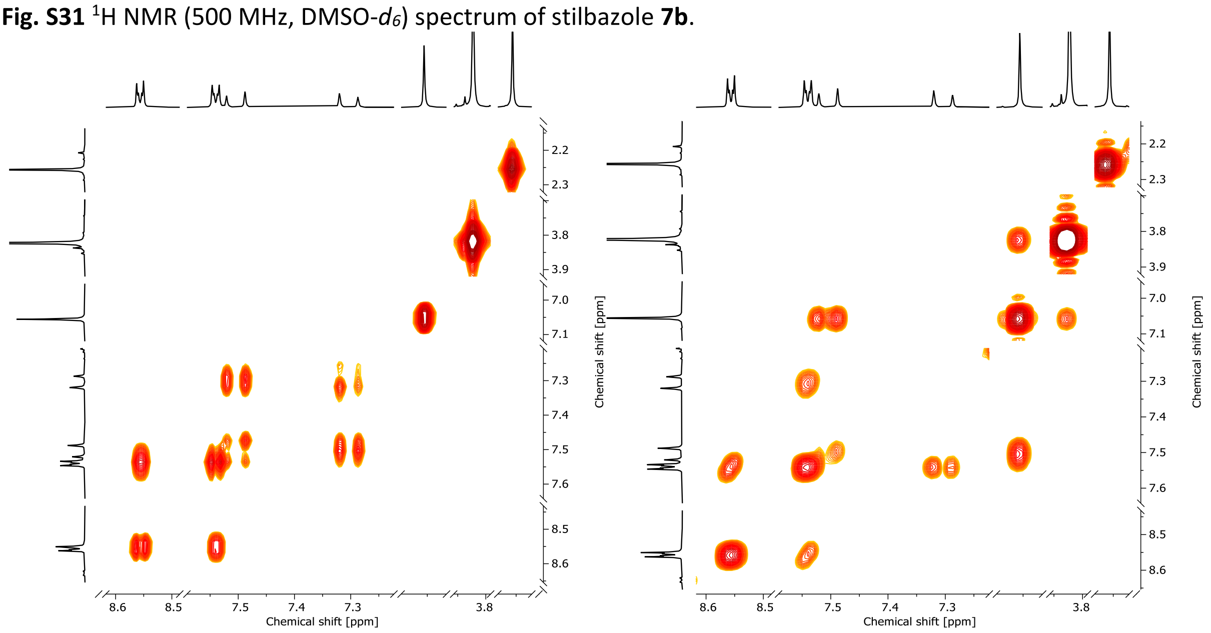

Fig. S32 Section of the ^{1}H-^{1}H COSY spectrum (left) and ^{1}H-^{1}H long range COSY spectrum (right, 450 ms) in the signal region of stilbazole **7b**.

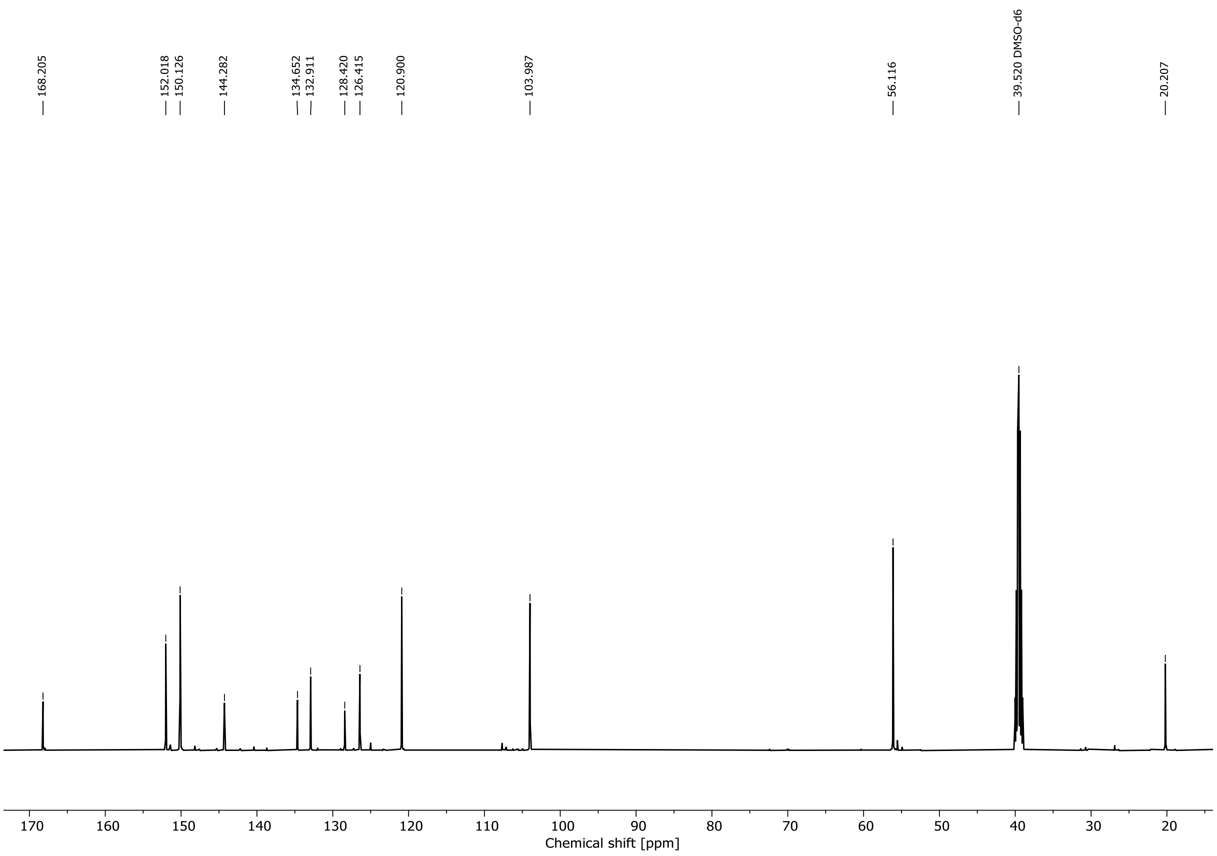

Fig. S33 $^{13}C\{^{1}H\}$ NMR (126 MHz, DMSO-d_6) spectrum of stilbazole **7b**.

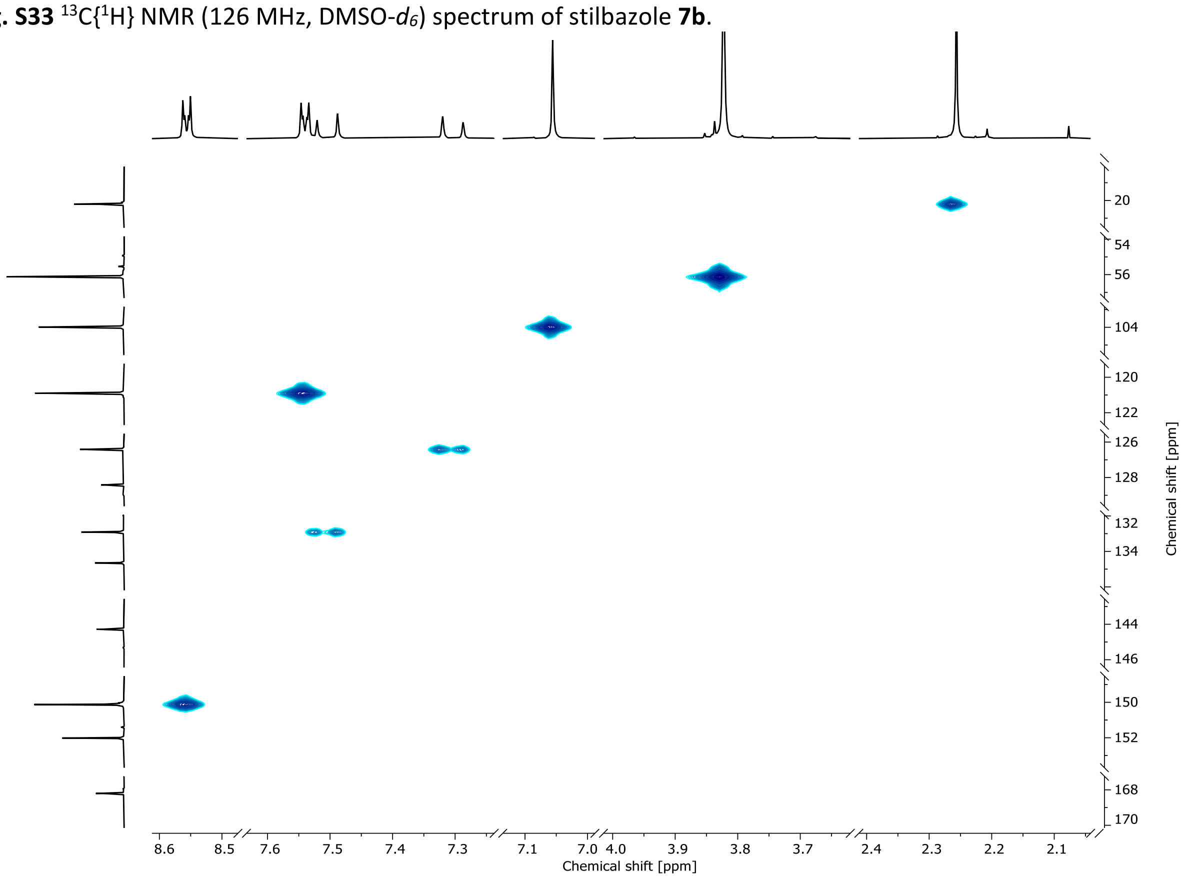

Fig. S34 Section of the ^{1}H-^{13}C HSQC spectrum in the signal region of stilbazole **7b**.

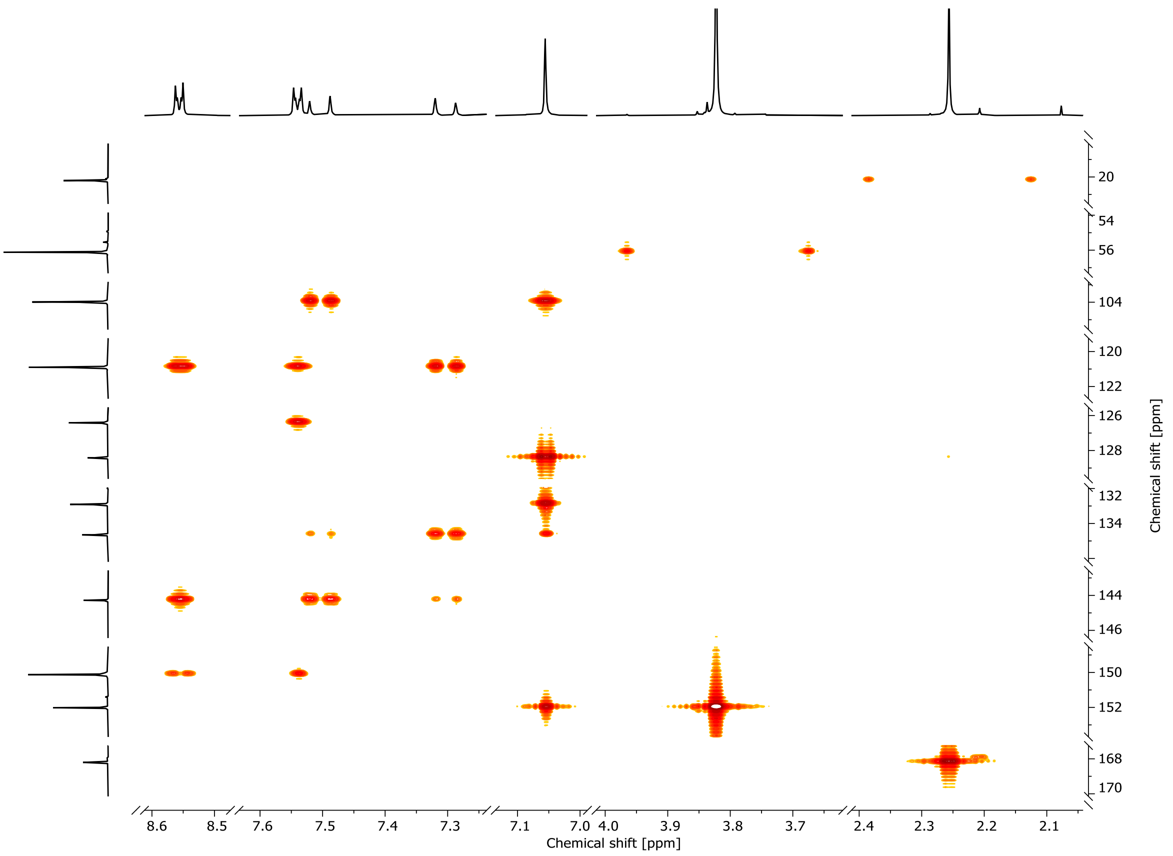

Fig. S35 Section of the ^{1}H-^{13}C HMBC spectrum in the signal region of stilbazole **7b**.

Stilbazolium salt 12a

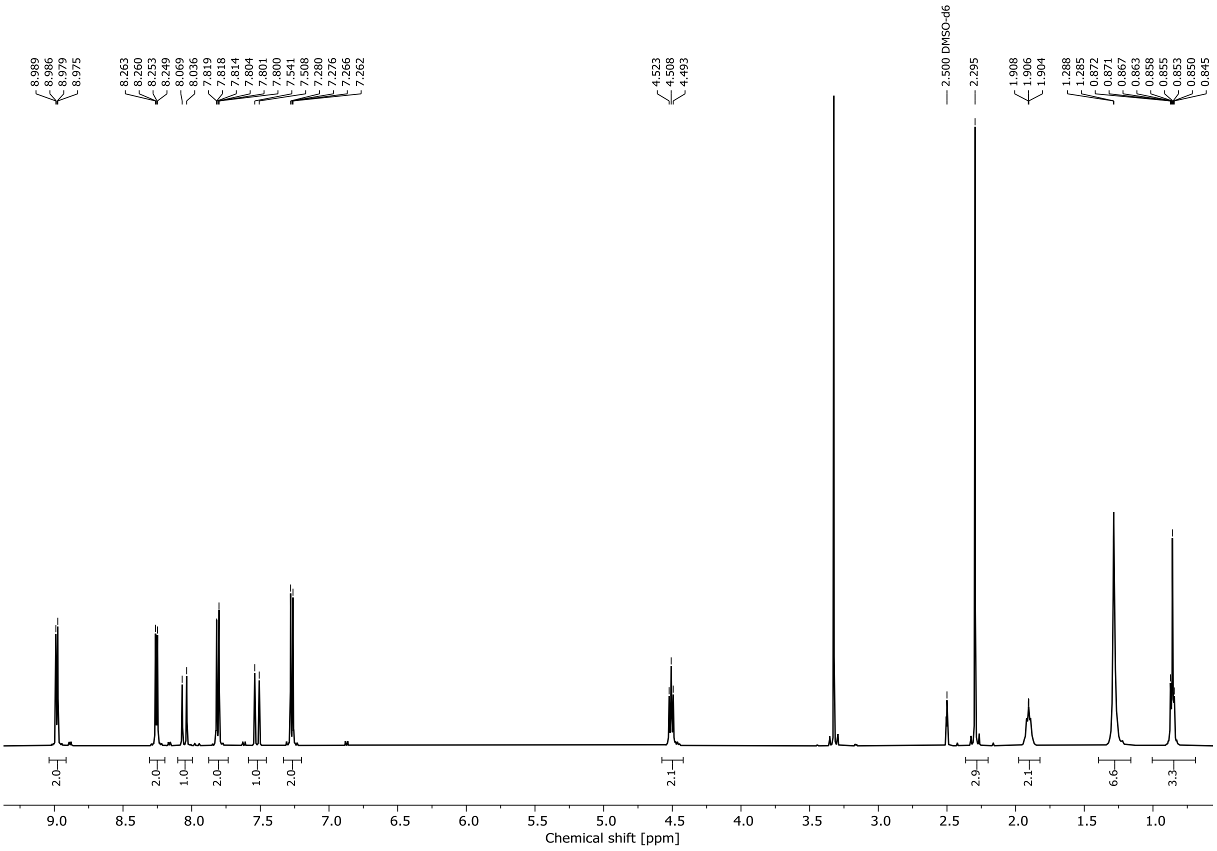

Fig. S36 ^{1}H NMR (500 MHz, DMSO-d_6) spectrum of stilbazolium salt **12a**.

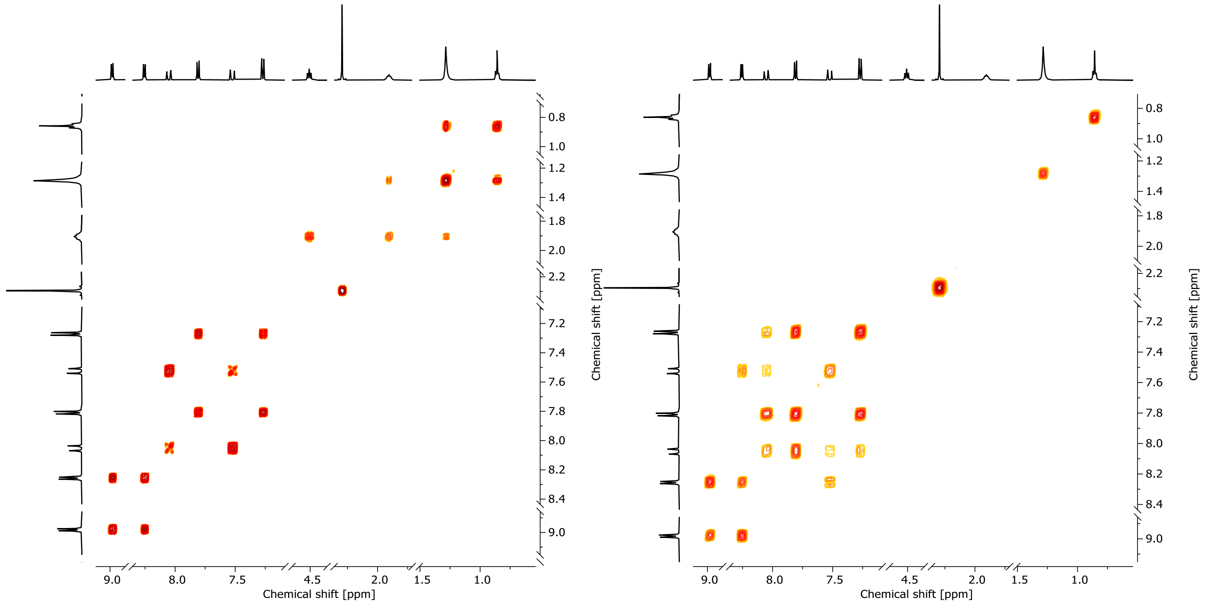

Fig. S37 Section of the ^{1}H-^{1}H COSY spectrum (left) and ^{1}H-^{1}H long range COSY spectrum (right, 450 ms) in the signal region of stilbazolium salt **12a**.

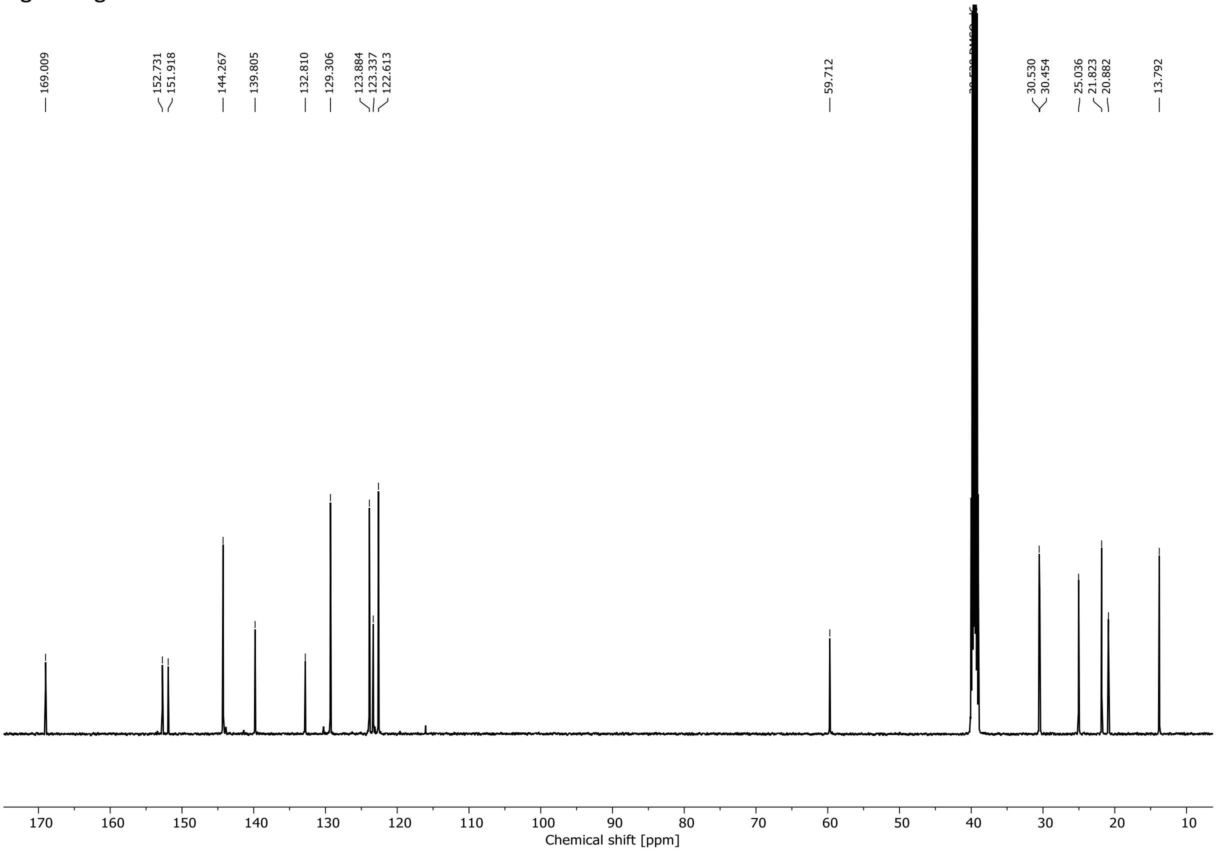

Fig. S38 $^{13}C\{^{1}H\}$ NMR (126 MHz, DMSO-d_6) spectrum of stilbazolium salt **12a**.

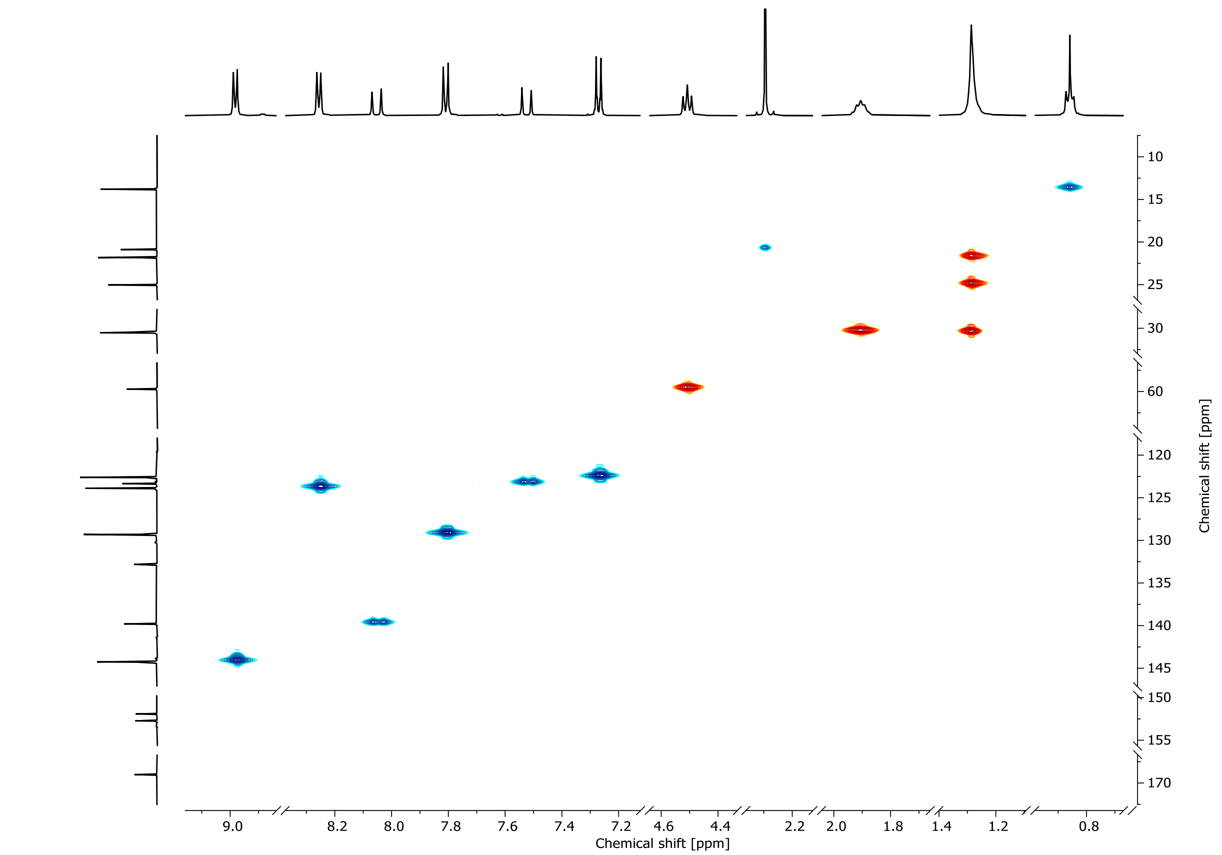

Fig. S39 Section of the ^{1}H-^{13}C HSQC spectrum in the signal region of stilbazolium salt **12a**.

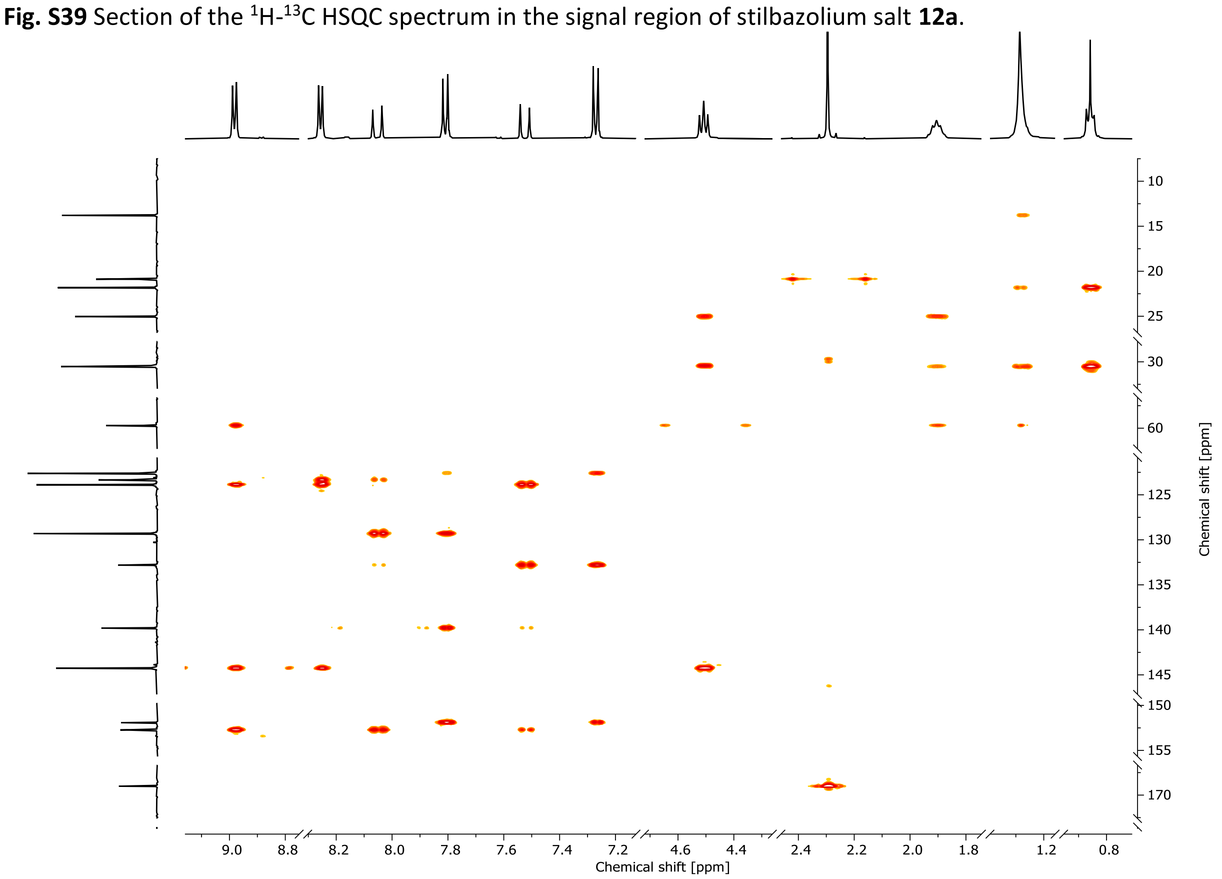

Fig. S40 Section of the ^{1}H-^{13}C HMBC spectrum in the signal region of stilbazolium salt **12a**.

Stilbazolium dye 13a

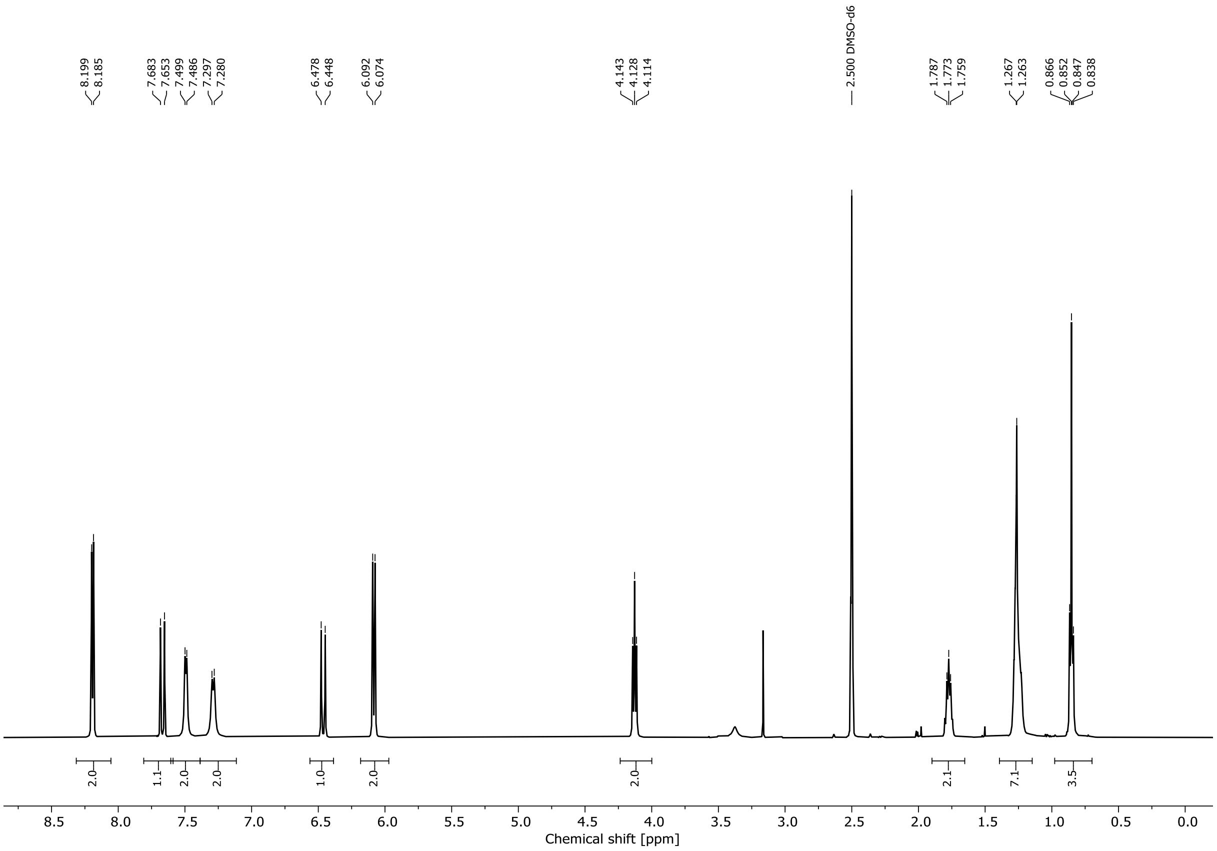

Fig. S41 ^1H NMR (500 MHz, DMSO-*d*$_6$) spectrum of stilbazolium dye **13a**.

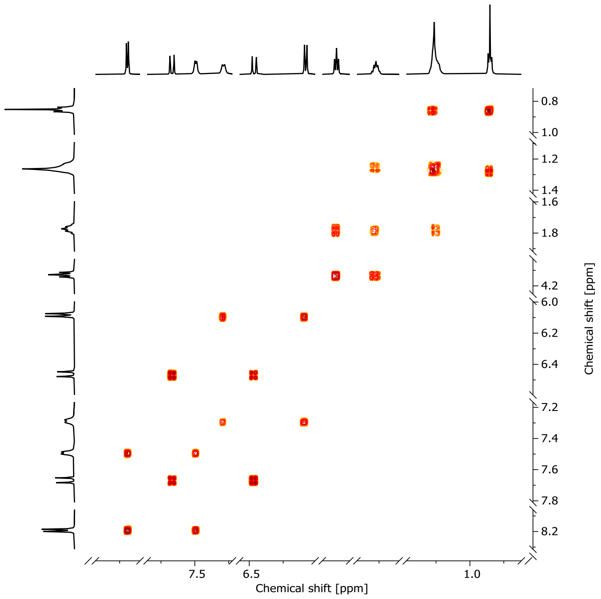

Fig. S42 Section of the ^1H-^1H COSY spectrum in the signal region of stilbazolium dye **13a**.

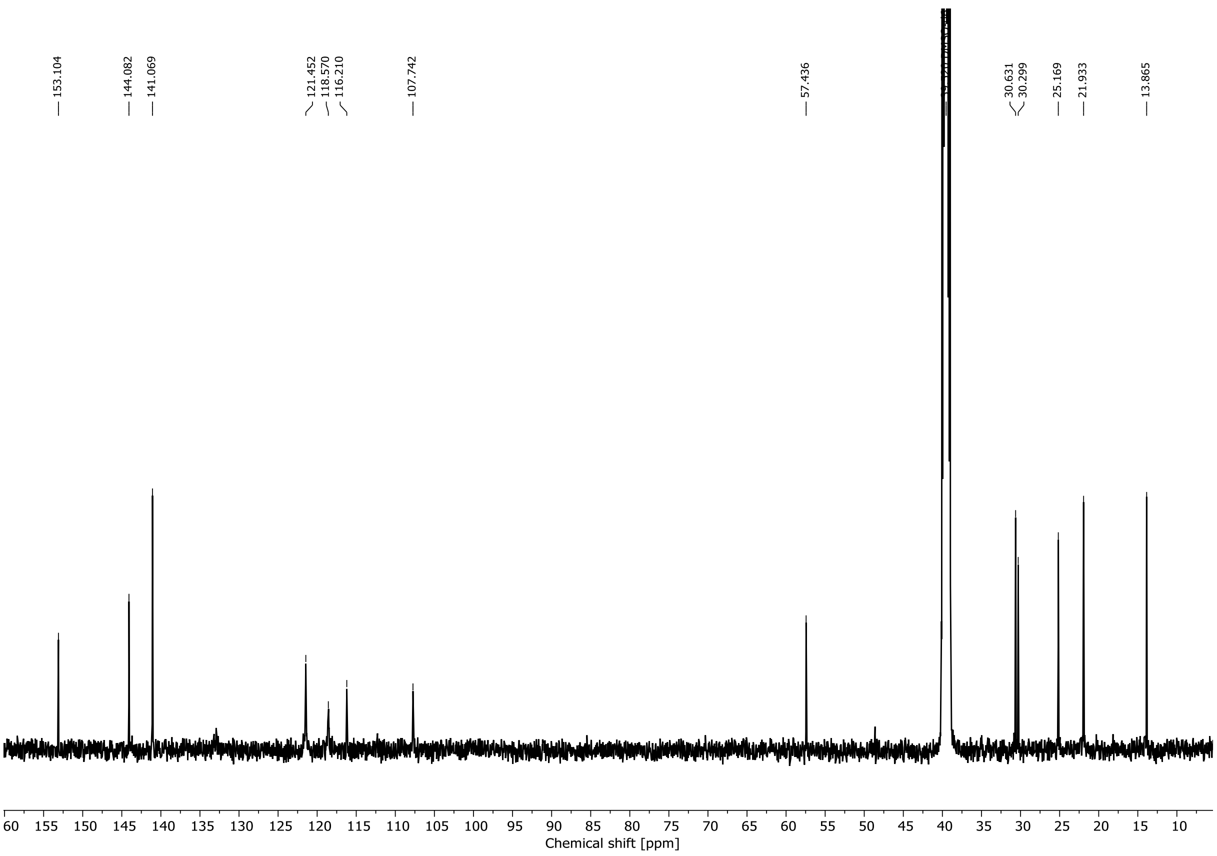

Fig. S43 $^{13}C\{^{1}H\}$ NMR (126 MHz, DMSO-d_6) spectrum of stilbazolium dye **13a**.

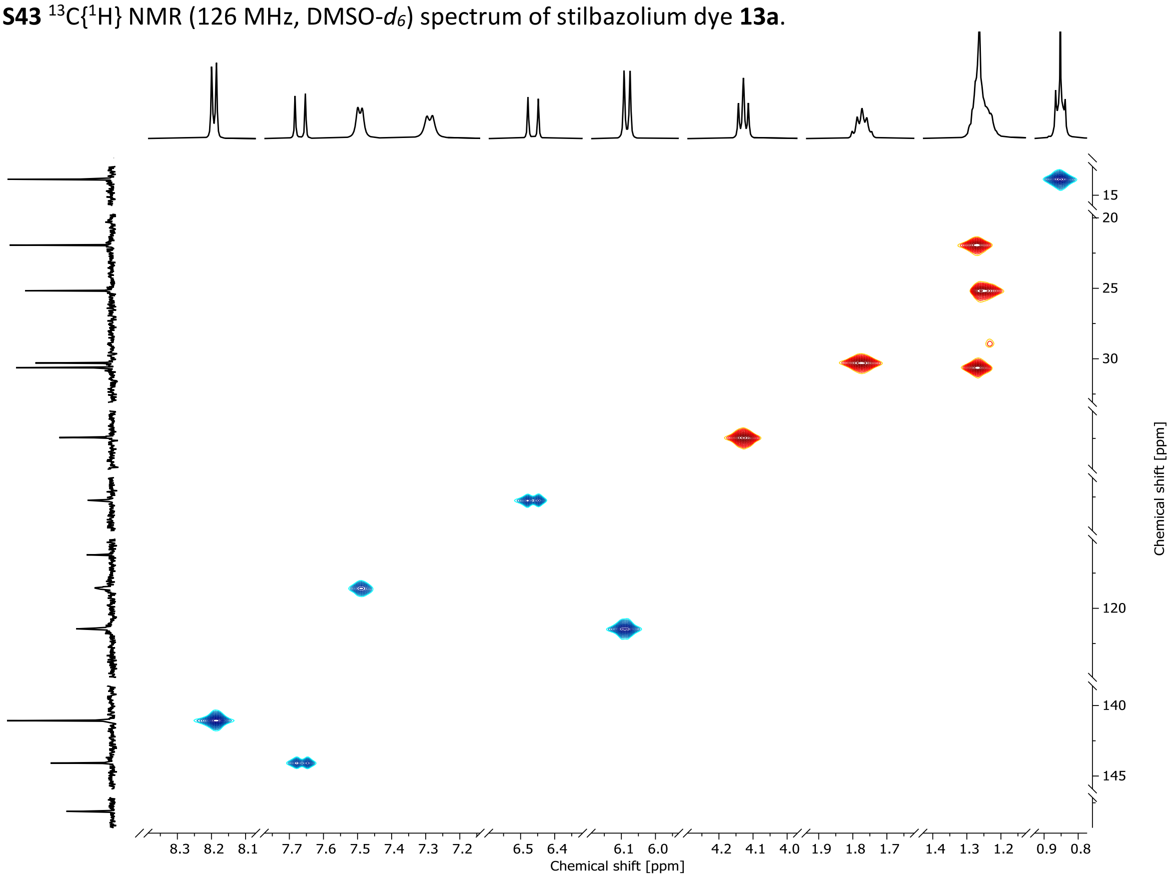

Fig. S44 Section of the ^{1}H-^{13}C HSQC spectrum in the signal region of stilbazolium dye **13a**.

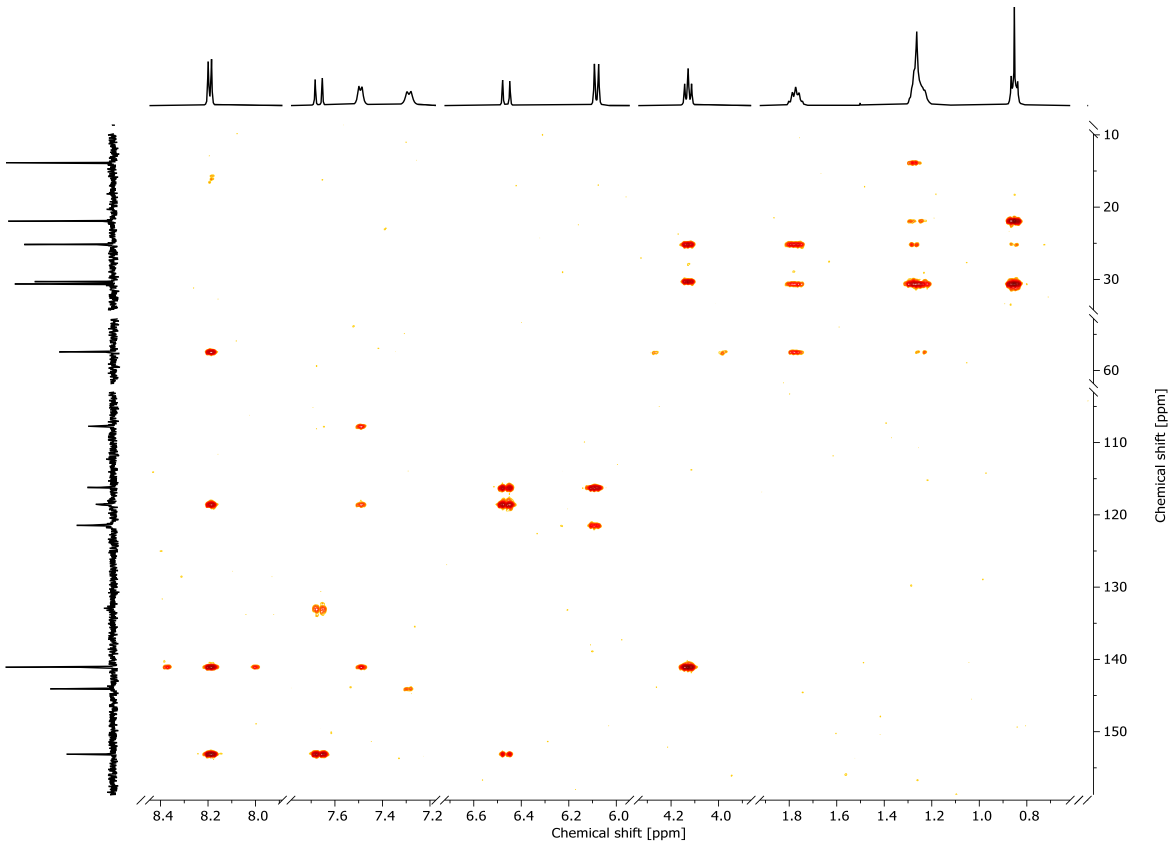

Fig. S45 Section of the ^{1}H-^{13}C HMBC spectrum in the signal region of stilbazolium dye **13a**.

3. Fluorosolvatochromic evaluation of model compounds 5

3.1 Preparation of samples for UV-Vis and fluorescence spectra measurement

Samples for UV-Vis and fluorescence emission spectra measurement were prepared as follows. An accurate amount of the stilbazolium dye **5** was weighed and transferred to a volumetric flask (100 mL) using MeOH:DCM 9:1 v/v. The solution was diluted with methanol to the exact volume. Aliquots (1 mL) of the prepared solution were pipetted into glass vials. The solutions were subsequently evaporated with a stream of nitrogen and dried under reduced pressure. Stock solutions were prepared from the thus prepared samples by dissolving the residue in the exact volume (1 mL) of the studied solvent. The stock solutions were used for UV-Vis dilution experiments, which were performed either in a single solvent or in a binary solvent mixture. The resulting concentrations of stock solutions and measured solutions are shown in Table S1.

Table S1: Preparation of solutions for UV-Vis titration experiments.

	Stilbazolium salt **5a**	Stilbazolium salt **5b**
m_{sample} [mg]	22.5	28.3
$\rho_{stock\ solution}$ [mg·mL^{-1}]	0.225	0.283
$c_{stock\ solution}$ [mmol·L^{-1}]	0.9402	0.9453
c_1 [µmol·L^{-1}]	12.21	12.28
c_2 [µmol·L^{-1}]	15.00	15.07
c_3 [µmol·L^{-1}]	17.72	17.79
c_4 [µmol·L^{-1}]	20.38	20.44

<u>a) Measurement of UV-Vis spectra in a single solvent:</u> The samples for measurement were prepared as follows. Volume of 1900 µL of the studied solvent was pipetted into the cuvette. The cuvette was closed with a cap. The UV-Vis spectrum was measured (blank; measured three times and then averaged). A blank was used to correct the measured spectra. Next, volume of 25 µL of a stock solution of the studied solvent was added to the cuvette (see samples preparation, stock solution). The cuvette was closed with a cap. The solution was mixed properly. The UV-Vis spectrum was recorded (c_1). With the standard addition of 25 µL of the diluted solution, a total of four points were measured (c_1 to c_4). The obtained spectra are shown below. Between measurements, the cuvette was washed with methanol and acetone, and then dried with a stream of air.

<u>b) Measurement of UV-Vis spectra in a solvent mixture:</u> The samples for measurement were prepared as follows. Volume of 1900 µL of the studied solvent was pipetted into the cuvette. The cuvette was closed with a cap. The UV-Vis spectrum was measured (blank; measured twice and then averaged). A blank was used to correct the measured spectra. Next, volume of 25 µL of stock solution of stilbazolium dye **5** in DMSO or methanol was added to the cuvette (see samples preparation, stock solution). The cuvette was closed with a cap. The solution was mixed properly. The UV-Vis spectrum was recorded (c_1). With the standard addition of 25 uL of the stock solution of stilbazolium dye **5** in DMSO (or MeOH), a total of four points were measured (c_1 to c_4). The obtained spectra are shown below. Between measurements, the cuvette was washed with methanol and acetone and then dried with a stream of air.

Fluorescence emission spectra were measured for each sample at a concentration c_1.

3.2 UV-Vis spectra of stilbazolium dyes 5

*3.2.1 UV-Vis spectra of stilbazolium dye **5a** measured in a single solvent*

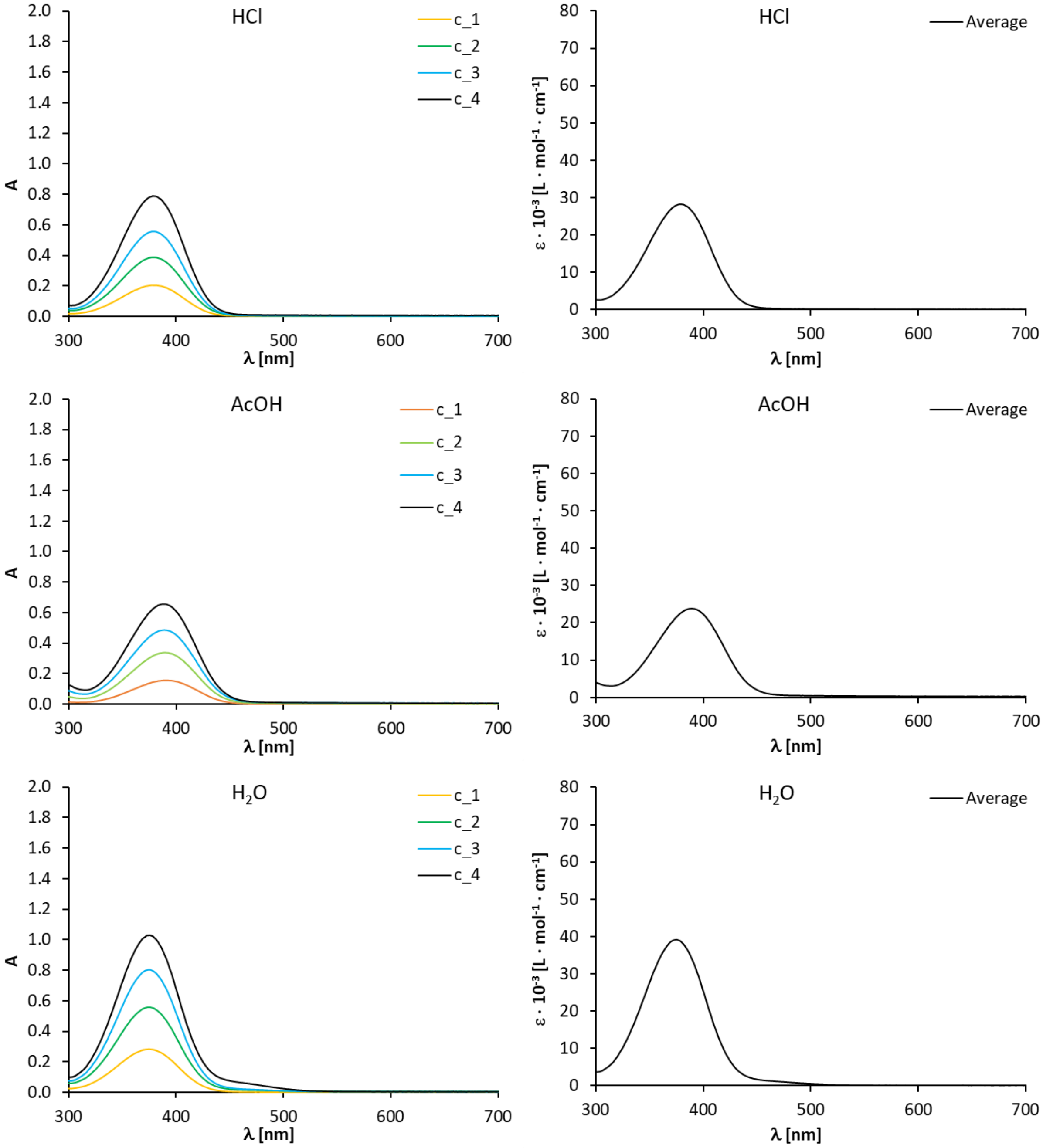

Fig. S46 UV-Vis spectra of stilbazolium dye **5a** in various solvents. Left: Titration experiments. Right: Averaged UV-Vis spectrum obtained from the spectra from titration experiments after their conversion to concentration-independent spectra. The measured concentrations were as follows: c_1 = 12.21 µmol·L^{-1}, c_2 = 15.00 µmol·L^{-1}, c_3 = 17.72 µmol·L^{-1}, c_4 = 20.38 µmol·L^{-1} (see Table S1).

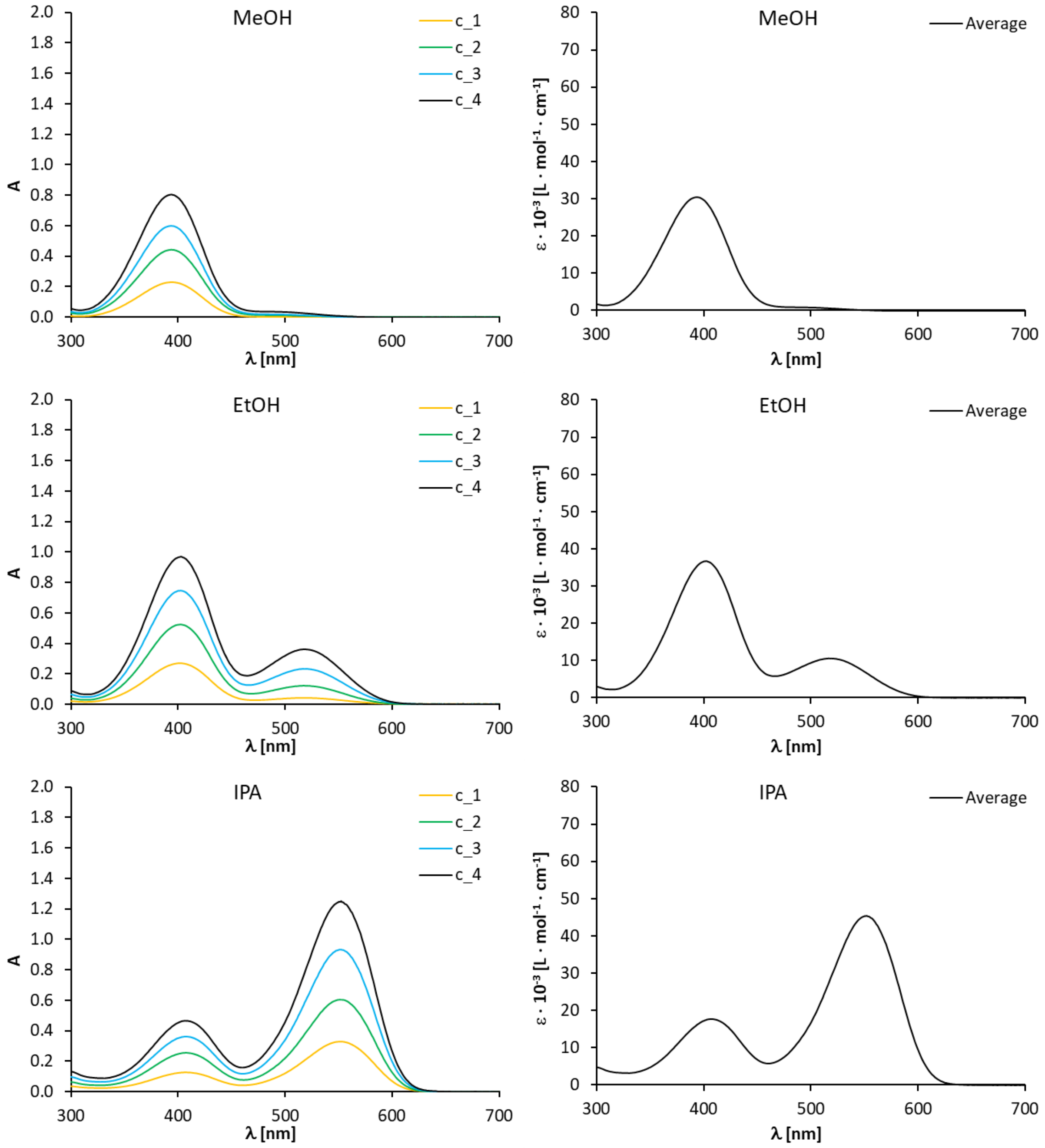

Fig. S47 UV-Vis spectra of stilbazolium dye **5a** in various solvents. Left: Titration experiments. Right: Averaged UV-Vis spectrum obtained from the spectra from titration experiments after their conversion to concentration-independent spectra. The measured concentrations were as follows: c_1 = 12.21 µmol·L^{-1}, c_2 = 15.00 µmol·L^{-1}, c_3 = 17.72 µmol·L^{-1}, c_4 = 20.38 µmol·L^{-1} (see Table S1).

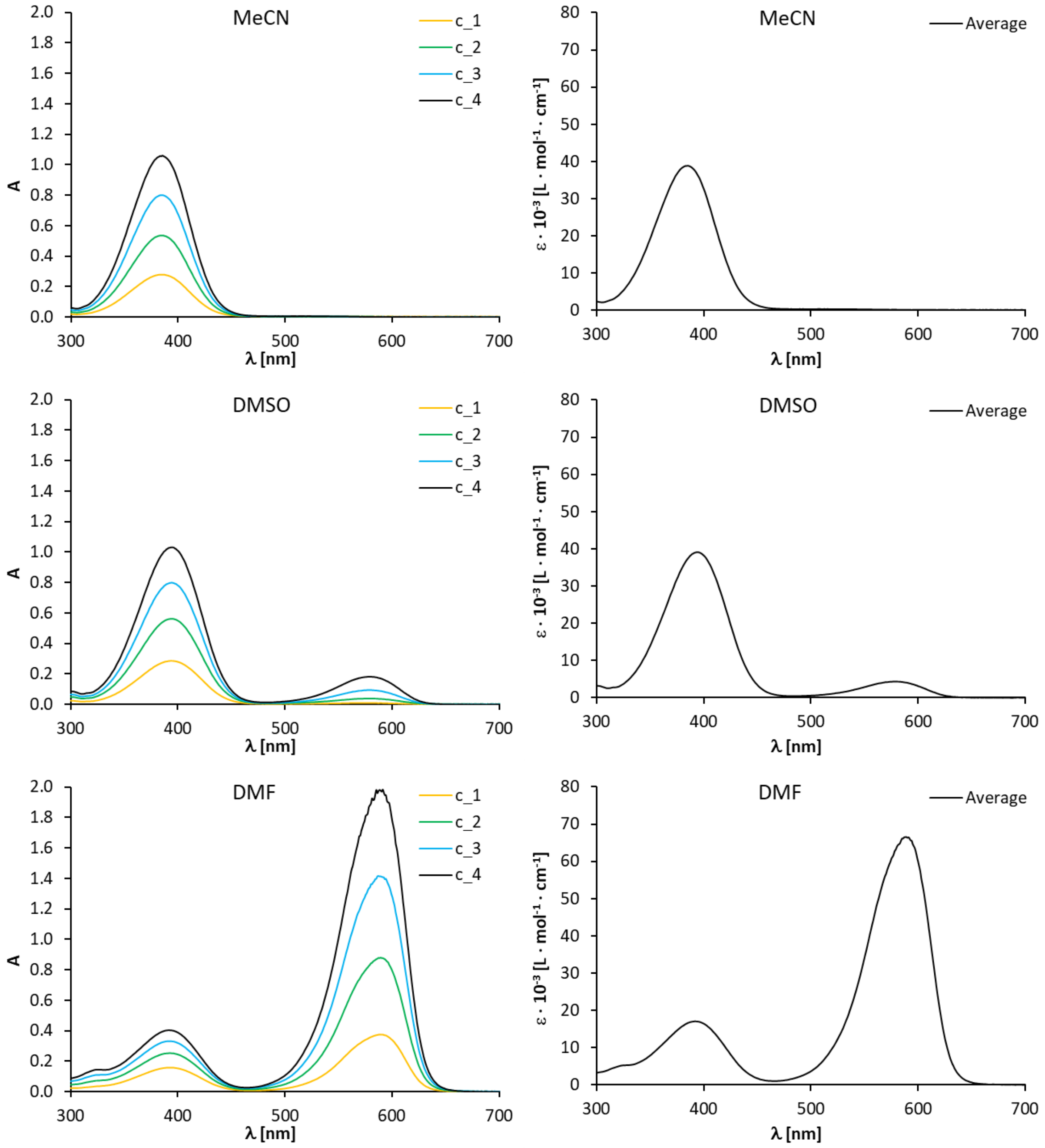

Fig. S48 UV-Vis spectra of stilbazolium dye **5a** in various solvents. Left: Titration experiments. Right: Averaged UV-Vis spectrum obtained from the spectra from titration experiments after their conversion to concentration-independent spectra. The measured concentrations were as follows: c_1 = 12.21 µmol·L^{-1}, c_2 = 15.00 µmol·L^{-1}, c_3 = 17.72 µmol·L^{-1}, c_4 = 20.38 µmol·L^{-1} (see Table S1).

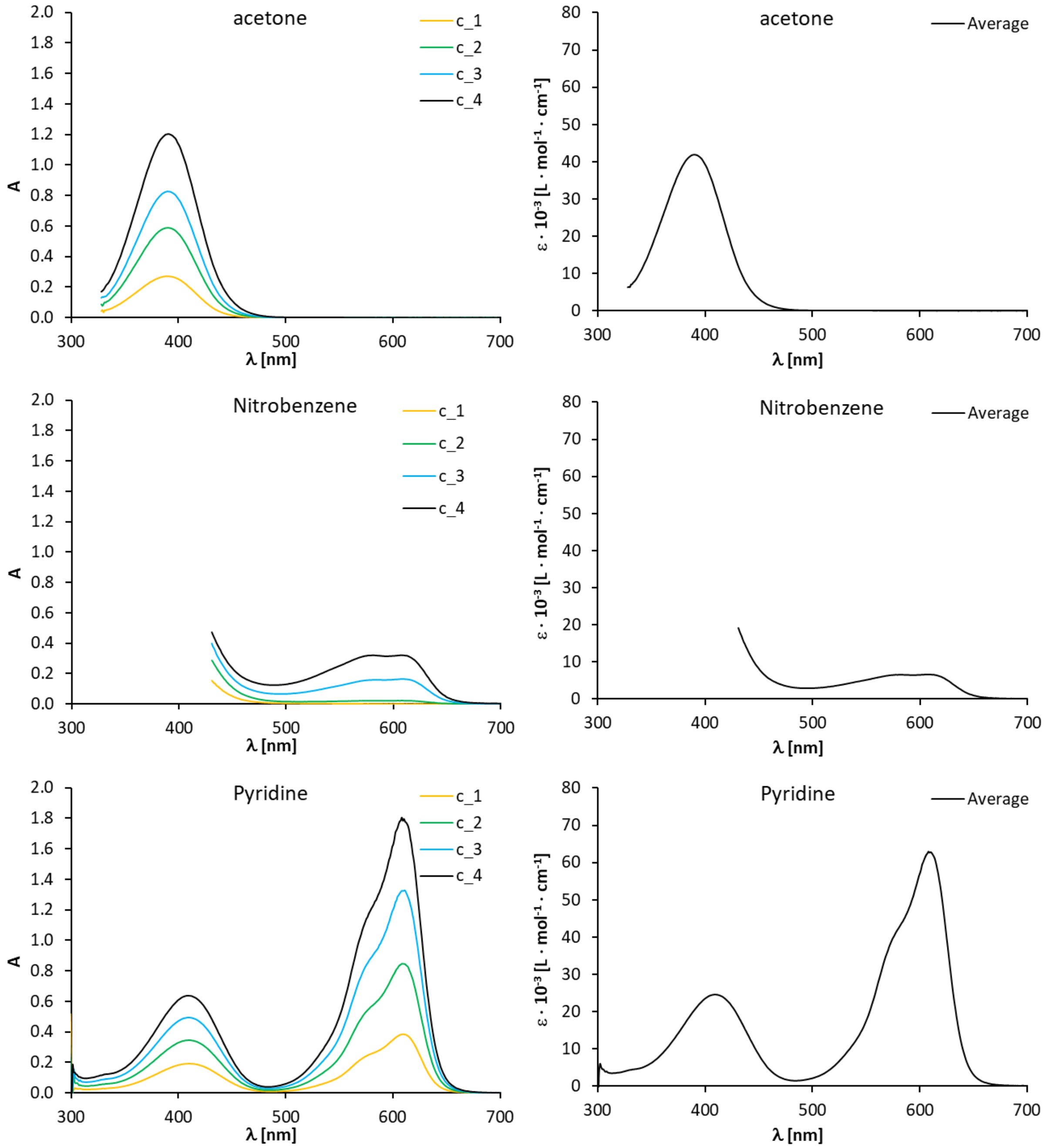

Fig. S49 UV-Vis spectra of stilbazolium dye **5a** in various solvents. Left: Titration experiments. Right: Averaged UV-Vis spectrum obtained from the spectra from titration experiments after their conversion to concentration-independent spectra. In the case of high solvent absorption, the spectrum was clipped (acetone 338 nm; nitrobenzene 430 nm). The measured concentrations were as follows: c_1 = 12.21 µmol·L^{-1}, c_2 = 15.00 µmol·L^{-1}, c_3 = 17.72 µmol·L^{-1}, c_4 = 20.38 µmol·L^{-1} (see Table S1).

*3.2.2 UV-Vis spectra of stilbazolium dye **5b** measured in a single solvent*

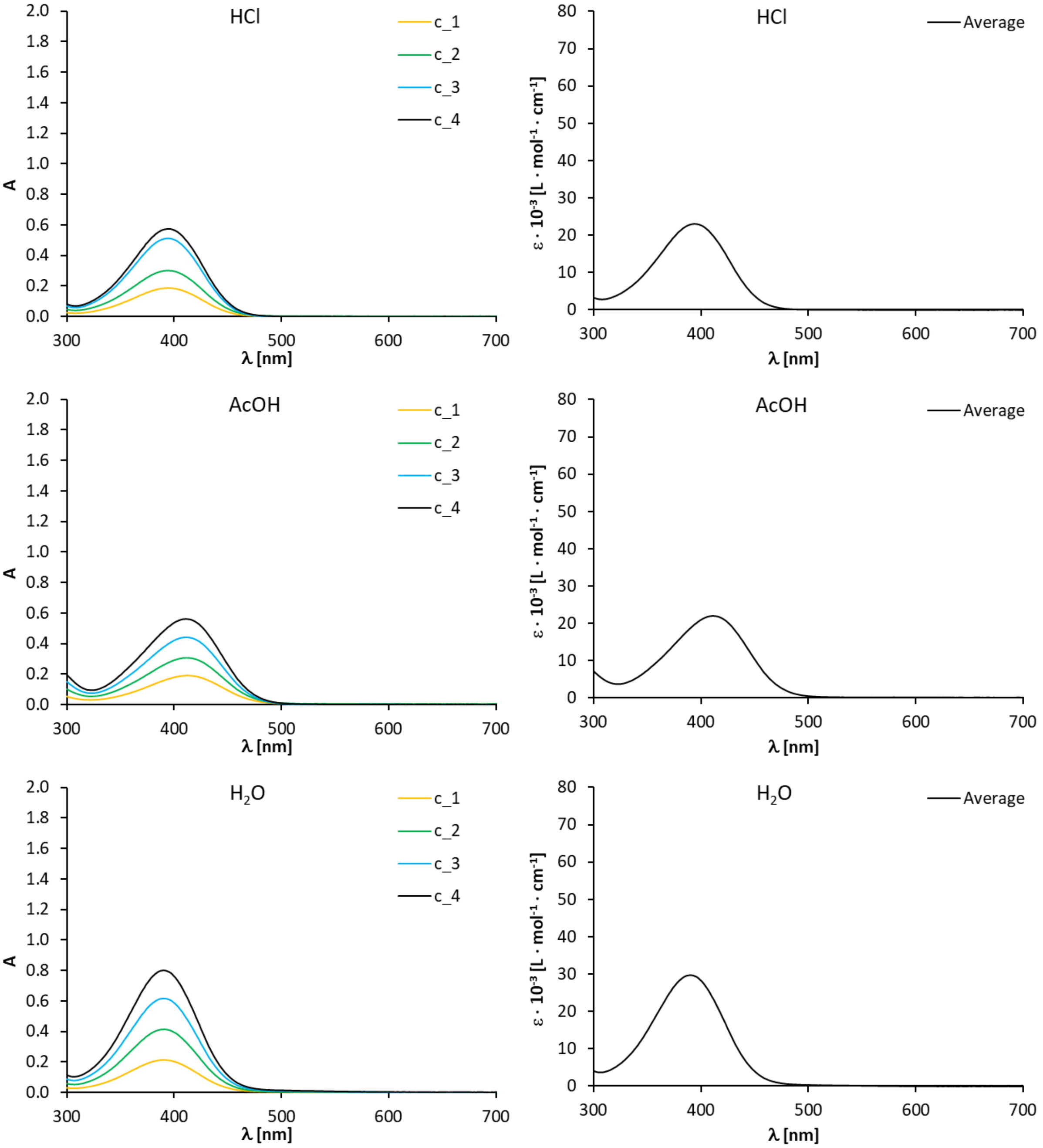

Fig. S50 UV-Vis spectra of stilbazolium dye **5b** in various solvents. Left: Titration experiments. Right: Averaged UV-Vis spectrum obtained from the spectra from titration experiments after their conversion to concentration-independent spectra. The measured concentrations were as follows: c_1 = 12.28 $\mu mol \cdot L^{-1}$, c_2 = 15.07 $\mu mol \cdot L^{-1}$, c_3 = 17.79 $\mu mol \cdot L^{-1}$, c_4 = 20.44 $\mu mol \cdot L^{-1}$ (see Table S1).

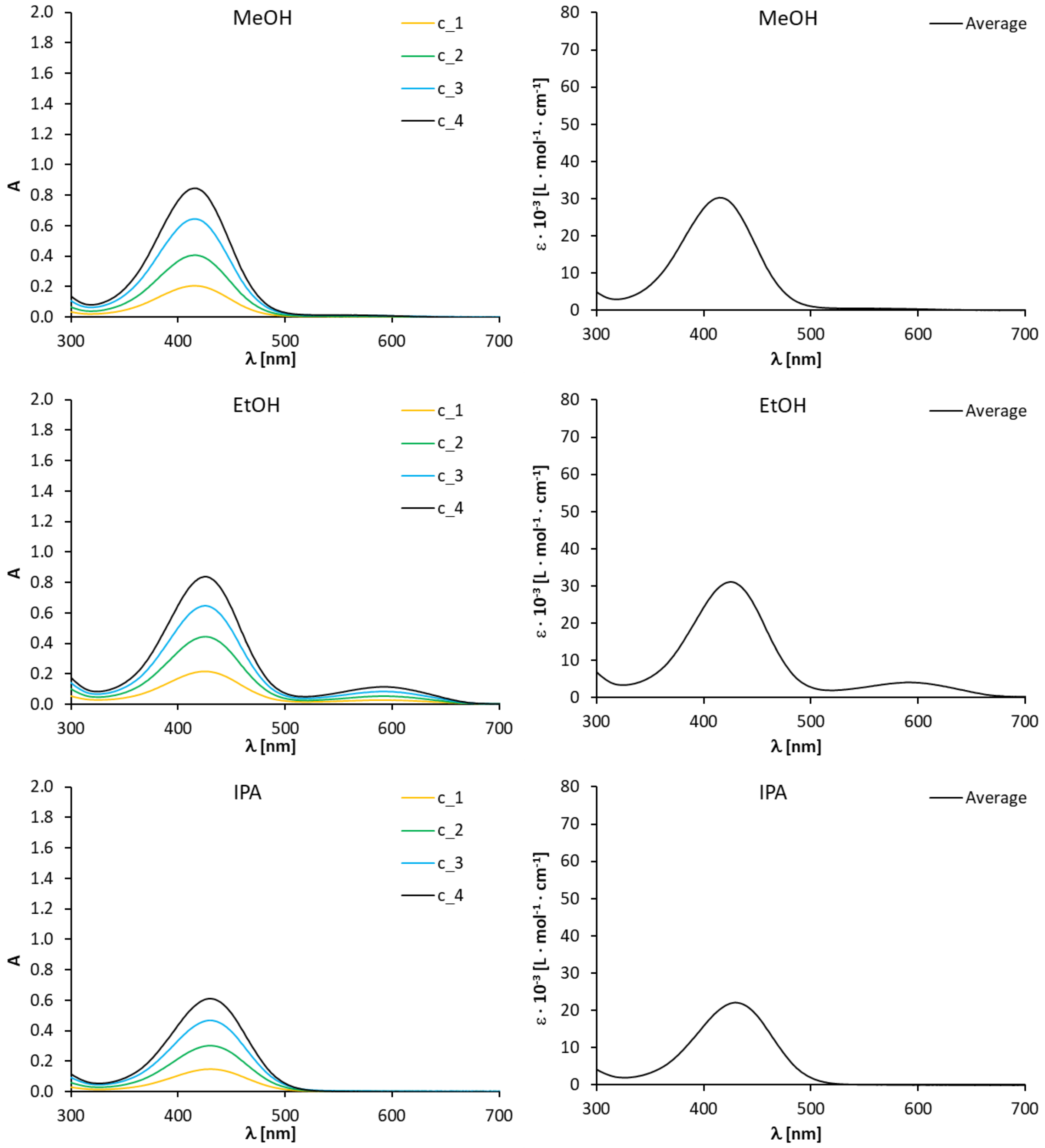

Fig. S51 UV-Vis spectra of stilbazolium dye **5b** in various solvents. Left: Titration experiments. Right: Averaged UV-Vis spectrum obtained from the spectra from titration experiments after their conversion to concentration-independent spectra. The measured concentrations were as follows: c_1 = 12.28 µmol·L^{-1}, c_2 = 15.07 µmol·L^{-1}, c_3 = 17.79 µmol·L^{-1}, c_4 = 20.44 µmol·L^{-1} (see Table S1).

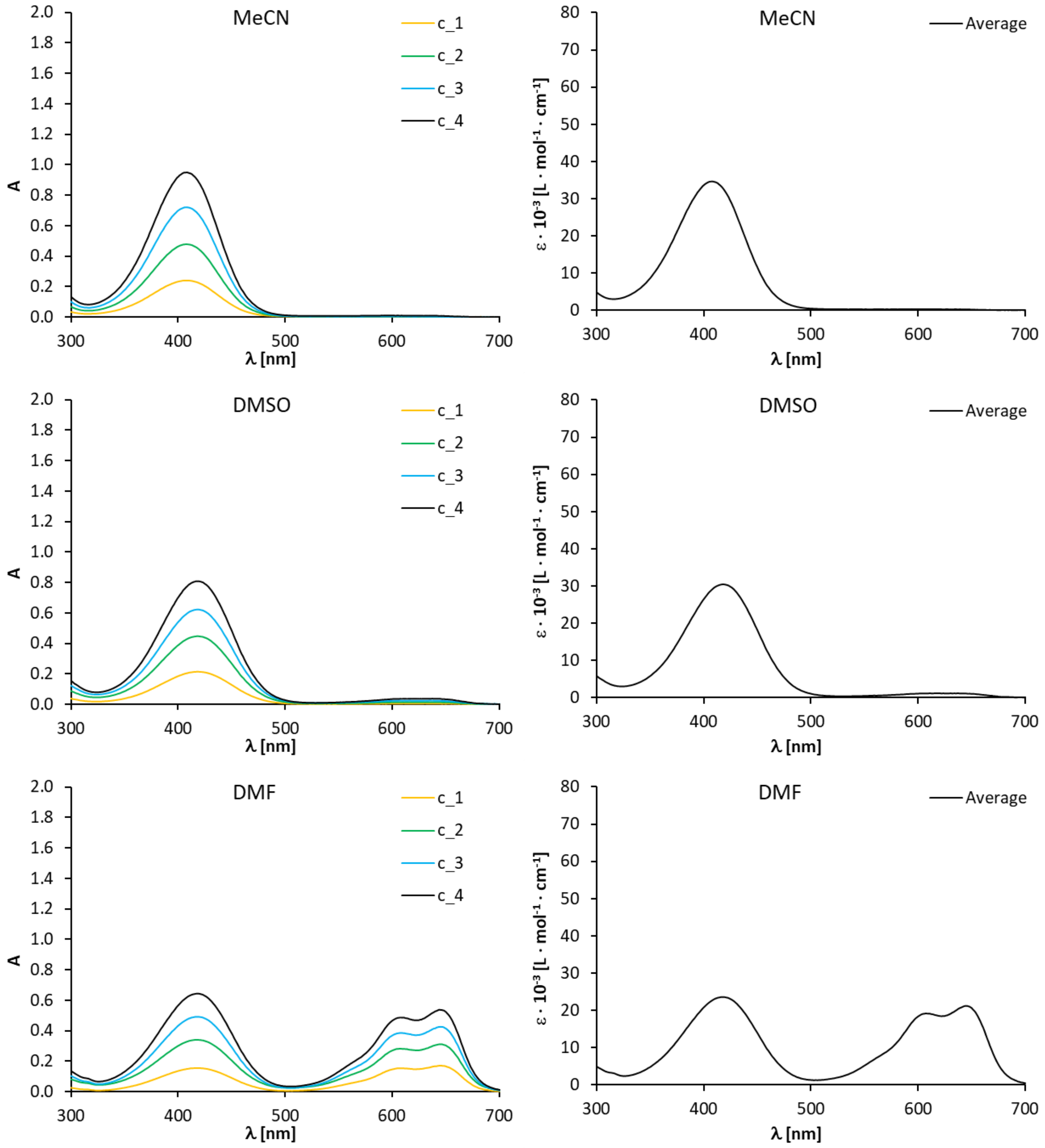

Fig. S52 UV-Vis spectra of stilbazolium dye **5b** in various solvents. Left: Titration experiments. Right: Averaged UV-Vis spectrum obtained from the spectra from titration experiments after their conversion to concentration-independent spectra. The measured concentrations were as follows: c_1 = 12.28 µmol·L^{-1}, c_2 = 15.07 µmol·L^{-1}, c_3 = 17.79 µmol·L^{-1}, c_4 = 20.44 µmol·L^{-1} (see Table S1).

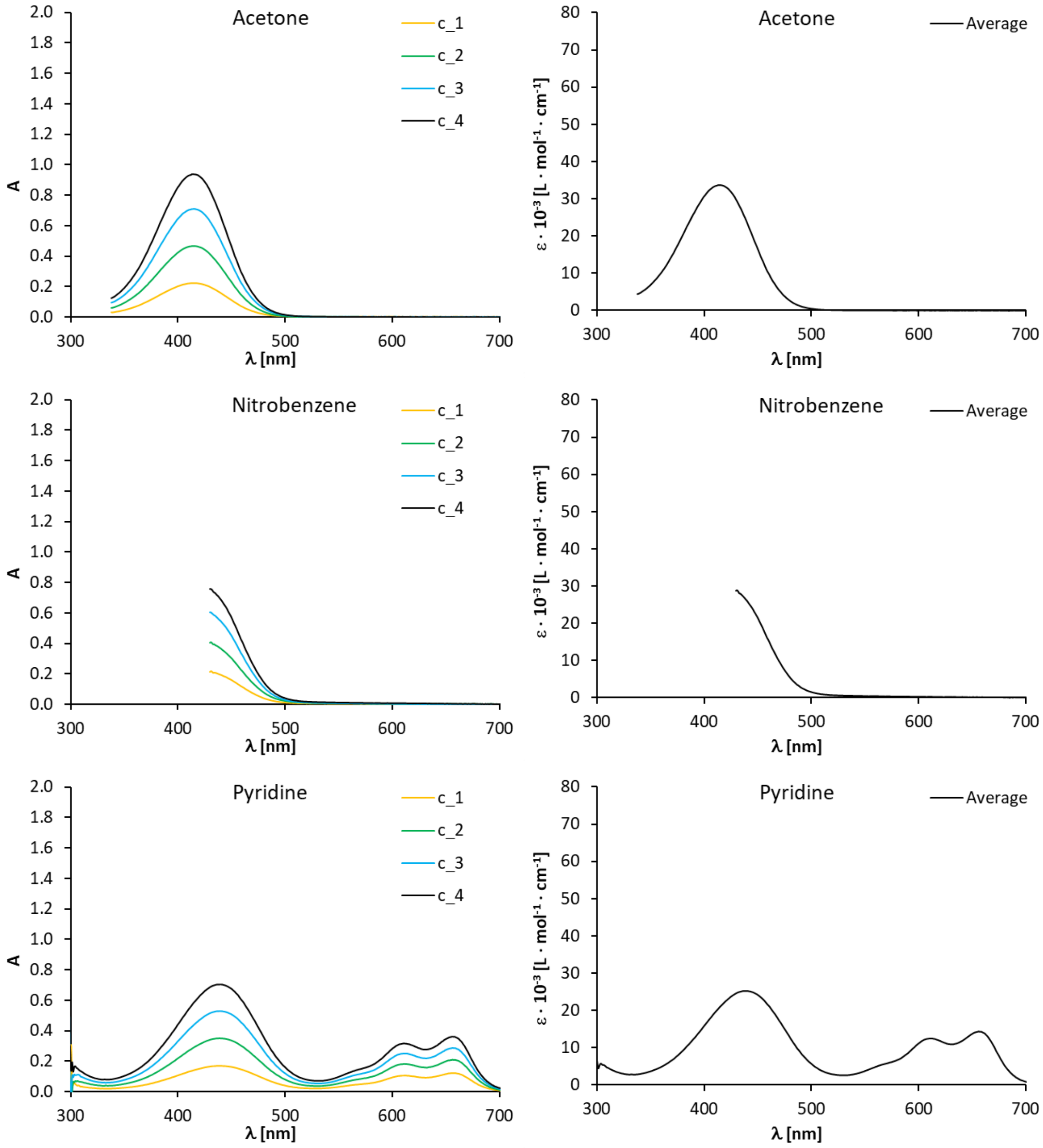

Fig. S53 UV-Vis spectra of stilbazolium dye **5b** in various solvents. Left: Titration experiments. Right: Averaged UV-Vis spectrum obtained from the spectra from titration experiments after their conversion to concentration-independent spectra. In the case of high solvent absorption, the spectrum was clipped (acetone 338 nm; nitrobenzene 430 nm). The measured concentrations were as follows: c_1 = 12.28 µmol·L^{-1}, c_2 = 15.07 µmol·L^{-1}, c_3 = 17.79 µmol·L^{-1}, c_4 = 20.44 µmol·L^{-1} (see Table S1).

*3.2.3 UV-Vis spectra of stilbazolium dye **5a** measured in a solvent mixture*

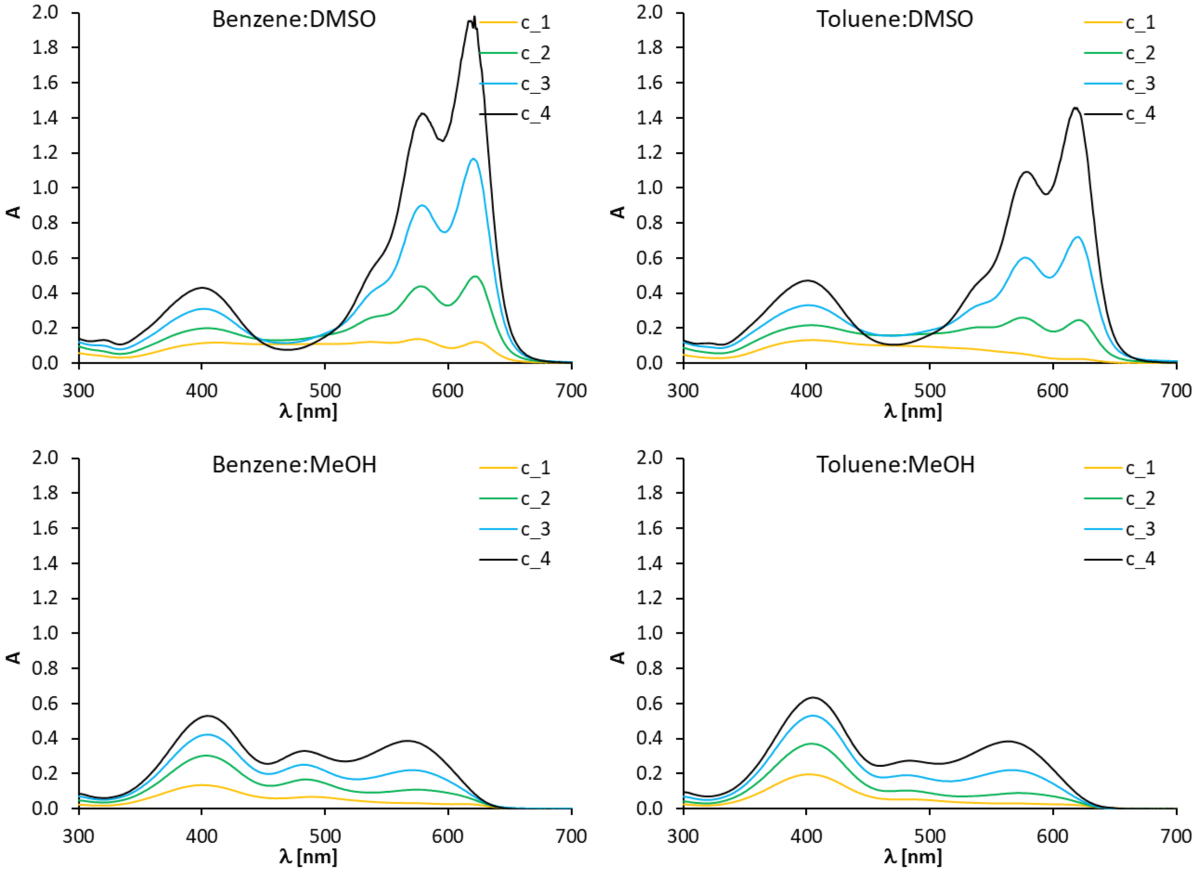

Fig. S54 UV-Vis spectra of stilbazolium dye **5a** in solvent mixtures – titration experiments. The measured concentrations were as follows: c_1 = 12.21 µmol·L^{-1}, c_2 = 15.00 µmol·L^{-1}, c_3 = 17.72 µmol·L^{-1}, c_4 = 20.38 µmol·L^{-1} (see Table S1).

*3.2.3 UV-Vis spectra of stilbazolium dye **5b** measured in a solvent mixture*

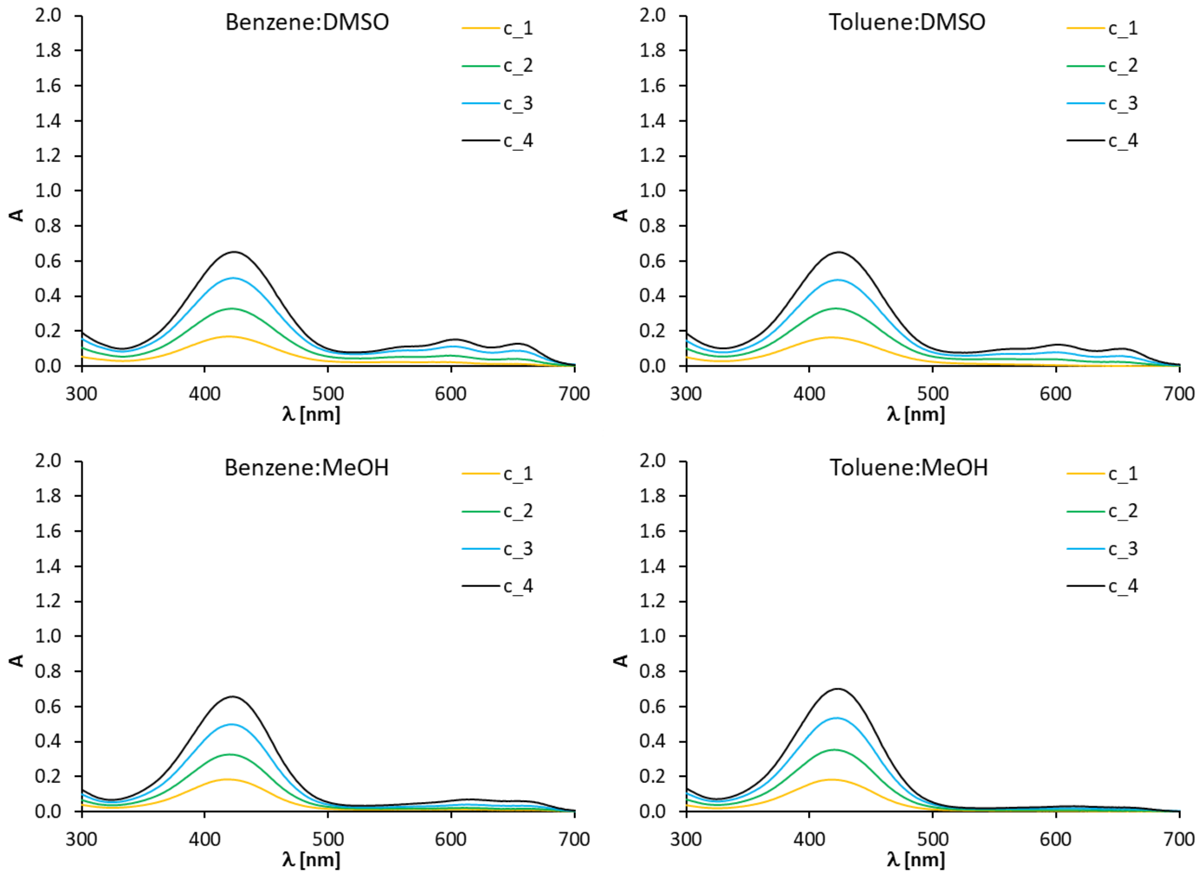

Fig. S55 UV-Vis spectra of stilbazolium dye **5b** in solvent mixtures – titration experiments. The measured concentrations were as follows: c_1 = 12.28 µmol·L^{-1}, c_2 = 15.07 µmol·L^{-1}, c_3 = 17.79 µmol·L^{-1}, c_4 = 20.44 µmol·L^{-1} (see Table S1).

3.3 Fluorescence emission spectra of stilbazolium dyes 5

*3.3.1 Fluorescence emission spectra of stilbazolium dye **5a** measured in a single solvent*

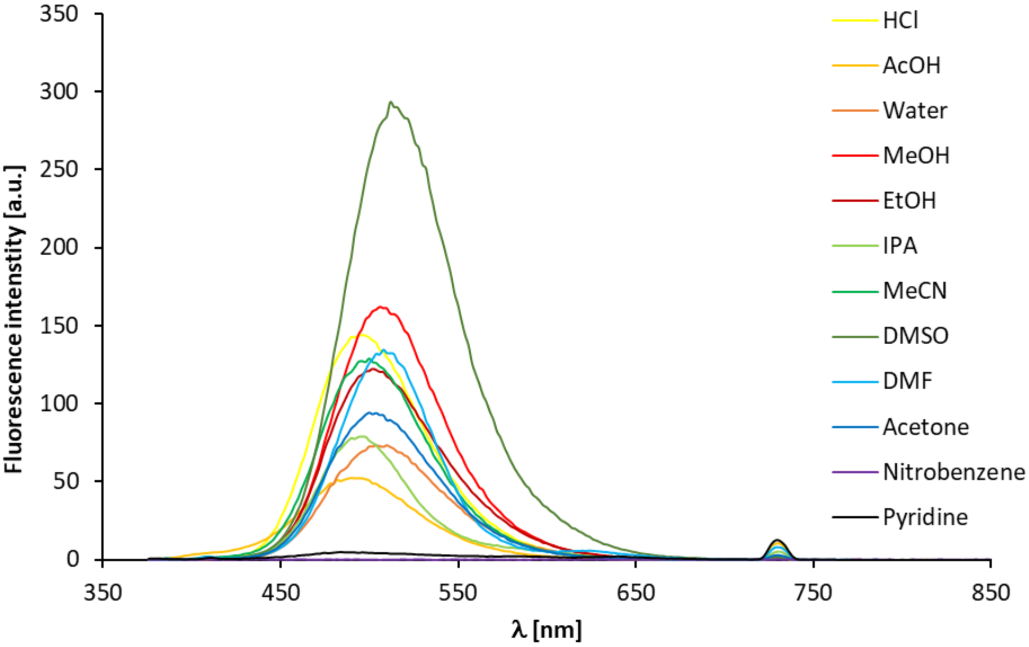

Fig. S56 Fluorescence emission spectra (λ_{ex} = 366 nm) of stilbazolium dye **5a** measured in different solvents. The low intensity band at 732 nm belongs to the second-order band, which is the result of higher-order scattering from the monochromator and is twice the excitation wavelength of 366 nm used.

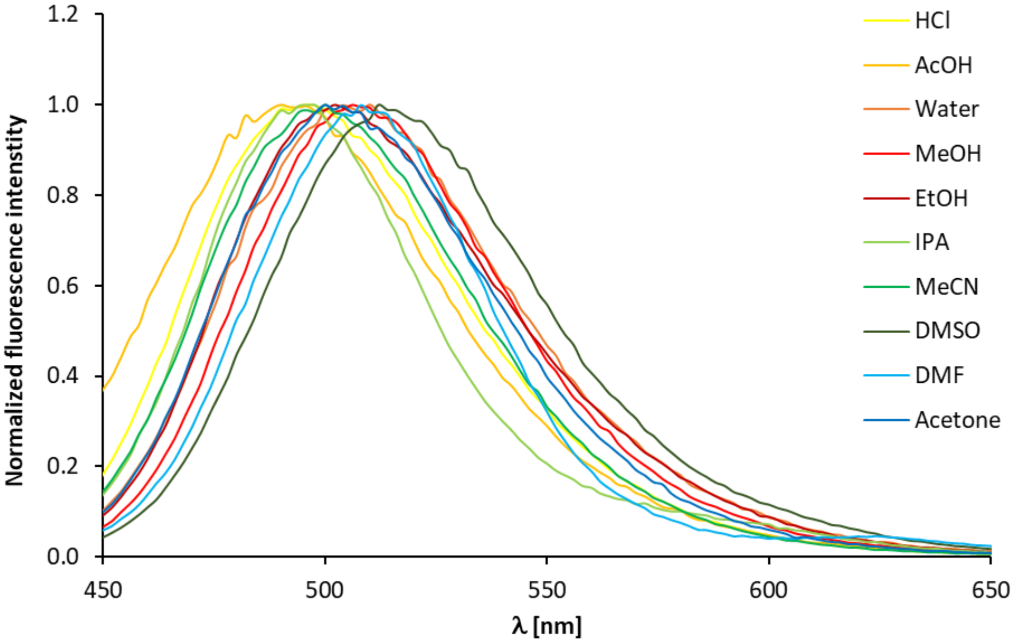

Fig. S57 Section of the normalized fluorescence emission spectra (λ_{ex} = 366 nm) of stilbazolium dye **5a** measured in different solvents (spectral region of 450-650 nm). Normalized spectra of nitrobenzene and pyridine are not shown due to low fluorescence emission intensity.

*3.3.2 Fluorescence emission spectra of stilbazolium dye **5b** measured in a single solvent*

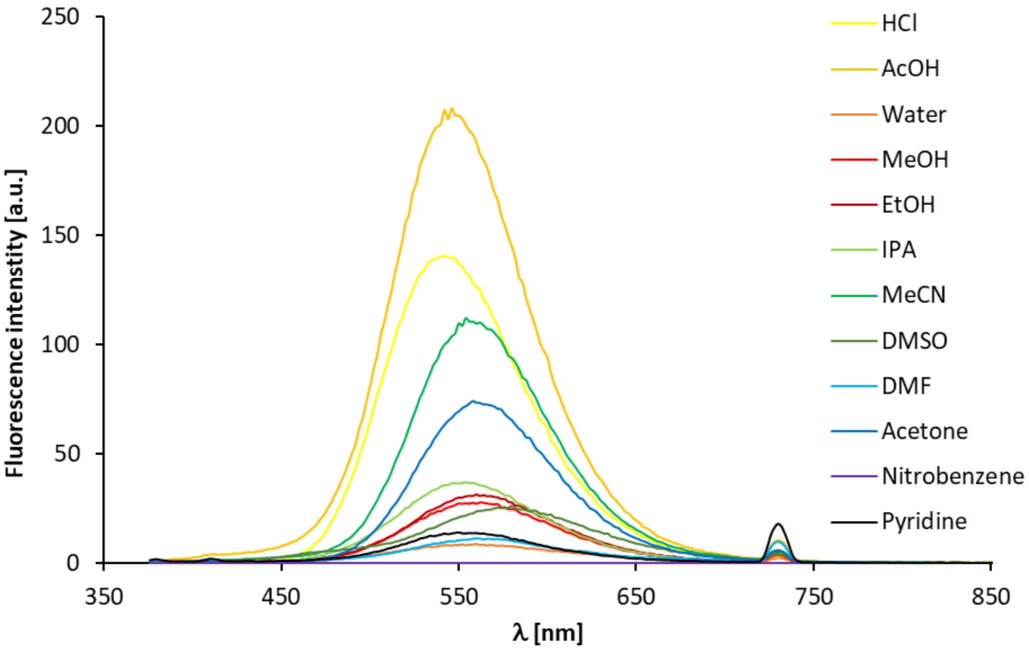

Fig. S58 Fluorescence emission spectra (λ_{ex} = 366 nm) of stilbazolium dye **5b** measured in different solvents. The low intensity band at 732 nm belongs to the second-order band, which is the result of higher-order scattering from the monochromator and is twice the excitation wavelength of 366 nm used.

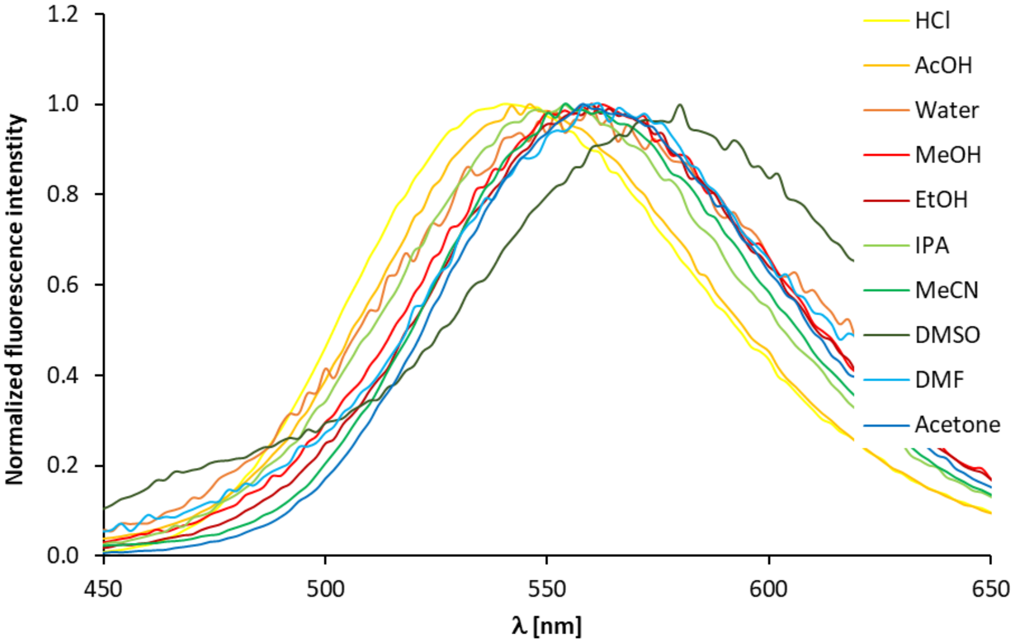

Fig. S59 Section of the normalized fluorescence emission spectra (λ_{ex} = 366 nm) of stilbazolium dye **5b** measured in different solvents (spectral region of 450-650 nm). Normalized spectra of nitrobenzene and pyridine are not shown due to low fluorescence emission intensity.

*3.3.3 Fluorescence emission spectra of stilbazolium dye **5a** measured in a solvent mixture*

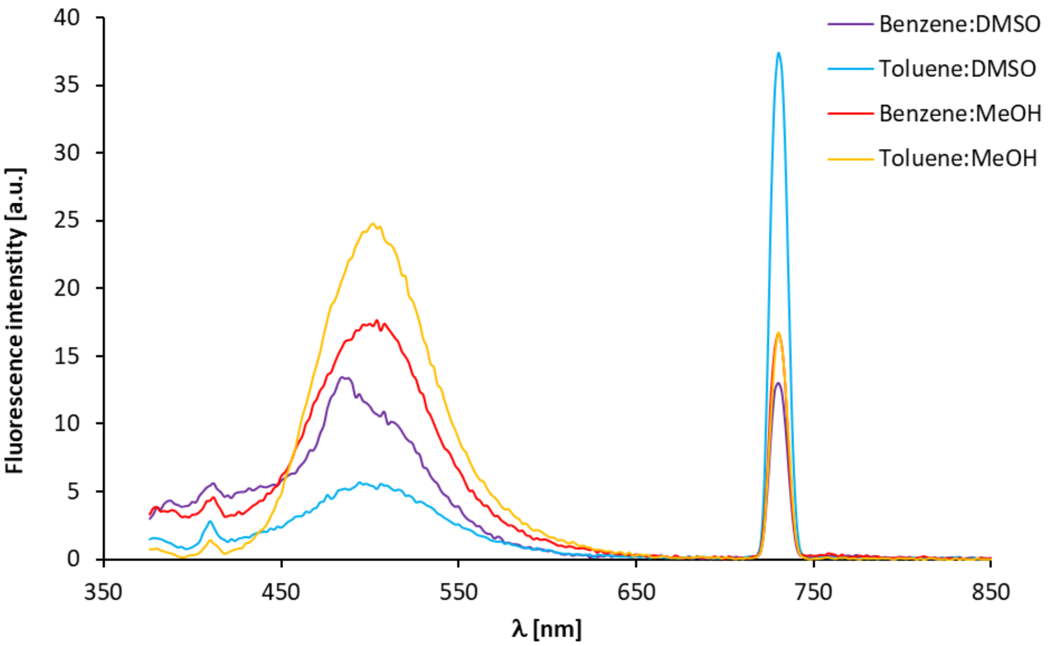

Fig. S60 Fluorescence emission spectra (λ_{ex} = 366 nm) of stilbazolium dye **5a** measured in solvent mixtures. The band at 732 nm belongs to the second-order band, which is the result of higher-order scattering from the monochromator and is twice the excitation wavelength of 366 nm used.

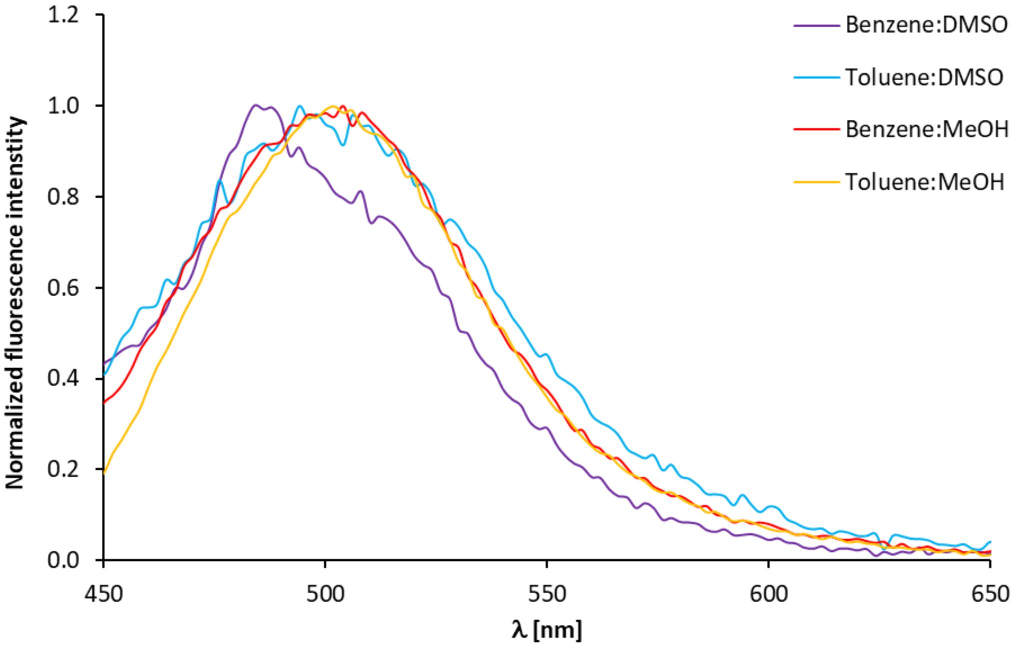

Fig. S61 Section of the normalized fluorescence emission spectra (λ_{ex} = 366 nm) of stilbazolium dye **5a** measured in solvent mixtures (spectral region of 450-650 nm).

*3.3.4 Fluorescence emission spectra of stilbazolium dye **5b** measured in a solvent mixture*

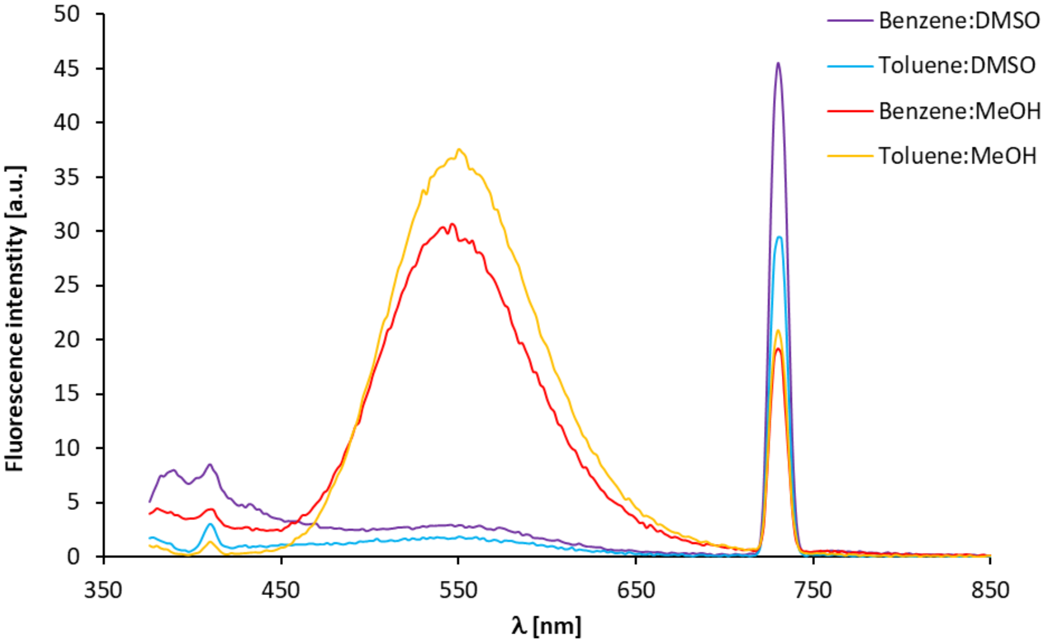

Fig. S62 Fluorescence emission spectra (λ_{ex} = 366 nm) of stilbazolium dye **5b** measured in solvent mixtures. The band at 732 nm belongs to the second-order band, which is the result of higher-order scattering from the monochromator and is twice the excitation wavelength of 366 nm used.

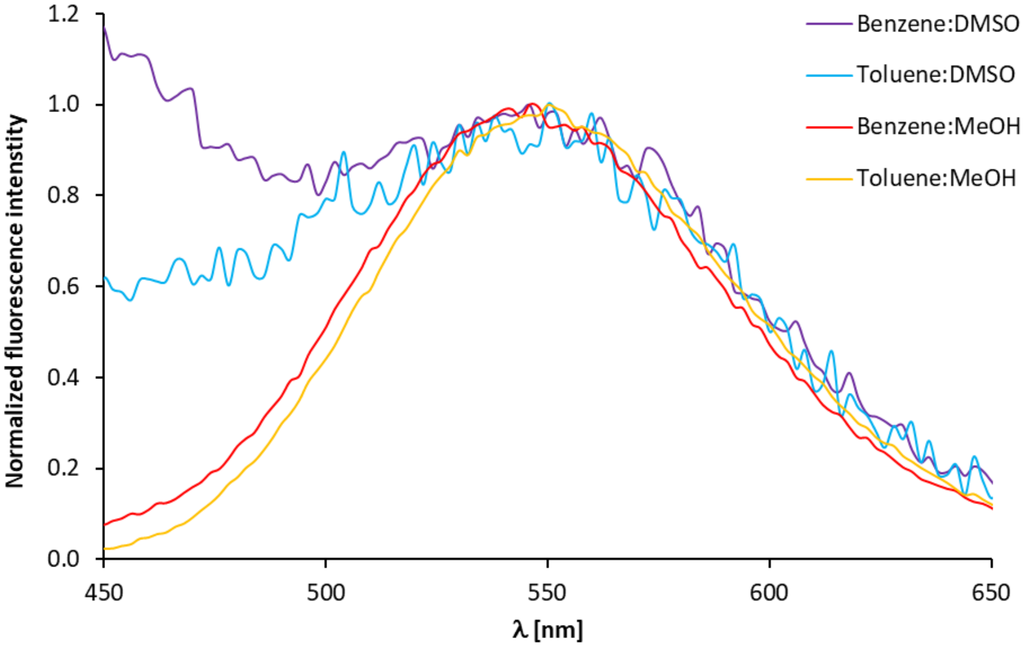

Fig. S63 Section of the normalized fluorescence emission spectra (λ_{ex} = 366 nm) of stilbazolium dye **5b** measured in solvent mixtures (spectral region of 450-650 nm).

3.4 Colorimetric evaluation of stilbazolium dyes 5 in a solvent mixtures

*3.4.1 Colorimetric evaluation of stilbazolium dye **5a***

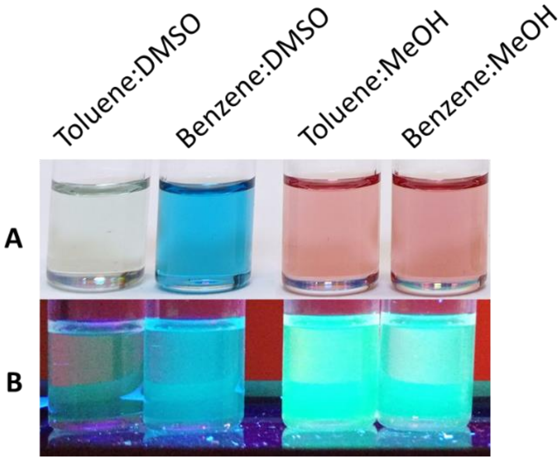

Fig. S64 Colorimetric evaluation of stilbazolium dye **5a** – solutions in a solvent mixture. The colors of the measured solutions of **5a** (c = 20.38 μmol·L^{-1}) under daylight (**A**) and under UV illumination (B, λ_{ex} = 366 nm).

*3.4.2 Colorimetric evaluation of stilbazolium dye **5b***

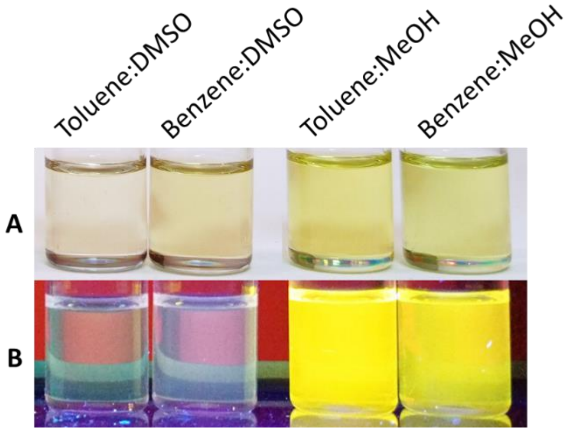

Fig. S65 Colorimetric evaluation of stilbazolium dye **5b** – solutions in a solvent mixture. The colors of the measured solutions of **5b** (c = 20.44 μmol·L^{-1}) under daylight (**A**) and under UV illumination (B, λ_{ex} = 366 nm).

4. Fluorosolvatochromic evaluation of stilbazolium salt 12a and stilbazolium dye 13a

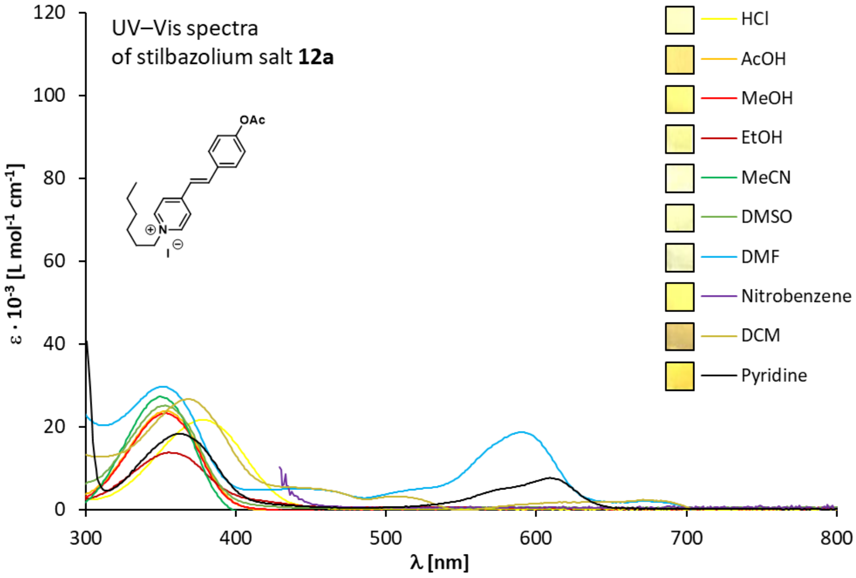

Fig. S66 UV–Vis spectra of stilbazolium salt **12a** measured in different solvents. The legend shows the colors of the stock solutions in glass vials under daylight. Each vial contains the same concentration of the stilbazolium salt **12a** (c = 11 μmol·L^{-1}).

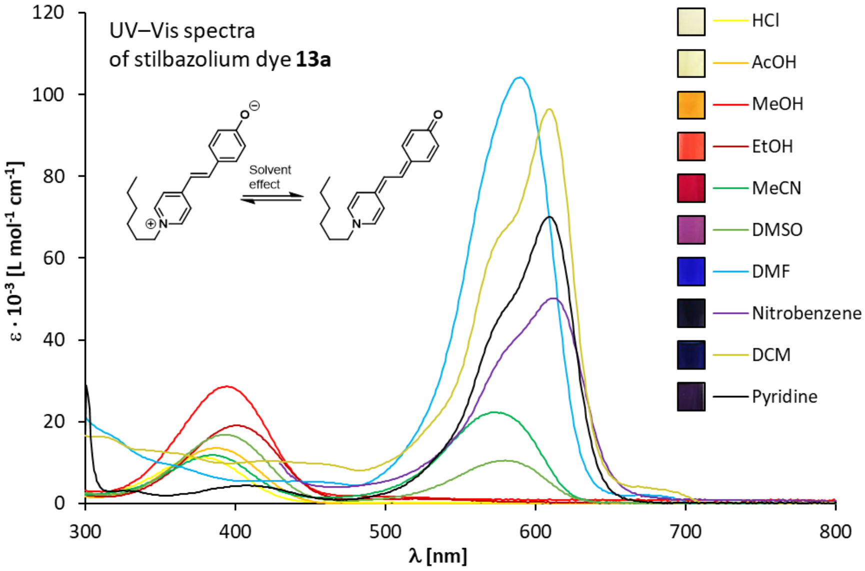

Fig. S67 UV–Vis spectra of stilbazolium dye **13a** measured in different solvents. The legend shows the colors of the stock solutions in glass vials under daylight. Each vial contains the same concentration of the stilbazolium salt **13a** (c = 11 μmol·L^{-1}).

5. Elemental analysis of modified silicas 10

Table S2: Results of elemental analysis of modified silicas **10.**

Sample	weight [mg]	%N	%C	%H	%S
**10a** (No.1)	54.0680	0.13	2.05	1.04	0.01
**10a** (No. 2)	45.2370	0.14	2.07	1.05	0.01
**10a (Average)**	**49.6525**	**0.14**	**2.06**	**1.05**	**0.01**
**10b** (No. 1)	45.7250	0.18	2.25	1.03	0.01
**10b** (No. 2)	45.0570	0.19	2.23	1.05	0.01
**10b (Average)**	**45.3910**	**0.18**	**2.24**	**1.04**	**0.01**

Table S3: Calculation of the nitrogen content bound on the surface of modified silicas **10.**

Sample	weight [mg]	%N	m_N [mg]	n_N [mmol]
**10a** (Average)	49.6525	0.14	0.06951	**0.004962**
**10b** (Average)	45.3910	0.18	0.08170	**0.005832**

The substance amount of nitrogen should directly correspond to the substance amount of bound stilbazolium dye.

6. Colorimetric evaluation of modified silicas 10

Colorimetric evaluation of solvatochromic response in a flow-through arrangement

From the NMR cuvette, rubber hose and parafilm, a device allowing continuous flow of solvents was created by layering cotton, glass wool and solvatochromic silica **10a**. Fig. S68 shows the resulting cuvette before use and the color change of silica **10a** after testing a continuous flow of three different solvents. First, the cuvette was filled with distilled water, then solvent was contiually changed to methanol, then tetrahydrofuran, and finally washed again with distilled water to verify the reversibility of the color change. The color change was visible by naked-eye detection and fully reversible. This arrangement eliminates the need for regeneration of the solvatochromic material and is therefore suitable for repeated use.

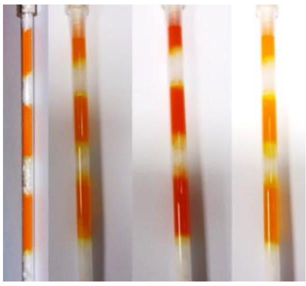

Fig. S68 From left: prepared cuvette with dry solvatochromic silica **10a**, cuvette with a flow of distilled water (orange), methanol (red) and tetrahydrofurane (yellow).

7. Supplemental references

[S1] Garcia-Guzman, C.; Fernandez, A.; Avlonitis, N.; Bradley, M.; Vendrell, M. Red-Fluorescent Activatable Probes for the Detection of Hydrogen Peroxide in Living Cells. *Comb. Chem. High Throughput Screening* **2016**, *19*, 5, 353–361

[S2] Lippert, E.; Lüder, W.; Moll, F.; Nägele, W.; Boos, H.; Prigge, H.; Seibold-Blankenstein, I. Umwandlung von Elektronenanregungsenergie. *Angew. Chem.* **1961**, *73*, 695–706

[S3] Dennis, K. J.; Luong, T.; Reshwan, M. L.; Minch, M. J. Isomerizable *N*-alkylmerocyanine dyes as probes of micellar solubilization sites. *J. Phys. Chem.* **1993**, *97*, 31, 8328–8335

[S4] Kumar, U.; Kato, T.; Frechet, J. M. J. Use of intermolecular hydrogen bonding for the induction of liquid crystallinity in the side chain of polysiloxanes. *J. Am. Chem. Soc.* **1992**, *114*, 17, 6630–6639
